# Supplementary material for: Cognitive Performance in Relation to Systemic and Brain Iron at Perimenopause
Source: Nutrients. 2025 Feb 20;17(5):745. doi: 10.3390/nu17050745 (PMC11901746; doi:10.3390/nu17050745)
Supplement: Supplementary file 1 [file nutrients-17-00745-s001.zip › main.pdf]

Supplementary Materials: Cognitive Performance in Relation to Systemic and Brain Iron at Perimenopause

Amy L. Barnett[1], Michael J. Wenger[2]*, Pamela Miles[3], Dee Wu[4], Zitha Redempta Isingizwe[5], Doris M. Benbrook[6], and Han Yuan[7]

1. Methodological details

1.1. Face/name associative memory task (FNAM)

The FNAM measures episodic memory for the association of faces with names and putative occupations and has been shown to show some discriminative sensitivity to the presence of mild cognitive impairment [1–3]. The version of the FNAM used in the present study was based on that used by Rubiño and colleagues [4]. The FNAM consisted of three phases: a learning phase followed by an initial recall phase, and a final delayed recall phase, which occurred approximately two hours after the initial learning and recall phases (at the end of the testing session).

In the initial learning phase, participants were first shown photographs of a set of 24 women with each image presented for 2 sec. Supplementary Figure S1 shows example images from each phase of the task. Facial images used as stimuli in the face name associative memory task were obtained from publicly available websites and were resized and converted to grayscale and were arbitrarily and randomly paired with a set of names and a set of occupations. The facial images subtended 4.8° of visual angle, both horizontally and vertically. Order of presentation in all phases was randomized across participants.

Participants self-initiated the sequence of presentations in each of the learning phases. After the initial presentation of all the faces, each face was shown again, now with a name directly below the photograph (Supplementary Figure S1b). Participants were instructed to study each face and name pairing for 2 sec. After studying the faces and names, participants were then tested on their memory for the association of faces and names. On each test trial, participants were presented with a studied face along with two names, below and to the left and right of the face; one of the names was the originally studied name and one was

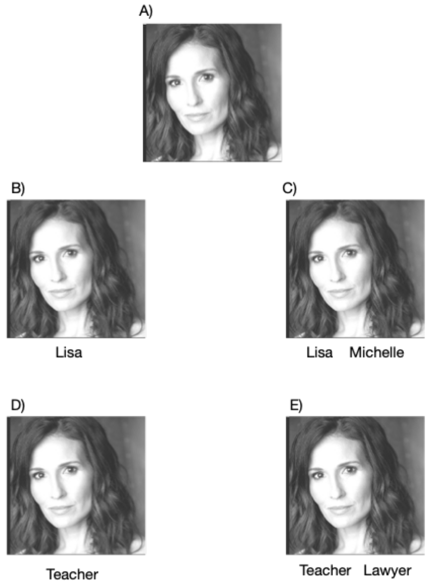

Figure S1. Examples of the presentation of stimuli during the study and test phases of the FNAM: (a) initial presentation of the face alone, (b) presentation of the face with its associated name, (c) presentation of the face with two alternative names at test, (d) presentation of the face with its associated occupation, (e) presentation of the face with two alternative occupations at test.

a novel lure (Supplementary Figure S1c). Participants pressed the z key if they thought the correct name was presented below the photograph on the left side of the screen and pressed the m key if the correct name was on the right side. Participants were given 2.5 seconds to respond to each photograph, and feedback was given for 1.5 sec on their choice after a 1 sec delay.

In the next stage, participants learned the occupations of each of the 24 women they had previously seen. Participants self-initiated the set of presentations. Each face appeared on the screen for 2 sec with an occupation label (Supplementary Figure S1d) listed directly below the face. Following the presentation of all the face-occupation pairings, participants were tested on their immediate memory for those associations. The test trials followed the same procedure used to test the initial face-name associations (Supplementary Figure S1e). After all the other behavioral tests were completed, the final (delayed, after approximately 2 hours) test of memory for the face-name and face-occupation associations was performed using the same procedures as were used for the initial tests.

1.2. Probabilistic selection task (PST)

The PST measures positive and negative reinforcement learning, which can be affected by variations in levels of dopamine [5,6]. The stimuli in the PST were characters from the Nepalese Devanagari script presented in pairs with each member of the pair subtending 4.8° horizontally and vertically, and each stimulus was offset 75 pixels horizontally from the center of the screen.

The PST involved two phases: a training phase and a testing phase. In the training phase, participants were presented with three pairs of stimuli (see Supplementary Figure S2) and needed to learn which member of each pair was rewarded more frequently. One member of each pair was rewarded more frequently than the other, and the differential reward rate was probabilistic. For the first pair of stimuli (the AB pair), A was rewarded 80% of the time while B was rewarded 20% of the time. For the second pair (the CD pair), the relative rates of reward were 70% vs. 30%, and for the third pair (the EF pair), the relative rates of reward were 60% vs. 40%. These pairings were presented in blocks, with each block containing three presentations of each pairing in random order. Each trial in each block was self-initiated, followed by the presentation of a fixation cross with an exposure duration drawn from an exponential distribution with a mean of 750 ms, censored at 500 and 1000 ms. The test pairing for the trial was then presented with left/right location of each member of the pair determined randomly on each trial. The pair was presented for up to 3 sec or until the participant made a choice. Participants were instructed to choose the rewarded member of the pair using the z key for the member on the left and the m key for the right. Visual feedback ('correct' or 'incorrect') was presented for 1.5 seconds after a 0.7 sec delay. Participants exited the training phase if either they met criterion levels of preference for the three pairs (choosing A 65% of the time in AB pairings, choosing C 60% of the time in CD pairings, and choosing E 50% of the time in EF pairings) in a given block, or they completed three blocks of training trials.

The testing phase involved the presentation of all possible pairings of the six stimuli and tested the choice preferences learned in the training phase from two perspectives. The first was with respect to the extent to which learning to choose stimulus A (the most rewarded stimulus) and learning to avoid stimulus B (the least rewarded stimulus) generalized to other pairings. The other was with respect to the amount of conflict present in the tested choice. This took two forms: conditions of high conflict, involving a choice between either two frequently rewarded stimuli (A with C or E, C with E) or two rarely rewarded stimuli (B with D or F, D with. F); and conditions of low conflict, involving a choice between a frequently rewarded and an infrequently rewarded stimulus (A with D or F, B with C or E). Each of the six possible pairings was repeated 15 times, with the order of presentation determined randomly. The trial events in the testing phase were identical to those in the training phase, with the exception that no feedback was provided.

Table S1. Parameters for the Gaussian distributions used to generate stimuli for the RBCL.

	Orientation (degrees)		Spatial frequency (cycles/100 pixels)	
Category	μ	σ	μ	σ
A	54	8	4	1
B	54	8	6	1
C	76	8	4	1
D	76	8	6	1

1.3. Rule-based category learning task (RBCL)

The RBCL is drawn from a literature on how the learning of different category structures is hypothesized to require the involvement of two distinct forms of learning and memory [7–10]. One of these is procedural learning, which is dependent on implicit learning of stimulus response associations, and the second is declarative learning, which is dependent on learning explicit, verbalizable rules. There are data suggesting the involvement of DA in both types of learning, with recent work [11] suggesting that DA functions in declarative learning with respect to the maintenance and switching of response rules and in procedural learning as a training signal.

In the RBCL, participants were presented with Gabor patches varying on two dimensions: spatial frequency and orientation. Stimuli subtended 2.8° both horizontally and vertically and were centered on the screen.Participants were required to learn the stimuli and place them in one of four categories, labeled A, B, C, and D. A total of 100 stimuli were generated for each category by drawing from Gaussian distributions parametrized for spatial frequency and orientation; see Supplementary Table S1 for these means and standard deviations of these distributions and see Supplementary Figure S3 for example stimuli from each category. The rule for determining category was a simple verbalizable rule, such that if orientation and spatial frequency were both low, the stimulus belonged to category A, and if orientation and spatial frequency were both high, the stimulus belonged to category D, etc..

At the beginning of the task, participants were shown examples of each category without any information about the rule that determined its category membership. Participants self-initiated each trial using the space bar in response to a circle that appeared on the screen. A fixation cross with a duration drawn from an exponential distribution with a

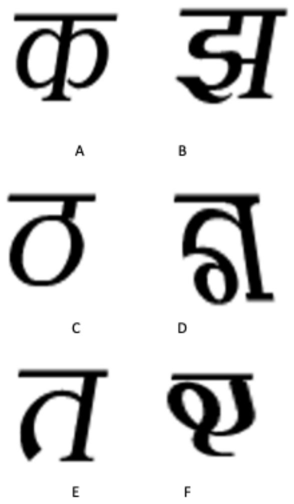

Figure S2. Examples of the presentation of stimuli during the training and test phases of the PST. See text for details of the pairings during the training phase.

mean of 750 ms, censored at 500 and 1000 ms. The test stimulus was presented until a response was made or until 2 s elapsed. Visual feedback was presented for 1.5 s following the participant's choice. The message "... take a short break ... " appeared after every 100 trials. Participants then continued until 400 trials were completed, with stimuli from each of the categories presented in random order 100 times. At the end of the task participants were asked, "For the task you just finished, you should have been making your decisions based on two dimensions. What do you think those two dimensions were? How were you making your decisions?" to check if they could verbalize the rules from the task.

1.4. Visuospatial working memory task (VSWM)

The VSWM was included on the basis of a set of findings [12–14] suggesting that variations in performance on tests of this form of working memory can be related to variations in DA status as well as age [15]. In the current study, visuospatial working memory is dependent on both attention and memory.

In the practice phase, two types of trials were introduced. In all practice trials, after a small circle appeared on the screen, participants were instructed to press the space bar to initiate the trial. A fixation cross with an exposure duration drawn from an exponential distribution with a mean of 750 ms, censored at 500 and 1000 ms, appeared on the screen. This was followed by a study display comprised of 16 white squares arranged in a circle. Three targets (red circles) randomly appeared in each of the 16 squares. Participants were instructed to remember the locations of the three targets. This study display was presented for 1 s followed by a 2 s blank display. The test display then followed, comprised of the 16 white squares (arranged in a circle) with a question mark appearing in one of the squares, prompting the participant to indicate whether a target was present in that square in the study display. Participants gave a positive response with the index finger of their dominant hand and a negative response with the index finger of their non-dominant hand. Participants were given up to 3 sec to respond before the next trial began, and feedback was not presented. Five practice trials were completed in the first set.

In the second set of practice trials, distractors (blue circles) were included along with the targets. The second set of practice trials followed the same process as the first set. Sixteen white squares arranged in a circle appeared on the screen. Three targets (red circles) and two distractors (blue circles) randomly appeared in each of the 16 squares. Participants were instructed to remember the locations of the three targets and to ignore the locations of

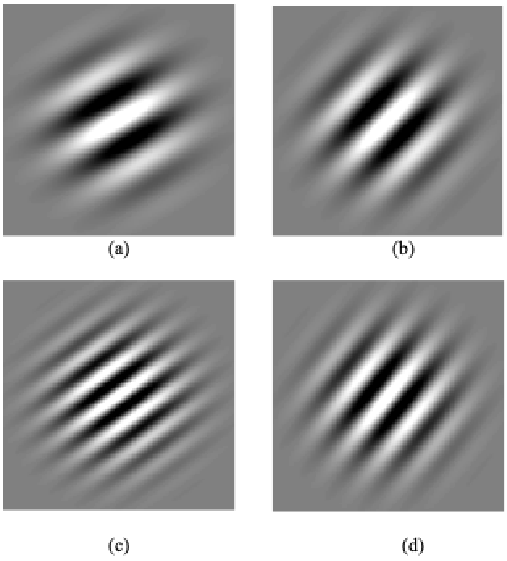

Figure S3. Examples of the stimuli from the four categories used in the RBCL: (a) low spatial frequency, low orientation; (b) low spatial frequency, high orientation; (c) high spatial frequency, low orientation; (d) high spatial frequency, high orientation.

the distractors. This study display was presented for 1 s followed by a 2 s blank display. The test display then followed, comprised of the 16 white squares with a question mark appearing in one of the squares, prompting the participant to indicate whether or not a target was present in that square in the study display. Participants gave a positive response with the index finger of their dominant hand and a negative response with the index finger of their non-dominant hand. Participants were given up to 3 sec to respond before the next trial began, and feedback was not given.

Each trial in the testing phase of the VSWM was self-initiated. After a small circle appeared on the screen, participants were instructed to press the space bar to initiate the trial. A fixation cross with an exposure duration drawn from an exponential distribution with a mean of 750 ms, censored at 500 and 1000 ms, appeared on the screen. This was followed by a study display comprised of 16 white squares arranged in a circle appeared on the screen. A randomly selected set of either three or five squares contained targets (red circles) while another randomly selected set of either zero or two additional squares contained distractors (blue circles). Participants were instructed to remember the locations of the red circles and to ignore the locations of the blue circles (see Supplementary Figure S4 for an example of a study display). This study display was presented for 1 s followed by a 2 s blank display. The test display then followed, comprised of the 16 white squares (arranged in a circle) with a question mark appearing in one of the squares, prompting the participant to indicate whether a target was present in that square in the study display. Participants gave a positive response with the index finger of their dominant hand and a negative response with the index finger of their non-dominant hand. Participants were given up to 3 sec to respond before the next trial began, and feedback was not presented. A total of 96 trials were presented, with half of the trials probing a studied location and half probing an unstudied location.

2. EEG acquisition, pre-processing, and feature extraction

2.1. Acquisition

EG data were collected using high-density (128 channel) electrode nets (Magstim/EGI, Eugene OR). Data were acquired using a 128-channel Net Amps 300 amplifier and were digitized at a sampling rate of 1 kHz. Impedances were kept at or below 50 $K\Omega$ during testing. Data were collected with online filters set at 0.1 Hz (highpass), 70 Hz (lowpass), and 60 Hz (notch).

2.2. Pre-processing

EEG data from all of the tasks and the resting data were pre-processed up to the point of feature extraction using the same set of steps. All pre-processing and feature extraction were done using the EEGLAB toolbox [16] for Matlab (Mathworks, Natick MA) following a recently published pipeline [17]. Data were first filtered using a high-pass filter at 0.5 Hz, a low-pass filter at 90 Hz, and a notch filter at 58-62 Hz. All data from before the start of

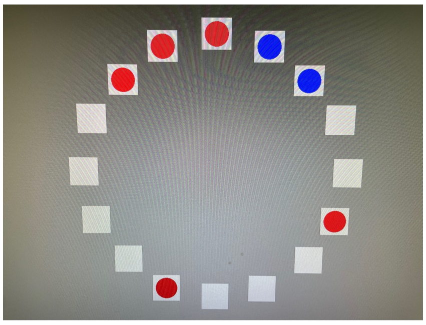

Figure S4. Examples of a study display in the VSWM, containing five targets (red circles) and two distractors (blue circles).

the task and after the end of the task were then removed, bad channels identified by visual inspection were removed, and then bad segments and any additional bad channels were removed using the function `pop_clean_rawdata` in EEGLAB. Data were then re-referenced to the mean. An independent component analysis was then done to identify artifacts such as muscle, eye, and cardiac artifacts [18]. The EEGLAB functions `pop_iclabel` and `pop_icflag` were then used to flag and remove components identified as artifacts. Missing channels were then interpolated.

2.3. Feature extraction: FNAM

The features extracted for the FNAM data were based on those in Guo et al. 2005 and Mitchell et al. 2016. Pre-processed data were low-pass filtered at 30 Hz and data were restricted to those recorded at electrodes 11, 129, 62, and 75, corresponding to Fz, Cz, Pz, and Oz, for the testing phase of the task. Data were then epoched from 200 ms preceding to 2500 ms following the onset of the test stimulus and baseline corrected. Average waveforms for remembered and forgotten face/name and face/occupation associations were then calculated for each participant. From these average waveforms, mean amplitudes were then calculated for three time periods following the onset of the test stimulus: 200-400 ms (positive amplitudes), 400-600 ms (negative amplitudes), and 600-800 ms (positive amplitudes).

2.4. Feature extraction: PST

Response-locked and feedback-locked features for the PST data were based on those in Frank et al. 2005, Larson et al. 2015, and Schmid et al. 2018. The response-locked features were error-related negativity (ERN) and positivity (Pe), and correct-related negativity (CRN). The feedback-locked features were the feedback-related negativity (FRN) for correct and incorrect responses. All features were extracted from the data from the test phase of the task. Pre-processed data were low-pass filtered at 15 Hz and then were epoched from 200 ms prior to either the response or the feedback to 500 ms after the response or 800 ms after the feedback. Data for the ERN and CRN were drawn from a cluster of electrodes surrounding Cz (4, 5, 11, 12, 16, and 19, following [21]). Data for the Pe were drawn from a separate set of electrodes (4, 55, 61, 62, 78, and 79, following [22]). Data for the FRN were drawn from a set of three electrodes (6, 7, and 106, following [23]). Average waveforms were calculated for each participant and baseline corrected.

The ERN was calculated as the difference between the maximum negative amplitude within 200 ms of the onset of the stimulus and the preceding maximum positive amplitude. The Pe was calculated as the difference between the maximum positive amplitude within 200 ms of the onset of the stimulus and the preceding maximum negative amplitude. The CRN was calculated as the difference between the maximum negative amplitude within 400 ms of the onset of the stimulus and the preceding maximum positive amplitude. The FRN for both correct and incorrect responses was calculated as the difference between the maximum negative amplitude in the interval running from 100-300 ms from the onset of the feedback and the maximum positive amplitude preceding it.

2.5. Feature extraction: RBCL

Stimulus- and feedback-locked features for the RBCL data were based on those in Rabi et al. 2018. Stimulus-locked features were obtained from electrodes 58, 59, 62, 91, and 96, while feedback-locked features were obtained from electrodes 11, 24, 36, 54, 62, 79, 104, and 124. Pre-processed data were low-pass filtered at 30 Hz and then were epoched from 200 ms prior to 800 ms after the onset of either the stimulus or the feedback. The data were averaged separately for correct and incorrect responses for each participant and then baseline corrected. The stimulus-locked P300 was calculated as the mean amplitude in the interval running from 300-600 ms after the onset of the stimulus. The feedback-locked late positive slow wave was calculated as the mean amplitude in the interval running from 300 to 650 ms following the onset of the feedback.

Table S2. Descriptive statistics for the behavioral data from the FNAM.

Task	DV	Retention interval	M	SE	min	max
Face/name	P(C)	Immediate	0.81	0.02	0.58	1.00
		Delayed	0.74	0.03	0.4	0.92
	RT (ms)	Immediate	1402	57	998	1864
		Delayed	1451	47	1037	1837
Face/Occupation	P(C)	Immediate	0.86	0.02	0.46	1.00
		Delayed	0.85	0.02	0.46	0.96
	RT (ms)	Immediate	1418	46	813	1981
		Delayed	1394	47	1141	1788
Delayed recognition	Hit Rate		0.62	0.04	0.08	0.96
	False alarm rate		0.14	0.19	0.04	0.33
	d'		1.5	0.2	-0.72	2.91
	c		0.41	0.06	-0.29	1.18
Old items, correct responses	RT (ms)		1351	59	934	1981
New items, correct responses	RT (ms)		1593	59	1115	2184

2.6. Feature extraction: VSWM

Features for the VSWM data were based on those in Knott et al. 2004. Pre-processed data were low-pass filtered at 12 Hz, the data for correct and incorrect responses were epoched from 200 ms before to 1000 ms after the onset of the stimulus, averaged for each participant, and then baseline corrected. The P300 for each trial type was calculated as the maximum positive amplitude in the interval running from 250 to 800 ms following the onset of the stimulus in three electrodes (11, 55, and 62).

2.7. Blink rates

Task-related and spontaneous blink rates were extracted from each of the tasks and the resting period using the BLINKER plug-in for EEGLAB [25].

3. MRI estimates of regional iron deposits

Our approach to obtaining estimates of regional brain iron deposits was based on that used in [26–28]. Specifically, we determined, for each participant across all ROIs, the intensity value that represented the lowest fifth percentile and isolated those pixels in each slice. We did this to ensure that we had the highest likelihood of isolating iron. We then averaged within and then across slices in each ROI in each hemisphere for each participating. These were the data that were subjected to analyses.

4. Results

4.1. FNAM

4.1.1. Behavioral data

Descriptive statistics for the behavioral data are presented in Supplementary Table S2. The behavioral data (accuracy and RT) were analyzed using 2 (test type: face/name, face/occupation) x 2 (retention interval: immediate, delayed) repeated measures mixed models analyses of variance (ANOVAs), with both factors manipulated within participants and participants as the random factor. Supplementary Table S3 presents the results of these analyses. Cells highlighted in gray indicate statistically significant results. In this case, the only significant effect was a main effect for test type in overall accuracy, with participants being more accurate in recalling face/occupation associations (0.86) than in recalling face/name associations (0.77).

Table S3. Repeated-measures analyses of the behavioral data from the FNAM.

	Factor	F	p
Accuracy, P(C)	Test type (TT)	11.00	0.003
	Retention interval (RI)	3.72	0.066
	TT x RI	2.05	0.166
RT, correct choices, ms	Test type (TT)	0.26	0.613
	Retention interval (RI)	0.09	0.764
	TT x RI	0.86	0.363
RT, Recognition	Old vs. New Items	24.31	< 0.001

4.1.2. Correlations between behavior and iron status

The correlations between the behavioral variables in the FNAM are presented in Supplementary Tables S4 and S5.

Table S4. Correlations between the behavioral variables in the FNAM and the iron status biomarkers (1). Cells highlighted in gray indicate statistically significant results after controlling for the false discovery rate. Note: pctl = percentile.

	Hb		sFt		sFt pctl		Age	
Variable	*r*	*p*	*r*	*p*	*r*	*p*	*r*	*p*
P(C), immediate face/name	0.25	0.255	0.30	0.165	0.46	**0.029**	-0.41	0.052
P(C), delayed face/name	0.35	0.102	0.47	**0.022**	0.44	0.036	-0.10	0.644
P(C), immediate face/occupation	-0.07	0.763	-0.19	0.395	-0.16	0.456	-0.04	0.858
P(C), delayed face/occupation	0.04	0.867	0.01	0.660	0.14	0.515	-0.33	0.126
Hit rate, delayed recognition	0.46	**0.026**	0.48	**0.020**	0.48	**0.022**	-0.15	0.493
False alarm rate, delayed recognition	-0.09	0.693	-0.32	0.139	-0.23	0.284	-0.02	0.929
d', delayed recognition	0.34	0.112	0.49	**0.016**	0.46	**0.026**	-0.11	0.626
c, delayed recognition	-0.47	**0.022**	-0.33	0.121	-0.37	0.080	0.17	0.447
RT, immediate face/name	-0.15	0.498	-0.43	0.038	-0.47	**0.023**	0.28	0.190
RT, delayed face/name	0.20	0.370	-0.51	**0.013**	-0.59	**0.003**	0.11	0.622
RT, immediate face/occupation	0.08	0.716	-0.39	0.066	-0.43	0.042	0.26	0.231
RT, delayed face/occupation	-0.48	**0.020**	-0.08	0.710	-0.20	0.366	0.26	0.237
RT, Old items	0.12	0.573	-0.52	**0.012**	-0.56	**0.006**	0.04	0.839
RT, New items	0.09	0.695	-0.49	**0.018**	-0.60	**0.002**	0.19	0.395

4.1.3. EEG data

Group average wave forms for remembered and forgotten face/name and face/occupation associations at each testing time and at each of the four electrodes are presented in Supplementary Figures S5 and S6. Amplitudes in each of the three time intervals at each of two retention intervals for the two types of association were analyzed using separate 4 (electrode: Fz, Cz, Pz, Oz) × 2 (recall status: remembered, forgotten) repeated measures mixed models with electrode and recall status as fixed factors and participants as random factors. The results of these analyses are presented in Supplementary Table S6.

Table S5. Correlations between the behavioral variables in the FNAM and the iron status biomarkers (2). Cells highlighted in gray indicate statistically significant results after controlling for the false discovery rate.

	WBC		RBC		HCT		MCV		MCH		MCHC		RDW	
Variable	r	p	r	p	r	p	r	p	r	p	r	p	r	p
P(C), immediate face/name	-0.09	0.692	0.09	0.682	0.22	0.309	0.12	0.570	0.18	0.403	0.16	0.477	-0.52	**0.011**
P(C), delayed face/name	-0.07	0.739	0.03	0.883	0.17	0.430	0.16	0.479	0.39	0.068	0.53	**0.009**	-0.45	0.030
P(C), immediate face/occupation	0.03	0.885	0.01	0.974	0.03	0.894	0.02	0.925	-0.10	0.654	-0.24	0.278	0.10	0.660
P(C), delayed face/occupation	0.01	0.957	0.00	0.986	-0.01	0.955	-0.02	0.924	0.04	0.861	0.13	0.569	-0.14	0.513
Hit rate, delayed recognition	-0.15	0.500	0.20	0.358	0.30	0.257	0.07	0.751	0.31	0.155	0.53	**0.010**	-0.52	**0.011**
False alarm rate, delayed recognition	-0.02	0.931	0.17	0.430	0.05	0.817	-0.19	0.388	-0.34	0.111	-0.36	0.094	0.14	0.536
d′, delayed recognition	-0.04	0.873	0.05	0.819	0.17	0.431	0.13	0.559	0.35	0.098	0.51	**0.013**	-0.44	0.033
c, delayed recognition	0.26	0.229	-0.37	0.086	-0.41	0.053	0.05	0.833	-0.10	0.660	-0.31	0.147	0.45	**0.029**
RT, immediate face/name	0.50	**0.015**	0.07	0.758	-0.12	0.583	-0.24	0.268	-0.13	0.210	-0.13	0.561	0.25	0.242
RT, delayed face/name	-0.26	0.231	0.28	0.200	0.13	0.552	-0.24	0.277	-0.12	0.588	0.20	0.354	0.43	0.038
RT, immediate face/occupation	-0.18	0.424	0.44	0.035	0.19	0.380	-0.42	0.046	-0.49	**0.017**	-0.23	0.285	0.29	0.172
RT, delayed face/occupation	0.06	0.796	-0.17	0.436	-0.46	**0.029**	-0.30	0.172	-0.38	0.075	-0.21	0.331	0.36	0.097
RT, Old items	-0.14	0.529	0.17	0.445	0.01	0.981	-0.24	0.266	-0.08	0.717	-0.23	0.285	0.29	0.172
RT, New items	-0.26	0.227	0.28	0.190	0.12	0.581	-0.26	0.231	-0.26	0.222	-0.21	0.331	0.36	0.097

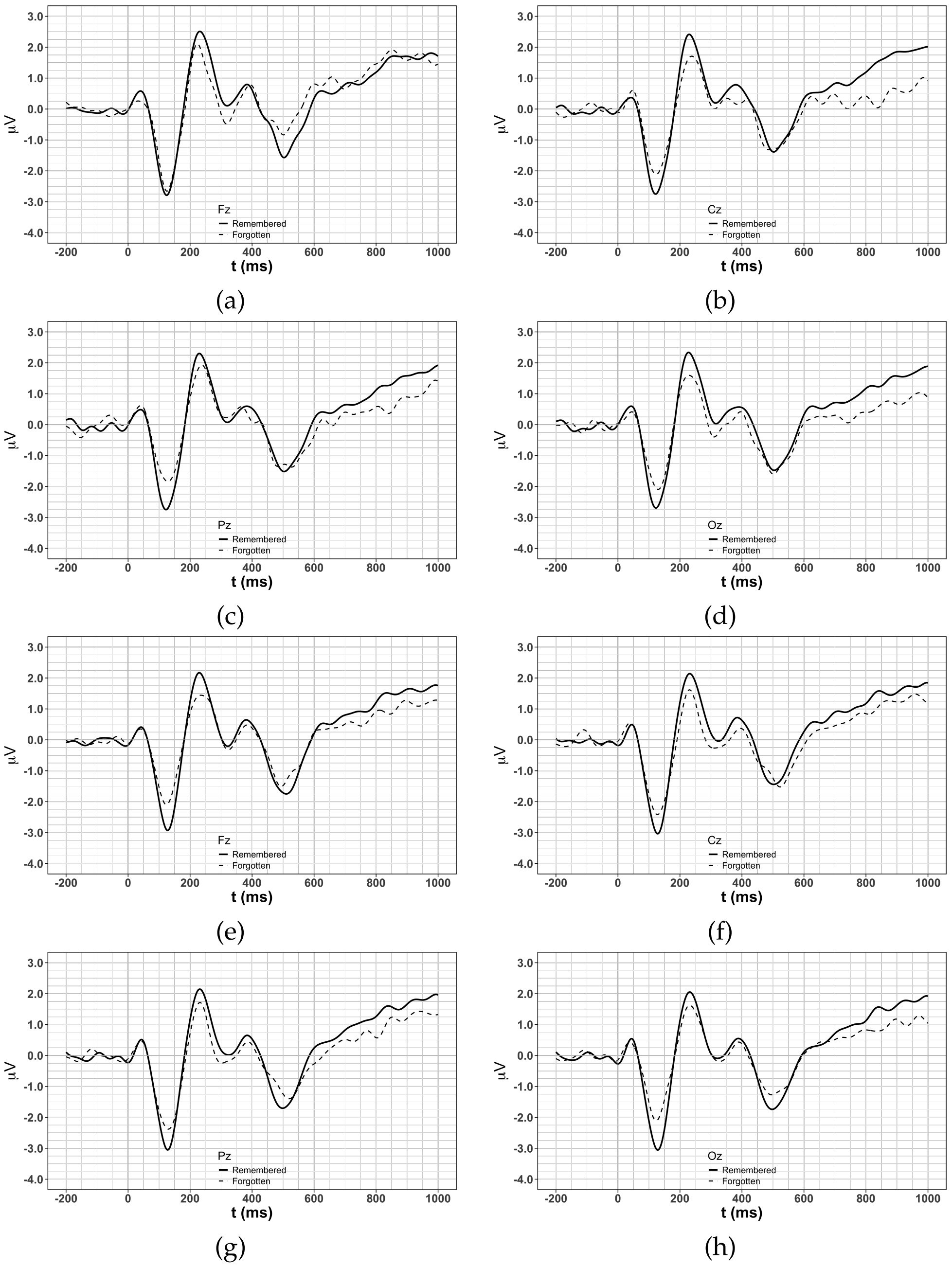

Figure S5. Group average wave forms for remembered and forgotten face/name associations: (a) immediate, electrode Fz; (b) immediate, electrode Cz; (c) immediate, electrode Pz; (d) immediate, electrode Oz; (e) delayed, electrode Fz; (f) delayed, electrode Cz; (g) delayed, electrode Pz; (h) delayed, electrode Oz.

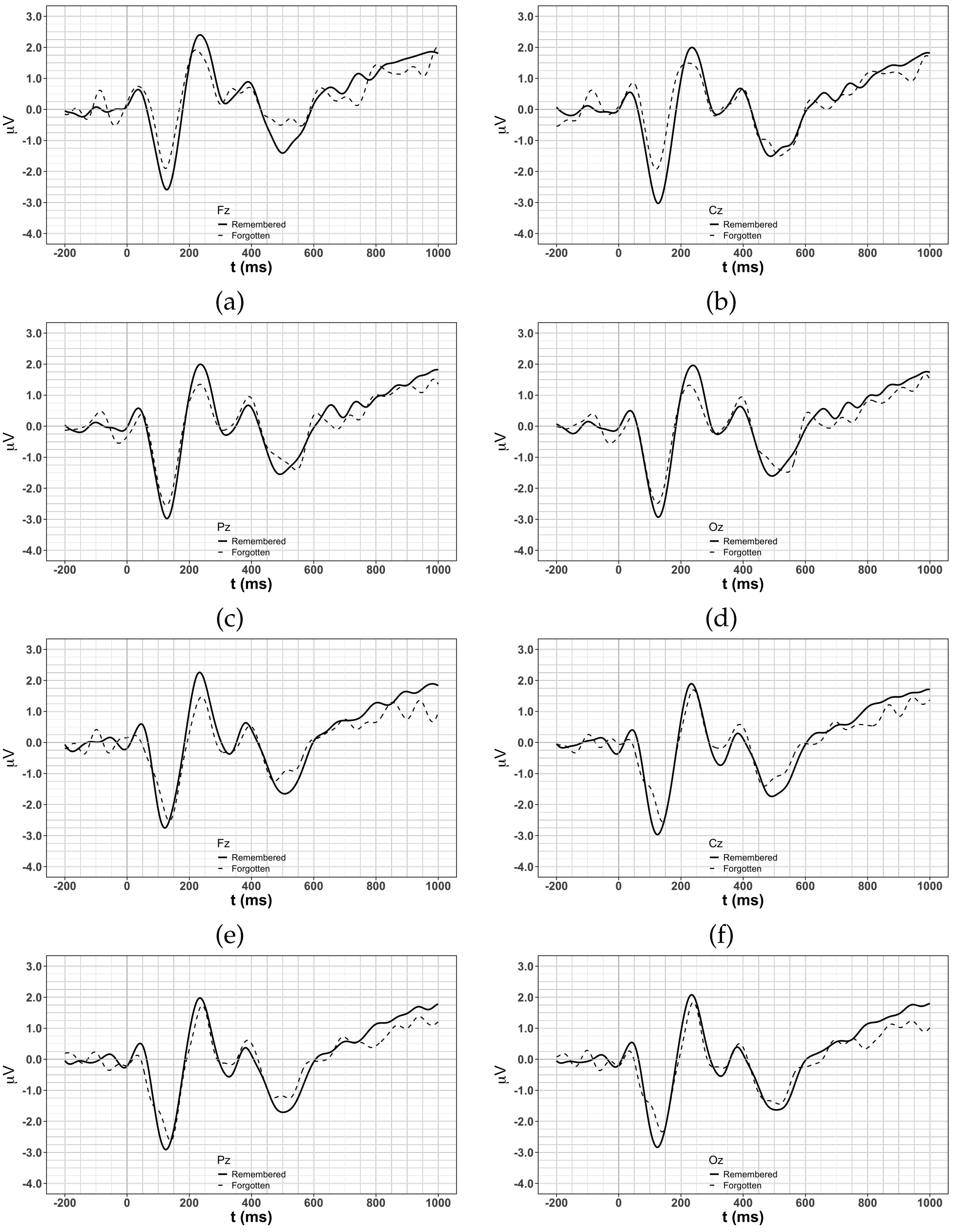

Figure S6. Group average wave forms for remembered and forgotten face/occupation associations: (a) immediate, electrode Fz; (b) immediate, electrode Cz; (c) immediate, electrode Pz; (d) immediate, electrode Oz; (e) delayed, electrode Fz; (f) delayed, electrode Cz; (g) delayed, electrode Pz; (h) delayed, electrode Oz.

Table S6. Results of the repeated measures analyses of the EEG amplitudes for the FNAM. Cells highlighted in gray indicate statistically significant results.

Test type	Retention interval	Time interval	Factor	*df*	*F*	*p*
Face/name	Immediate	200-400	Electrode (E)	3	1.54	0.298
			Recall status (R)	1	14.55	**< 0.001**
			E x R	3	1.17	0.311
		400-600	E	3	4.52	**0.005**
			R	1	1.25	0.265
			E x R	3	0.08	0.971
		600-800	E	3	14.55	**< 0.001**
			R	1	3.20	0.076
			E x R	3	0.90	0.445
	Delayed	200-400	E	3	0.34	0.798
			R	1	21.09	**<0.001**
			E x R	3	0.46	0.713
		400-600	E	3	0.10	0.960
			R	1	0.13	0.718
			E x R	3	0.62	0.606
		600-800	E	3	10.78	**< 0.001**
			R	1	0.09	0.765
			E x R	3	9.14	**< 0.001**
Face/occupation	Immediate	200-400	E	3	1.57	0.200
			R	1	3.41	0.067
			E x R	3	0.43	0.734
		400-600	E	3	0.29	0.831
			R	1	15.98	**< 0.001**
			E x R	3	1.40	0.245
		600-800	E	3	5.79	**0.001**
			R	1	0.07	0.792
			E x R	3	5.25	**0.002**
	Delayed	200-400	E	3	4.76	**0.003**
			R	1	15.76	**0.001**
			E x R	3	3.55	**0.016**
		400-600	E	3	8.55	**0.001**
			R	1	0.02	0.896
			E x R	3	6.68	**0.001**
		600-800	E	3	0.56	0.645
			R	1	2.59	0.110
			E x R	3	0.02	0.996

There were three instances in which there was a significant main effect of electrode without there being and electrode × recall status interaction. For main effect of electrode for the face/name associations at the immediate test in the 400-600 ms time interval, the mean (standard error) amplitudes were -0.11 (0.15) μV at Fz, -0.56 (0.15) μV at Cz, -0.73 (0.15) μV at Pz, and -0.71 (0.15) at Oz. For main effect of electrode for the face/name associations at the immediate test in the 600-800 ms time interval, the mean (standard error) amplitudes were 0.89 (0.15) μV at Fz, 0.42 (0.15) μV at Cz, 1.18 (0.15) μV at Pz, and 0.47 (0.15) μV at Oz. For main effect of electrode for the face/occupation associations at the immediate test in the 600-800 ms time interval, the mean (standard error) amplitudes were 0.64 (0.15) μV at Fz, 0.45 (0.15) μV at Cz, 0.97 (0.15) μV at Pz, and 0.14 (0.15) μV at Oz.

There were two instances in which there was a main effect for recall status without being involved in the interaction. For the main effect of recall status in face/name recognition at the immediate test in the 200-400 ms time interval, the mean (standard error) amplitudes were 0.83 μV (0.06) for remembered associations and 0.47 (0.06) μV for forgotten associa-

tions. For the main effect of recall status in face/occupation recognition at the immediate test in the 400-600 ms time interval, the mean (standard error) amplitudes were -1.23 (0.14) μV for remembered associations and -0.60 (0.14) μV for forgotten associations.

There were four instances in which there was a electrode × recall status interaction: in face/name recognition at the delayed test in the 600-800 ms time interval, in face/occupation recognition at the immediate test in the 600-800 ms time interval, in face/occupation recognition at the delayed test in the 200-400 ms time interval, and in face/occupation recognition at the delayed test in the 400-600 ms time window. The means for those interactions are plotted in the panels of Supplementary Figure S7.

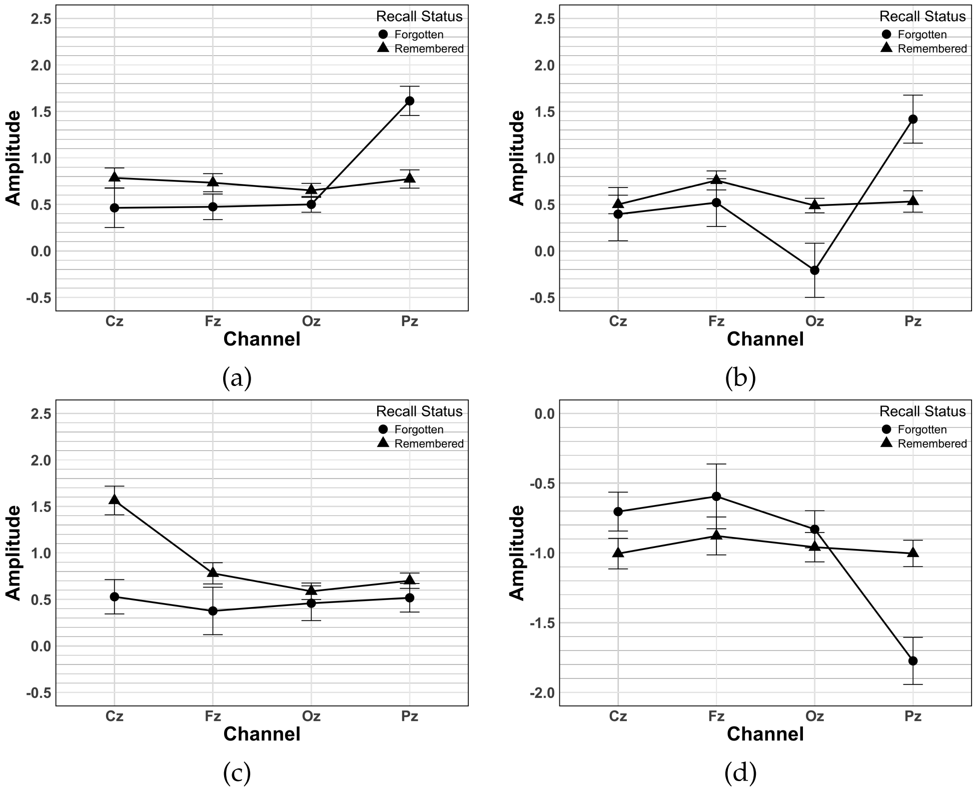

Figure S7. Group means for the significant electrode × recall status interactions in the EEG data for the FNAM: (a) face/name delayed 600-800 ms, (b) face/occupation immediate 600-800 ms, (c) face/occupation delayed 200-400 ms, (d) face/occupation delayed 400-600 ms.

4.1.4. Correlations between EEG and iron status

The complete set of correlations between the EEG variables in the conditions of the FNAM and the iron status biomarkers are presented in Supplementary Tables S7-S12.

Table S7. Correlations (1) between the EEG amplitudes and iron status biomarkers in the FNAM, for the face/name associations. Cells highlighted in gray indicate correlations that were significant after adjusting for the false discovery rate. Note: pctl = percentile.

Retention interval	Time interval	Electrode	Recall Status	Age		Hb		sFt		sFt pctl	
				r	*p*	*r*	*p*	*r*	*p*	*r*	*p*
Immediate	200-400	Cz	forgotten	0.30	0.211	-0.18	0.456	-0.05	0.844	-0.16	0.506
			remembered	0.13	0.592	0.29	0.224	0.69	**0.001**	0.50	**0.029**
		Fz	forgotten	0.24	0.334	0.25	0.311	-0.18	0.469	-0.23	0.351
			remembered	0.07	0.773	0.33	0.162	0.60	**0.007**	0.44	0.058
		Oz	forgotten	-0.32	0.184	0.01	0.976	0.55	**0.015**	0.69	**0.001**
			remembered	-0.03	0.918	0.07	0.773	-0.06	0.814	-0.08	0.754
		Pz	forgotten	0.28	0.253	-0.14	0.563	-0.07	0.768	-0.19	0.428
			remembered	0.06	0.795	-0.12	0.640	-0.18	0.474	-0.23	0.354
	400-600	Cz	forgotten	-0.10	0.678	-0.15	0.536	-0.15	0.544	-0.09	0.726
			remembered	0.22	0.363	0.18	0.472	-0.62	**0.005**	-0.69	**0.001**
		Fz	forgotten	0.32	0.181	0.20	0.415	0.19	0.449	0.03	0.909
			remembered	0.14	0.577	0.02	0.942	-0.52	**0.021**	-0.66	**0.002**
		Oz	forgotten	-0.29	0.225	-0.22	0.357	-0.59	**0.008**	-0.38	0.107
			remembered	0.19	0.432	0.45	0.051	-0.09	0.726	-0.19	0.429
		Pz	forgotten	-0.15	0.554	-0.24	0.318	-0.09	0.719	-0.01	0.960
			remembered	0.26	0.282	0.20	0.419	-0.08	0.750	-0.19	0.429
	600-800	Cz	forgotten	-0.17	0.498	-0.26	0.288	-0.08	0.751	-0.05	0.844
			remembered	-0.19	0.433	0.37	0.125	0.44	0.058	0.47	0.044
		Fz	forgotten	0.05	0.831	0.25	0.308	0.42	0.072	0.33	0.175
			remembered	0.13	0.607	0.08	0.738	0.66	**0.002**	0.55	**0.014**
		Oz	forgotten	-0.39	0.097	-0.30	0.206	-0.42	0.073	-0.25	0.310
			remembered	-0.18	0.452	0.28	0.246	0.23	0.344	0.18	0.450
		Pz	forgotten	-0.17	0.500	0.47	0.042	0.19	0.447	0.23	0.345
			remembered	-0.23	0.352	0.25	0.293	0.29	0.237	0.26	0.277
Delayed	200-400	Cz	forgotten	-0.24	0.295	-0.04	0.874	-0.02	0.930	-0.02	0.950
			remembered	0.34	0.130	0.02	0.919	0.38	0.092	0.21	0.362
		Fz	forgotten	0.16	0.478	-0.10	0.672	-0.06	0.785	-0.05	0.831
			remembered	-0.03	0.896	0.18	0.427	0.27	0.232	0.18	0.432
		Oz	forgotten	0.11	0.626	0.01	0.955	0.70	**0.000**	0.67	**0.001**
			remembered	0.05	0.824	-0.27	0.244	0.51	**0.017**	0.42	0.056
		Pz	forgotten	0.01	0.979	0.00	0.988	0.42	0.059	0.49	**0.026**
			remembered	0.24	0.306	-0.17	0.470	0.49	**0.026**	0.33	0.146
	400-600	Cz	forgotten	-0.31	0.168	0.19	0.422	0.22	0.338	0.24	0.291
			remembered	0.25	0.273	0.21	0.371	0.07	0.769	-0.05	0.820
		Fz	forgotten	0.10	0.678	-0.13	0.562	-0.31	0.171	-0.26	0.250
			remembered	-0.01	0.969	0.27	0.243	0.16	0.493	0.06	0.781
		Oz	forgotten	-0.39	0.084	-0.08	0.718	-0.11	0.643	0.03	0.904
			remembered	0.09	0.706	-0.13	0.566	-0.78	**0.000**	-0.74	**0.000**
		Pz	forgotten	-0.11	0.624	0.31	0.174	0.04	0.850	0.01	0.964
			remembered	0.29	0.205	0.04	0.850	0.31	0.171	0.22	0.339
	600-800	Cz	forgotten	-0.11	0.645	-0.03	0.915	-0.05	0.827	-0.11	0.648
			remembered	0.17	0.459	0.27	0.230	0.07	0.755	0.03	0.894
		Fz	forgotten	0.07	0.776	0.01	0.954	-0.16	0.480	-0.11	0.625
			remembered	-0.04	0.880	0.29	0.211	0.05	0.838	0.01	0.963
		Oz	forgotten	-0.42	0.059	-0.28	0.226	-0.25	0.273	-0.08	0.735
			remembered	-0.09	0.695	0.36	0.111	0.29	0.203	0.35	0.125
		Pz	forgotten	-0.13	0.588	-0.13	0.584	0.11	0.624	0.24	0.287
			remembered	0.03	0.886	0.39	0.084	0.11	0.625	0.10	0.659

Table S8. Correlations (2) between the EEG amplitudes and iron status biomarkers in the FNAM, for the face/occupation associations. Cells highlighted in gray indicate correlations that were significant after adjusting for the false discovery rate. Note: pctl = percentile

Retention interval	Time interval	Electrode	Recall Status	Age		Hb		sFt		sFt pctl	
				r	*p*	*r*	*p*	*r*	*p*	*r*	*p*
Immediate	209-400	Cz	forgotten	0.06	0.809	0.27	0.273	-0.07	0.770	-0.16	0.509
			remembered	0.36	0.134	-0.10	0.679	-0.32	0.179	-0.46	0.050
		Fz	forgotten	0.28	0.252	0.16	0.526	-0.29	0.237	-0.40	0.088
			remembered	0.01	0.963	0.26	0.292	0.24	0.323	0.26	0.280
		Oz	forgotten	-0.04	0.888	0.17	0.489	-0.37	0.121	-0.34	0.161
			remembered	-0.41	0.082	0.15	0.529	0.21	0.396	0.35	0.141
		Pz	forgotten	-0.02	0.930	0.21	0.387	-0.27	0.258	-0.27	0.263
			remembered	-0.43	0.068	0.25	0.311	0.14	0.569	0.31	0.205
	400-600	Cz	forgotten	0.07	0.772	0.33	0.162	0.01	0.956	-0.03	0.892
			remembered	0.09	0.708	-0.22	0.372	-0.10	0.698	-0.14	0.571
		Fz	forgotten	0.34	0.159	0.45	0.054	-0.01	0.984	-0.14	0.566
			remembered	-0.03	0.892	-0.31	0.200	-0.04	0.887	-0.01	0.975
		Oz	forgotten	-0.09	0.726	0.36	0.135	-0.37	0.124	-0.30	0.215
			remembered	0.19	0.438	-0.35	0.139	0.04	0.881	-0.02	0.936
		Pz	forgotten	-0.06	0.794	0.40	0.089	-0.25	0.307	-0.22	0.365
			remembered	0.15	0.535	-0.37	0.117	0.10	0.694	0.02	0.944
	600-800	Cz	forgotten	0.04	0.860	0.14	0.576	-0.26	0.287	-0.32	0.185
			remembered	-0.30	0.210	-0.20	0.412	0.25	0.301	0.36	0.127
		Fz	forgotten	0.15	0.548	0.02	0.946	0.44	0.058	0.53	**0.019**
			remembered	0.06	0.822	0.32	0.184	-0.10	0.689	-0.08	0.757
		Oz	forgotten	-0.10	0.686	0.45	0.053	0.77	**0.000**	0.76	**0.000**
			remembered	-0.21	0.400	0.01	0.956	0.16	0.521	0.29	0.236
		Pz	forgotten	0.14	0.558	0.10	0.687	0.66	**0.002**	0.60	**0.006**
			remembered	-0.13	0.589	0.14	0.575	0.20	0.403	0.31	0.198
Delayed	200-400	Cz	forgotten	0.11	0.640	-0.13	0.568	0.27	0.243	0.22	0.340
			remembered	0.09	0.692	0.48	**0.027**	-0.28	0.226	-0.29	0.208
		Fz	forgotten	0.01	0.983	0.02	0.935	0.09	0.687	0.10	0.662
			remembered	0.25	0.266	-0.01	0.953	0.11	0.644	0.00	0.995
		Oz	forgotten	0.02	0.934	0.16	0.490	-0.13	0.589	-0.10	0.679
			remembered	0.10	0.683	-0.16	0.500	0.03	0.908	-0.08	0.724
		Pz	forgotten	0.09	0.690	-0.01	0.971	0.23	0.313	0.19	0.398
			remembered	0.12	0.606	0.40	0.069	-0.07	0.753	-0.04	0.855
	400-600	Cz	forgotten	0.02	0.917	-0.28	0.222	-0.49	**0.026**	0.41	0.069
			remembered	0.33	0.149	-0.53	**0.014**	0.19	0.401	0.10	0.667
		Fz	forgotten	-0.09	0.701	0.05	0.832	0.29	0.199	0.31	0.178
			remembered	0.48	**0.026**	0.19	0.403	-0.04	0.857	-0.22	0.345
		Oz	forgotten	0.02	0.949	-0.07	0.749	0.32	0.160	0.24	0.290
			remembered	0.27	0.230	-0.22	0.342	-0.01	0.954	-0.14	0.555
		Pz	forgotten	-0.04	0.861	0.18	0.438	-0.63	**0.003**	-0.55	**0.012**
			remembered	0.28	0.223	-0.38	0.088	0.05	0.836	-0.05	0.835
	600-800	Cz	forgotten	-0.11	0.632	-0.23	0.314	0.09	0.706	0.10	0.659
			remembered	0.34	0.129	-0.08	0.726	0.04	0.860	-0.03	0.904
		Fz	forgotten	-0.12	0.619	0.10	0.675	-0.11	0.628	-0.05	0.848
			remembered	0.33	0.139	0.47	0.032	-0.15	0.521	-0.28	0.218
		Oz	forgotten	-0.20	0.390	0.08	0.741	-0.26	0.256	-0.19	0.408
			remembered	0.13	0.567	0.05	0.825	-0.10	0.674	-0.21	0.352
		Pz	forgotten	-0.12	0.602	-0.07	0.771	-0.08	0.720	-0.05	0.837
			remembered	0.07	0.755	0.00	0.988	-0.03	0.898	-0.08	0.724

Table S9. Correlations (3) between the EEG amplitudes and iron status biomarkers in the FNAM, for the face/name associations. Cells highlighted in gray indicate correlations that were significant after adjusting for the false discovery rate.

**Retention interval**	**Time Bin**	**Electrode**	**Recall Status**	**RBC** *r*	**RBC** *p*	**HCT** *r*	**HCT** *p*	**RDW** *r*	**RDW** *p*
Immediate	200-400	Cz	forgotten	-0.08	0.757	-0.09	0.714	0.27	0.256
			remembered	0.23	0.342	0.30	0.218	-0.15	0.543
		Fz	forgotten	0.22	0.356	0.20	0.422	-0.17	0.493
			remembered	0.25	0.310	0.31	0.201	-0.18	0.471
		Oz	forgotten	0.01	0.959	-0.02	0.924	-0.41	0.078
			remembered	0.22	0.371	0.12	0.627	0.24	0.318
		Pz	forgotten	-0.10	0.689	-0.09	0.705	0.28	0.249
			remembered	-0.08	0.732	-0.07	0.775	0.36	0.133
	400-600	Cz	forgotten	-0.25	0.311	-0.16	0.505	0.10	0.685
			remembered	0.09	0.718	0.05	0.853	0.36	0.136
		Fz	forgotten	0.23	0.342	0.29	0.222	-0.05	0.849
			remembered	-0.05	0.836	-0.04	0.885	0.45	0.053
		Oz	forgotten	-0.36	0.132	-0.33	0.162	0.01	0.963
			remembered	0.29	0.223	0.47	0.041	-0.11	0.649
		Pz	forgotten	-0.34	0.153	-0.26	0.287	0.04	0.864
			remembered	0.02	0.953	0.17	0.490	-0.06	0.804
	600-800	Cz	forgotten	-0.35	0.148	-0.23	0.341	0.20	0.415
			remembered	0.26	0.278	0.28	0.245	-0.20	0.421
		Fz	forgotten	0.31	0.204	0.37	0.125	-0.12	0.633
			remembered	0.11	0.652	0.14	0.577	-0.27	0.258
		Oz	forgotten	-0.47	0.041	-0.40	0.090	0.06	0.824
			remembered	0.23	0.352	0.32	0.179	-0.08	0.738
		Pz	forgotten	0.48	0.038	0.42	0.074	-0.14	0.573
			remembered	0.16	0.514	0.24	0.314	-0.13	0.610
Delayed	200-400	Cz	forgotten	0.03	0.897	0.02	0.922	0.09	0.715
			remembered	-0.02	0.920	0.08	0.730	-0.08	0.748
		Fz	forgotten	-0.22	0.347	-0.15	0.518	0.09	0.710
			remembered	0.05	0.818	0.19	0.413	-0.06	0.814
		Oz	forgotten	0.10	0.676	-0.05	0.841	-0.19	0.413
			remembered	-0.32	0.153	-0.19	0.408	-0.12	0.617
		Pz	forgotten	0.06	0.786	0.05	0.839	0.08	0.746
			remembered	-0.18	0.430	-0.07	0.768	-0.03	0.892
	400-600	Cz	forgotten	0.22	0.346	0.26	0.255	-0.08	0.741
			remembered	0.06	0.792	0.20	0.389	-0.02	0.936
		Fz	forgotten	-0.22	0.334	-0.19	0.415	0.21	0.372
			remembered	0.04	0.870	0.23	0.314	-0.05	0.823
		Oz	forgotten	-0.26	0.260	-0.19	0.406	0.05	0.819
			remembered	-0.19	0.409	-0.19	0.408	0.29	0.201
		Pz	forgotten	0.34	0.127	0.37	0.099	-0.14	0.534
			remembered	0.04	0.873	0.07	0.760	-0.11	0.641
	600-800	Cz	forgotten	0.01	0.966	0.08	0.728	0.15	0.523
			remembered	0.26	0.254	0.36	0.106	-0.15	0.504
		Fz	forgotten	-0.11	0.624	-0.07	0.753	0.03	0.913
			remembered	0.17	0.461	0.31	0.169	-0.14	0.541
		Oz	forgotten	-0.34	0.129	-0.30	0.192	0.09	0.711
			remembered	0.39	0.078	0.37	0.102	-0.27	0.236
		Pz	forgotten	-0.11	0.628	-0.17	0.458	-0.03	0.910
			remembered	0.43	0.054	0.44	0.045	-0.11	0.644

Table S10. Correlations (4) between the EEG amplitudes and iron status biomarkers in the FNAM, for the face/occupation associations. Cells highlighted in gray indicate correlations that were significant after adjusting for the false discovery rate.

Retention interval	Time Bin	Electrode	Recall Status	RBC		HCT		RDW	
				r	*p*	*r*	*p*	*r*	*p*
Immediate	200-400	Cz	forgotten	0.14	0.556	0.30	0.206	0.17	0.498
			remembered	-0.15	0.544	-0.01	0.956	0.26	0.290
		Fz	forgotten	0.14	0.567	0.21	0.394	0.12	0.624
			remembered	0.23	0.345	0.09	0.712	-0.29	0.236
		Oz	forgotten	0.05	0.848	0.17	0.485	0.16	0.507
			remembered	0.27	0.273	0.08	0.734	-0.21	0.382
		Pz	forgotten	0.12	0.621	0.24	0.320	0.24	0.323
			remembered	0.31	0.201	0.18	0.470	-0.19	0.425
	400-600	Cz	forgotten	0.22	0.375	0.29	0.236	-0.02	0.929
			remembered	-0.21	0.378	-0.05	0.855	-0.01	0.970
		Fz	forgotten	0.35	0.141	0.46	0.049	-0.19	0.449
			remembered	-0.30	0.220	-0.12	0.624	0.02	0.947
		Oz	forgotten	0.19	0.442	0.27	0.256	0.06	0.800
			remembered	-0.41	0.080	-0.21	0.398	0.03	0.903
		Pz	forgotten	0.26	0.290	0.34	0.152	0.13	0.608
			remembered	-0.43	0.069	-0.18	0.451	-0.13	0.590
	600-800	Cz	forgotten	-0.03	0.910	0.09	0.712	0.22	0.378
			remembered	-0.31	0.193	-0.26	0.281	-0.02	0.929
		Fz	forgotten	-0.05	0.844	0.03	0.920	0.20	0.406
			remembered	0.12	0.622	0.09	0.707	-0.22	0.360
		Oz	forgotten	0.33	0.163	0.46	0.050	-0.45	0.054
			remembered	-0.06	0.825	-0.17	0.478	-0.09	0.712
		Pz	forgotten	0.13	0.601	0.18	0.459	-0.42	0.073
			remembered	0.07	0.791	-0.03	0.893	0.02	0.937
Delayed	200-400	Cz	forgotten	0.01	0.958	-0.06	0.794	0.09	0.690
			remembered	0.38	0.087	0.39	0.083	-0.04	0.860
		Fz	forgotten	0.17	0.453	0.16	0.495	0.04	0.882
			remembered	-0.17	0.452	0.07	0.749	-0.22	0.336
		Oz	forgotten	0.23	0.308	0.14	0.554	0.07	0.753
			remembered	-0.25	0.272	-0.13	0.567	-0.05	0.841
		Pz	forgotten	0.08	0.725	0.02	0.947	0.12	0.599
			remembered	0.44	0.048	0.33	0.149	-0.10	0.655
	400-600	Cz	forgotten	-0.18	0.425	-0.11	0.652	0.02	0.940
			remembered	-0.41	0.065	-0.42	0.055	0.00	0.994
		Fz	forgotten	-0.04	0.869	0.19	0.401	-0.35	0.118
			remembered	0.01	0.951	0.22	0.346	-0.14	0.559
		Oz	forgotten	0.05	0.818	0.07	0.753	0.12	0.602
			remembered	-0.17	0.473	-0.18	0.448	0.09	0.700
		Pz	forgotten	0.07	0.784	0.01	0.980	0.01	0.955
			remembered	-0.31	0.177	-0.35	0.120	0.10	0.676
	600-800	Cz	forgotten	-0.07	0.754	-0.08	0.741	0.16	0.481
			remembered	-0.14	0.552	-0.12	0.600	-0.25	0.274
		Fz	forgotten	0.09	0.702	0.22	0.350	-0.21	0.371
			remembered	0.16	0.496	0.43	0.050	-0.21	0.356
		Oz	forgotten	0.12	0.592	0.15	0.510	0.13	0.574
			remembered	-0.16	0.497	-0.03	0.886	-0.05	0.847
		Pz	forgotten	0.07	0.772	0.07	0.758	0.18	0.444
			remembered	-0.22	0.349	-0.16	0.492	-0.11	0.633

Table S11. Correlations (5) between the EEG amplitudes and iron status biomarkers in the FNAM, for the face/name associations. Cells highlighted in gray indicate correlations that were significant after adjusting for the false discovery rate.

**Retention interval**	**Time Bin**	**Electrode**	**Recall Status**	**MCV**		**MCH**		**MCHC**	
				r	*p*	*r*	*p*	*r*	*p*
Immediate	200-400	Cz	forgotten	0.00	0.996	-0.14	0.565	-0.28	0.242
			remembered	0.03	0.903	0.08	0.736	0.11	0.643
		Fz	forgotten	-0.11	0.653	-0.01	0.960	0.19	0.442
			remembered	0.01	0.961	0.11	0.661	0.20	0.410
		Oz	forgotten	-0.08	0.744	-0.04	0.888	0.09	0.725
			remembered	-0.17	0.492	-0.20	0.414	-0.09	0.731
		Pz	forgotten	0.03	0.9	-0.05	0.833	-0.16	0.503
			remembered	0.06	0.823	-0.03	0.917	-0.14	0.557
	400-600	Cz	forgotten	0.17	0.476	0.14	0.555	-0.02	0.941
			remembered	-0.08	0.75	0.10	0.686	0.35	0.137
		Fz	forgotten	-0.01	0.982	-0.09	0.720	-0.16	0.512
			remembered	0.05	0.838	0.10	0.688	0.11	0.648
		Oz	forgotten	0.12	0.623	0.20	0.417	0.18	0.461
			remembered	0.15	0.553	0.19	0.449	0.11	0.643
		Pz	forgotten	0.21	0.384	0.17	0.479	-0.04	0.863
			remembered	0.19	0.448	0.23	0.341	0.14	0.581
	600-800	Cz	forgotten	0.25	0.303	0.15	0.534	-0.16	0.520
			remembered	-0.06	0.822	0.12	0.641	0.34	0.154
		Fz	forgotten	-0.02	0.946	-0.11	0.643	-0.19	0.435
			remembered	-0.02	0.952	-0.06	0.811	-0.09	0.720
		Oz	forgotten	0.24	0.329	0.28	0.247	0.11	0.654
			remembered	0.06	0.812	0.05	0.831	0.01	0.975
		Pz	forgotten	-0.22	0.363	-0.06	0.795	0.29	0.229
			remembered	0.07	0.792	0.11	0.642	0.12	0.623
Delayed	200-400	Cz	forgotten	-0.02	0.924	-0.10	0.680	-0.17	0.456
			remembered	0.16	0.478	0.09	0.697	-0.12	0.597
		Fz	forgotten	0.14	0.556	0.17	0.451	0.11	0.641
			remembered	0.17	0.466	0.18	0.445	0.05	0.826
		Oz	forgotten	-0.25	0.27	-0.15	0.513	0.18	0.448
			remembered	0.27	0.241	0.12	0.617	-0.27	0.233
		Pz	forgotten	-0.03	0.903	-0.08	0.733	-0.13	0.591
			remembered	0.22	0.348	0.06	0.810	-0.30	0.193
	400-600	Cz	forgotten	0.00	0.995	-0.06	0.814	-0.12	0.609
			remembered	0.15	0.522	0.17	0.460	0.09	0.710
		Fz	forgotten	0.09	0.705	0.12	0.615	0.08	0.724
			remembered	0.23	0.313	0.29	0.198	0.18	0.440
		Oz	forgotten	0.15	0.523	0.23	0.309	0.21	0.366
			remembered	0.05	0.837	0.08	0.729	0.06	0.792
		Pz	forgotten	-0.04	0.853	-0.06	0.801	-0.04	0.853
			remembered	0.03	0.916	-0.01	0.972	-0.05	0.817
	600-800	Cz	forgotten	0.09	0.708	-0.05	0.818	-0.27	0.231
			remembered	0.07	0.769	0.02	0.944	-0.10	0.677
		Fz	forgotten	0.08	0.737	0.18	0.426	0.23	0.319
			remembered	0.14	0.533	0.16	0.489	0.05	0.818
		Oz	forgotten	0.15	0.53	0.10	0.679	-0.08	0.728
			remembered	-0.14	0.544	-0.06	0.788	0.13	0.578
		Pz	forgotten	-0.05	0.833	-0.01	0.968	0.07	0.761
			remembered	-0.09	0.705	-0.07	0.759	0.01	0.953

Table S12. Correlations (6) between the EEG amplitudes and iron status biomarkers in the FNAM, for the face/occupation associations. Cells highlighted in gray indicate correlations that were significant after adjusting for the false discovery rate.

Retention interval	Time Bin	Electrode	Recall Status	MCV *r*	MCV *p*	MCH *r*	MCH *p*	MCHC *r*	MCHC *p*
Immediate	200-400	Cz	forgotten	0.17	0.476	0.16	0.528	-0.01	0.976
			remembered	0.20	0.404	0.06	0.795	-0.25	0.302
		Fz	forgotten	0.05	0.846	0.00	0.996	-0.08	0.738
			remembered	-0.25	0.313	0.02	0.943	0.49	0.034
		Oz	forgotten	0.16	0.505	0.18	0.468	0.05	0.842
			remembered	-0.31	0.195	-0.17	0.479	0.23	0.344
		Pz	forgotten	0.14	0.565	0.13	0.602	-0.01	0.977
			remembered	-0.26	0.287	-0.11	0.655	0.26	0.282
	400-600	Cz	forgotten	0.03	0.889	0.14	0.564	0.22	0.360
			remembered	0.28	0.253	0.02	0.925	-0.48	0.039
		Fz	forgotten	0.02	0.922	0.08	0.736	0.13	0.596
			remembered	0.32	0.188	0.02	0.923	-0.54	**0.016**
		Oz	forgotten	0.07	0.78	0.22	0.366	0.31	0.194
			remembered	0.39	0.096	0.14	0.583	-0.48	0.039
		Pz	forgotten	0.05	0.843	0.18	0.461	0.27	0.260
			remembered	0.44	0.062	0.12	0.612	-0.57	**0.011**
	600-800	Cz	forgotten	0.16	0.504	0.22	0.362	0.15	0.554
			remembered	0.19	0.443	0.22	0.375	0.08	0.747
		Fz	forgotten	0.11	0.653	0.09	0.723	-0.04	0.884
			remembered	-0.08	0.735	0.25	0.303	0.65	**0.003**
		Oz	forgotten	0.04	0.878	0.12	0.635	0.17	0.484
			remembered	-0.14	0.579	0.10	0.686	0.46	0.050
		Pz	forgotten	0.01	0.957	-0.06	0.810	-0.13	0.584
			remembered	-0.15	0.552	0.10	0.695	0.46	0.049
Delayed	200-400	Cz	forgotten	-0.06	0.793	-0.15	0.505	-0.20	0.394
			remembered	-0.12	0.594	0.08	0.746	0.40	0.075
		Fz	forgotten	-0.04	0.848	-0.19	0.405	-0.32	0.155
			remembered	0.38	0.094	0.24	0.303	-0.21	0.354
		Oz	forgotten	-0.16	0.481	-0.09	0.701	0.11	0.639
			remembered	0.21	0.357	0.15	0.529	-0.11	0.637
		Pz	forgotten	-0.07	0.76	-0.09	0.706	-0.04	0.849
			remembered	-0.27	0.23	-0.09	0.688	0.34	0.137
	400-600	Cz	forgotten	0.19	0.408	-0.08	0.740	-0.50	**0.021**
			remembered	0.11	0.649	-0.12	0.609	-0.45	0.043
		Fz	forgotten	0.33	0.147	0.15	0.525	-0.33	0.150
			remembered	0.25	0.269	0.23	0.319	0.01	0.982
		Oz	forgotten	0.04	0.861	-0.15	0.522	-0.38	0.092
			remembered	0.02	0.944	-0.08	0.730	-0.20	0.393
		Pz	forgotten	-0.11	0.639	0.14	0.566	0.47	0.036
			remembered	0.03	0.914	-0.09	0.698	-0.24	0.304
	600-800	Cz	forgotten	0.05	0.82	-0.16	0.484	-0.44	0.046
			remembered	0.08	0.737	0.11	0.640	0.06	0.790
		Fz	forgotten	0.16	0.483	0.04	0.874	-0.25	0.279
			remembered	0.31	0.169	0.41	0.063	0.26	0.259
		Oz	forgotten	0.03	0.913	-0.04	0.852	-0.16	0.496
			remembered	0.21	0.362	0.31	0.179	0.23	0.327
		Pz	forgotten	0.02	0.935	-0.15	0.522	-0.36	0.114
			remembered	0.16	0.483	0.34	0.127	0.38	0.086

4.2. PST

4.2.1. Behavioral data

Descriptive statistics for the behavioral data from the PST are presented in Supplementary Table S13. Accuracy data (choosing A vs. avoiding B) were analyzed using a one-way repeated measures mixed models ANOVA, with choice type (choose A, avoid B) manipulated within participants and participants as the random factor, and the results are presented in Supplementary Table S14. There was no significant effect of choice type on accuracy, indicating that participants were equally accurate in correctly choosing A (M = .78, SE = .03) vs. avoiding B (M = .79, SE = .03). This aligns with previous literature, suggesting that healthy individuals do not show a difference in either approach or avoidance learning [5,6].

Table S13. Descriptive statistics for the behavioral data from the PST.

Factor	DV	Level	*M*	*SE*	*min*	*max*
Choice type	P(C)	Choose A	0.78	0.03	0.36	1
		Avoid B	0.79	0.03	0.52	1
Conflict Level	RT (ms)	Low	1027	50	647	1521
		High	1311	67	752	1962

Table S14. Results of the repeated measures ANOVA on the behavioral variables from the PST. Cells highlighted in gray indicate statistically significant effects.

Factor	DV	*F*	*p*
Choice Type	Accuracy, P(C)	0.16	0.691
Conflict Level	RT, ms	23.07	< 0.001

A one-way repeated measures ANOVA was performed on correct RTs with conflict level (low, high) manipulated within participants and participants as the random factor. This analysis (see Supplementary Table S14) revealed a main effect for level of conflict, with participants responding more quickly in the low conflict condition (M = 1027, SE = 50) relative to the high conflict condition (M = 1311, SE = 67). These results agree with previous literature, suggesting a conflict-induced slowing in healthy adults [6].

Pearson correlations were calculated involving response accuracy for the two choices and the blood iron biomarkers, and the results are presented in Tables S15 and **??**. Levels of sFt were positively correlated with accuracy of choosing A. Accuracy of correctly avoiding B was positively correlated with sFt and sFt percentile. The results with sFt are consistent with the idea that higher levels of iron should be associated with better dopaminergic system integrity, with the latter being associated with higher levels of performance [5,21].

Pearson correlations were calculated for correct RTs for the two conflict levels (low vs. high) and the iron biomarkers and age (Supplementary Tables S15 and **??**). RTs in the high conflict condition were negatively correlated with sFt, sFt percentile and with MCH. According to Frank et al. [6], participants with higher DA levels showed shorter RTs. Given the evidence in animal models of relationship between iron levels and central, this suggests that even in women who are not iron deficient there still may be a positive relationship between iron status and DA. In addition the relationship with MCH again points to the potential involvement of oxygen transport in non-anemic women.

Table S15. Correlations of the behavioral variables in the PST with the iron status biomarkers (1). Cells highlighted in gray indicate correlations that were significant after correcting for the false discovery rate. Note: pctl = percentile.

	Hb		sFt		sFt pctl		Age	
Variable	*r*	*p*	*r*	*p*	*r*	*p*	*r*	*p*
Choose A, P(C)	0.40	0.047	0.46	**0.022**	0.34	0.101	0.10	0.634
Avoid B, P(C)	-0.11	0.604	0.50	**0.012**	0.41	0.040	0.14	0.502
Low Conflict, RT, ms	0.13	0.547	-0.27	0.195	-0.30	0.148	0.18	0.394
High Conflict, RT, ms	-0.22	0.290	-0.42	**0.034**	-0.47	**0.019**	0.25	0.226

4.2.2. EEG and correlations with iron status

Supplementary Figure S8 presents the group averages for the response-locked (Supplementary Figure S8a) and feedback-locked (Supplementary Figure S8b) EEG features. Supplementary Table S17 presents the correlations involving all of the features and the iron status biomarkers. Each of the features was significantly related to iron status, such the higher iron status was related to larger (positive or negative) amplitudes. There were no significant correlations with any of the biomarkers associated with oxygen transport.

Finally, age was significantly related with both of the FRNs, such that higher age was associated with lower amplitudes.

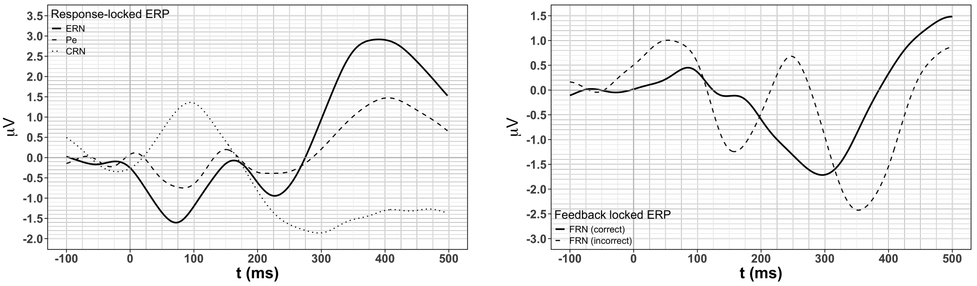

(a) (b)

Figure S8. Group average EEG data from the PST: (a) response-locked features, (b) feedback locked features.

Table S17. Correlations involving the EEG features from the PST with the iron status biomarkers. Cells highlighted in gray indicate correlations that were significant after correcting for the false discovery rate. Note: FRN (c) = FRN correct trials, FRN (i) = FRN incorrect trials

	ERN		Pe		CRN		FRN (c)		FRN (i)	
	r	*p*	*r*	*p*	*r*	*p*	*r*	*p*	*r*	*p*
Age	0.29	0.171	-0.27	0.209	0.36	0.082	0.45	**0.029**	0.44	**0.033**
Hb	-0.22	0.309	0.15	0.472	-0.13	0.540	-0.16	0.470	-0.11	0.598
sFt	-0.81	**< 0.001**	0.69	**< 0.001**	-0.84	**< 0.001**	-0.75	**< 0.001**	-0.76	**< 0.001**
sFt percentile	-0.88	**< 0.001**	0.73	**< 0.001**	-0.94	**< 0.001**	-0.91	**< 0.001**	-0.92	**< 0.001**
RBC	-0.13	0.537	0.23	0.284	-0.02	0.922	0.02	0.940	0.01	0.960
Hct	-0.11	0.618	0.06	0.780	-0.05	0.829	-0.04	0.863	-0.01	0.950
RDW	0.35	0.092	-0.33	0.113	0.25	0.236	0.25	0.236	0.27	0.198
MCV	0.05	0.823	-0.28	0.194	-0.05	0.815	-0.09	0.661	-0.05	0.830
MCH	-0.13	0.560	-0.10	0.633	-0.17	0.427	-0.25	0.242	-0.18	0.404
MCHC	-0.35	0.092	0.30	0.153	-0.26	0.216	-0.35	0.097	-0.29	0.164

4.3. RBCL

4.3.1. Behavioral data

Descriptive statistics for each of the behavioral variables from the RBCL are presented in Supplementary Table S18. Each of the variables were analyzed using a one-way repeated measures mixed models ANOVA, with block (first 200 vs. second 200 trials) manipulated within participants and with participants as the random factor, and the results are presented in Supplementary Table S19. The analysis of the accuracy variable revealed a significant main effect of block, showing that participants performed more accurately in block 2 (M = .49, SE = .02) than in block 1 (M = .44, SE = .02), indicating a modest learning effect. Note that chance level of accuracy in this task is 0.25. This was the only variable for which there was a significant main effect for block.

Table S18. Descriptive statistics for the behavioral data from the RBCL.

	Block 1				Block 2			
Variable	M	SE	min	max	M	SE	min	max
Overall accuracy, P(C)	0.44	0.02	0.30	0.71	0.49	0.02	0.29	0.71
Marginal hit rate, orientation	0.54	0.02	0.34	0.79	0.56	0.03	0.04	0.90
Marginal false alarm rate, orientation	0.36	0.03	0.09	0.67	0.27	0.03	0.10	0.61
Marginal d', orientation	0.53	0.10	-0.23	1.50	0.81	0.10	-0.69	1.62
Marginal c, orientation	0.14	0.07	-0.55	0.83	0.25	0.07	-0.47	1.43
Marginal hit rate, spatial frequency	0.75	0.03	0.49	0.98	0.82	0.02	0.63	0.96
Marginal false alarm rate, spatial frequency	0.34	0.02	0.11	0.53	0.30	0.02	0.09	0.58
Marginal d', spatial frequency	1.23	0.13	0.01	2.89	1.48	0.13	0.35	2.46
Marginal c, spatial frequency	-0.17	0.05	-0.75	0.30	-0.17	0.05	-0.73	0.59
RT, correct responses, ms	1059	27	837	1346	1024	27	798	1337

Table S19. Results of the repeated measures ANOVAs on the behavioral data from the RBCL.

Variable	*F*	*p*
Overall accuracy, P(C)	4.45	0.045
Marginal d', orientation	4.24	0.050
Marginal c, orientation	1.32	0.261
Marginal d', spatial frequency	2.95	0.098
Marginal c, spatial frequency	0.01	0.918
RT, correct responses, ms	2.29	0.143

Pearson correlations were run on accuracy and all of the other behavioral variables with the iron biomarkers and age, and the results are presented in Supplementary Table S20. No significant correlations involving accuracy were observed in block 1. However, there were significant positive correlations involving accuracy and sFt and sFt percentile in block 2, consistent with previous work with women of reproductive age [29,30].

Table S20. Correlations involving the behavioral data from the RBCL with the iron status biomarkers (1). Cells highlighted in gray indicate correlations that were statistically significant after correcting for the false discovery rate. Note: pctl = percentile.

		Hb		sFt		sFt pctl		Age	
Variable	Block	*r*	*p*	*r*	*p*	*r*	*p*	*r*	*p*
Overall accuracy, P(C)	1	0.06	0.758	-0.14	0.486	-0.15	0.826	-0.23	0.267
	2	-0.21	0.306	0.51	**0.008**	0.52	**0.006**	-0.10	0.633
Hit rate (%), orientation	1	0.18	0.378	-0.18	0.380	-0.16	0.444	0.10	0.626
	2	0.07	0.735	0.50	**0.010**	0.42	0.035	0.04	0.843
False alarm rate (%), orientation	1	-0.01	0.978	0.05	0.798	0.00	0.993	0.25	0.227
	2	0.22	0.291	0.11	0.593	0.03	0.100	0.09	0.669
Marginal d', orientation	1	0.14	0.499	-0.20	0.323	-0.13	0.539	-0.19	0.363
	2	-0.13	0.534	0.37	0.062	0.37	0.060	-0.06	0.764
Marginal c, orientation	1	-0.08	0.687	0.04	0.859	0.07	0.742	-0.23	0.266
	2	-0.11	0.577	-0.42	0.034	-0.32	0.116	-0.06	0.780
Hit rate (%), spatial frequency	1	-0.12	0.565	-0.03	0.887	0.07	0.752	-0.25	0.222
	2	0.04	0.843	0.56	**0.003**	0.59	**0.002**	-0.18	0.391
False alarm rate (%), spatial frequency	1	0.01	0.974	0.17	0.407	0.08	0.709	0.16	0.441
	2	0.28	0.159	0.13	0.526	0.01	0.967	0.25	0.223
Marginal d', spatial frequency	1	-0.13	0.521	-0.17	0.395	-0.07	0.794	-0.24	0.228
	2	-0.31	0.123	0.38	0.054	0.46	**0.018**	-0.27	0.175
Marginal c, spatial frequency	1	0.21	0.311	0.01	0.969	-0.04	0.851	0.18	0.379
	2	-0.08	0.702	-0.58	**0.002**	-0.51	**0.007**	-0.04	0.846
RT, correct responses, ms	1	-0.03	0.870	0.20	0.336	0.08	0.700	0.35	0.077
	2	0.06	0.782	-0.15	0.462	-0.25	0.226	0.18	0.385

Pearson correlations of overall accuracy and the marginal signal detection measures, including marginal hit rate, marginal false alarm rate, marginal d', and marginal c, with the iron status biomarkers and age were calculated for both blocks. Overall accuracy was positively correlated with sFt and sFt percentile in block 2, indicating that higher values of these iron biomarkers were associated with higher asymptotic levels of performance, consistent with work with women of reproductive age [29,30]. The marginal hit rate for orientation was also positively correlated with both sFt and sFt percentile in block 2, also consistent with results with women of reproductive age [29,30].

No significant correlations were found between the iron markers and age with marginal false alarm rate or marginal d' for orientation in either block. There was, however, a negative correlation between marginal c for orientation and sFt in block 2, showing that higher levels of sFt were associated with more liberal responding in the final portion of the task. While this last result is consistent with associations involving response criterion noted earlier, and with work with women of reproductive age [31], this suggests that iron levels were affecting only response bias and not sensitivity for orientation, an outcome which is inconsistent with previous work with this task [30].

The marginal hit rate for spatial frequency was positively correlated with both sFt and sFt percentile in block 2, indicating that higher hit rates for spatial frequency were associated with higher values of sFt and sFt percentile, which aligns with previous literature

suggesting that higher levels of sFt are related to improvements in performance on this task [30]. No significant correlations were found between the iron markers with false alarms rate for spatial frequency in either block, which was unexpected due to the previous findings that lower sFt values predicted greater reductions in false alarm rates [32]. Marginal d' for spatial frequency was positively correlated with sFt percentile in block 2, indicating that higher levels of sFt were associated with higher asymptotic discriminability for spatial frequency, a result that is consistent with prior results with women of reproductive age [29,30]. Marginal c for spatial frequency was negatively correlated with both sFt and sFt percentile in block 2. This result is consistent with the results for orientation. However, sFt levels were associated with differences in both sensitivity and response bias, consistent with previous work with women of reproductive age [30].

Reaction times for correct responses were analyzed using a one-way repeated measures mixed models ANOVA, with block (first 200 vs. second 200 trials) manipulated within participants and participants as the random factor. Results did not indicate any significant main effects of RT for block. This is inconsistent with previous results with women of reproductive age, in which increasing practice with the task led to reductions in RT [30]. Since there was a main effect of accuracy but not RT, this may suggest that participants are prioritizing accuracy over speed, taking more time to respond to ensure correct answers, even if it results in slower response times.

Pearson correlations involving RT were conducted. No significant correlations were found for block 1. However, RT was positively correlated with RDW in block 2, indicating that longer RTs were associated with higher values of RDW. Since higher values of RDW are associated with poorer oxygen transport capability, this result is consistent with expectations.

4.3.2. Correlations between EEG and iron status

The group average waveforms for the stimulus- and feedback locked EEG data are presented in Supplementary Figure S9. The complete set of correlations of these features with the iron status biomarkers are presented in Supplementary Table **??**. Note that these data are all collapsed across blocks in order to obtain the most stable estimates for correct and incorrect trials. Age was negatively correlated with the amplitudes of the P300 (both correct and incorrect) and the late positive slow wave (both correct and incorrect), indicating lower amplitudes with increasing age. The amplitudes of both of these features, for both correct and incorrect trials, were positively related to both sFt and sFt percentile, as was fractional area latency. This is consistent with previous work with women of reproductive age who were iron deficient but not anemic [32,33] in a sample of women who were not iron deficient. The amplitude of the late positive slow wave, for both correct and incorrect trials, was negatively related to RDW, and the fractional area latency for correct trials was positively related to MCH. Both of these findings that improved iron status with respect to oxygen transport capability was related to better brain function, with this being true in a sample of women who were not anemic.

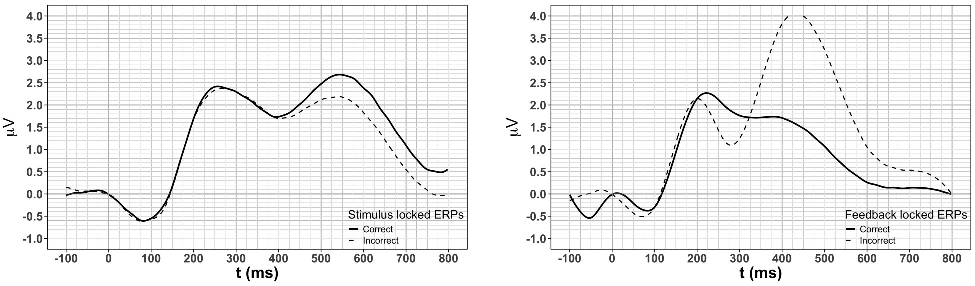

(a) (b)

Figure S9. Group average EEG data from the RBCL: (a) stimulus-locked features, (b) feedback locked features.

4.4. VSWM

4.4.1. Behavioral data

Descriptive statistics for all of the behavioral variables are presented in Supplementary Table S22. Accuracy and RT data were analyzed using 2 (number of targets: 3, 5) × 2 (number of distractors: 0, 2) × 2 (target: absent, present) repeated measures mixed models ANOVAs, with all three factors manipulated within participants and participants as the random factor. The signal detection measures (d', c) and the working memory capacity measures (K, K') were analyzed using 2 (number of targets: 3, 5) × 2 (number of distractors: 0, 2) repeated measures mixed models ANOVAs, with both factors manipulated within participants and participants as the random factor. The results of these analyses are presented in Supplementary Table S23.

Table S22. Descriptive statistics for the behavioral variables from the VSWM. Note: pres/abs = present/absent.

	Targets	Distractors	Target pres/abs	M	SE	min	max
Accuracy P(C)	3	0	A	0.970	0.03	0.83	1.00
			P	0.970	0.03	0.83	1.00
		2	A	0.960	0.03	0.83	1.00
			P	0.960	0.03	0.67	1.00
	5	0	A	0.910	0.03	0.67	1.00
			P	0.800	0.03	0.00	1.00
		2	A	0.880	0.03	0.58	1.00
			P	0.800	0.03	0.00	1.00
RT, ms	3	0	A	933	40	562	1238
			P	918	40	540	1324
		2	A	941	40	581	1420
			P	915	40	606	1408
	5	0	A	989	40	614	1358
			P	984	40	563	1351
		2	A	1051	40	591	1591
			P	992	40	704	1300
Hit rate	3	0		0.96	0.01	0.83	0.99
		2		0.95	0.01	0.67	0.99
	5	0		0.81	0.04	0.01	0.99
		2		0.80	0.05	0.01	0.99
False alarm rate	3	0		0.04	0.01	0.01	0.17
		2		0.04	0.01	0.01	0.17
	5	0		0.10	0.02	0.01	0.33
		2		0.12	0.02	0.01	0.42
d'	3	0		4.02	0.15	2.35	4.65
		2		3.94	0.19	1.40	4.65
	5	0		2.65	0.26	0.00	4.65
		2		2.57	0.26	0.00	4.65
c	3	0		0.00	0.05	-0.47	0.47
		2		0.00	0.05	-0.47	0.47
	5	0		0.27	0.11	-0.47	2.33
		2		0.13	0.14	-0.95	2.33
K	3	0		2.88	0.03	2.45	2.97
		2		2.84	0.05	1.80	2.97
	5	0		3.88	0.23	0.00	4.95
		2		3.88	0.27	0.00	4.95
K'	3	0		0.40	0.03	0.38	0.43
		2		0.39	0.05	0.34	0.43
	5	0		0.45	0.02	0.00	0.54
		2		0.43	0.03	0.00	0.54

Table S23. Repeated measures ANOVAs on the behavioral variables from the VSWM. Cells highlighted in gray indicate significant effects.

Variable	Factor	*F*	*p*
Accuracy P(C)	Targets (T)	32.56	**< 0.001**
	Distractors (D)	0.32	0.578
	Present/absent (P)	6.48	**0.018**
	T x D	0.03	0.875
	T x P	5.11	**0.033**
	D x P	0.06	0.809
	T x D x P	0.11	0.744
RT, correct responses, ms	T	27.19	**< 0.001**
	D	1.58	0.221
	P	3.20	0.086
	T x D	1.19	0.286
	T x P	0.17	0.682
	D x P	1.20	0.285
	T x D x P	0.53	0.474
K	T	25.22	**< 0.001**
	D	0.03	0.860
	T x D	0.01	0.920
K'	T	5.86	**0.023**
	D	0.46	0.505
	T x D	0.29	0.598
d'	T	46.73	**< 0.001**
	D	0.44	0.514
	T x D	0.00	0.956
c	T	3.69	0.066
	D	0.71	0.406
	T x D	0.51	0.480

Results for accuracy showed a main effect of number of targets, indicating that participants performed more accurately when 3 targets were present (M = .97, SE = .01) than when 5 targets were present (M = .85, SE = .01). This shows that accuracy was higher when there were fewer items to be remembered, which is to be expected [34]. Results also indicated that accuracy was higher when the target was absent (M = .93, SE = .01) relative to when it was present (M = .88, SE = .01), suggesting that participants were more accurate at rejecting a probe at a location that was not occupied by a target than they were at confirming a probe at a location that was occupied by a target, which also aligns with previous research [34]. There was a significant interaction between the number of targets presented and whether a target was present or absent, wherein participants were more accurate when the target was absent but that this was true only when five targets were presented (Supplementary Figure S10). This could indicate that the five target condition was demanding to the point of there being no advantage for target absent over target present trials.

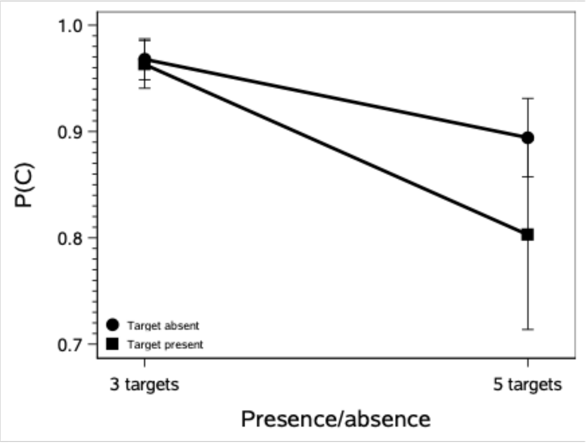

Figure S10. Interaction of number of targets and target present/absent in the accuracy data, VSWM.

Results of the analyses of the RT data revealed a main effect of number of targets, indicating that participants responded faster when three targets were presented (M = 927, SE = 35) vs. when five targets were presented (M = 1004, SE = 35). This is consistent with the regularity that lower set sizes are associated with shorter RTs in healthy adults [35,36]. None of the other effects were significant.

Two measures of working memory capacity were calculated. The first [34], K, was calculated as

$$K = N \times (H - F)$$

where N is the number of targets to be remembered, H is the hit rate, and F is the false alarm rate. The form of this measure guarantees that K will always be higher for five targets than for three targets. To adjust for this and provide a more interpretable measure across levels of total memory load, we also calculated a second capacity measure, $K^{'}$, which we defined as

$$K' = \frac{N \times (H - F)}{N + D}$$

where D is the number of distractors.

The repeated measures ANOVA on K revealed a main effect of number of targets, showing that participants had a higher working memory capacity when five targets were presented relative to when three targets were presented (M = 2.86, SE = 0.04). As noted above, this is a natural consequence of the way that K is defined. The repeated measures ANOVA was on K' also found a higher working memory capacity when five targets were presented (M = .44 , SE = .01) relative to when three targets were presented (M = .40, SE = .01). The magnitude of the difference, however, suggests that while the difference was significant, it most likely is not meaningful.

The repeated measures ANOVA on d' revealed a main effect of number of targets, showing that participants had a higher d' when three targets were presented (M = 3.98, SE = .15) vs. when five targets were presented (M = 2.61, SE = .23). This shows that the participants found it easier to differentiate between the presence and absence of a target when the three targets were presented, which is consistent with classic findings in the literature [37]. There were no significant effects on c.

4.4.2. Correlations between behavior and iron status

The complete set of correlations involving the behavioral variables and the iron status biomarkers and age are presented in Supplementary Tables **??**-S26. Age was negatively correlated with accuracy, but only when five targets and zero distractors were present. Age was positively correlated with RT in all conditions, indicating age-related slowing of

performance [38]. Finally, age was positively correlated with the false alarm rate, but only when five targets and zero distractors were present.

Table S26. Correlations involving the behavioral variables from the VSWM and the iron status biomarkers (3). Cells highlighted in gray indicate correlations that were signficant after correcting for the false discovery rate.

Variable	Targets	Distractors	Target pres/abs	MCHC r	MCHC p	RDW r	RDW p
Accuracy P(C)	3	0	A	-0.24	0.229	0.21	0.307
			P	0.21	0.292	-0.09	0.651
		2	A	-0.13	0.534	0.26	0.192
			P	-0.17	0.409	0.14	0.484
	5	0	A	-0.04	0.847	-0.11	0.577
			P	-0.02	0.919	0.00	0.990
		2	A	-0.07	0.740	0.13	0.533
			P	0.05	0.794	-0.41	0.040
RT, correct responses	3	0	A	0.00	0.981	0.31	0.126
			P	-0.03	0.877	0.36	0.068
		2	A	-0.03	0.894	0.37	0.064
			P	-0.03	0.883	0.31	0.123
	5	0	A	-0.11	0.590	0.32	0.106
			P	0.01	0.953	0.41	0.045
		2	A	0.04	0.850	0.27	0.185
			P	-0.06	0.759	0.41	0.039
Hit rate	3	0		0.21	0.303	-0.09	0.679
		2		-0.17	0.396	0.15	0.471
	5	0		-0.03	0.870	0.00	0.982
		2		0.05	0.793	-0.40	0.042
False alarm rate	3	0		0.25	0.226	-0.21	0.297
		2		0.14	0.494	-0.27	0.190
	5	0		0.04	0.851	0.17	0.573
		2		0.06	0.779	-0.12	0.546
K	3	0		0.19	0.358	-0.06	0.760
		2		-0.18	0.366	0.16	0.440
	5	0		-0.08	0.709	0.00	0.984
		2		0.04	0.851	-0.39	0.051
K'	3	0		-0.03	0.902	0.04	0.856
		2		-0.19	0.353	0.06	0.763
	5	0		-0.10	0.618	0.07	0.721
		2		0.06	0.769	-0.35	0.078
d'	3	0		0.01	0.960	0.03	0.897
		2		-0.11	0.608	0.19	0.347
	5	0		0.00	0.998	-0.12	0.568
		2		-0.05	0.800	-0.35	0.084
c	3	0		-0.41	0.036	0.28	0.172
		2		0.07	0.722	0.14	0.493
	5	0		-0.05	0.820	0.00	0.984
		2		-0.12	0.552	0.45	**0.022**

Accuracy, hit rates, working memory capacity (K and K'), and d' were all positively related to sFt and were more frequently positively related to sFt percentile. This indicates that numerous measures of performance were related to these measures of iron status in the same way as has been observed in women of reproductive age. In addition, higher values of both of these were related to more liberal responding, something that we have observed in previous studies with women of reproductive age. With respect to Hb and

the other iron status variables related to oxygen transport, the only correlation to reach significance after correcting for the false discovery rate was a positive relationship between *c* and RDW, indicating that a lower iron status (higher values of RDW) were associated with a more conservative response, something that we have observed in previous studies involving women of reproductive age.

4.4.3. EEG data

Group average waveforms at each level of number of targets and number of distractors at each of the three electrodes are presented in the panels of Supplementary Figure S11. These data were analyzed separately at each electrode using a set of 2 (number of targets: 3, 5) × 2 (number of distractors) × 2 (match: match, non-match) mixed models repeated measures ANOVA, with the three factors as fixed and participant as a random factor. In this section of the results "match" refers to a target present trial and "non-match" refers to a target absent trial. The results of these analysis are presented in Supplementary Table S27. None of the main effects or interactions were significant.

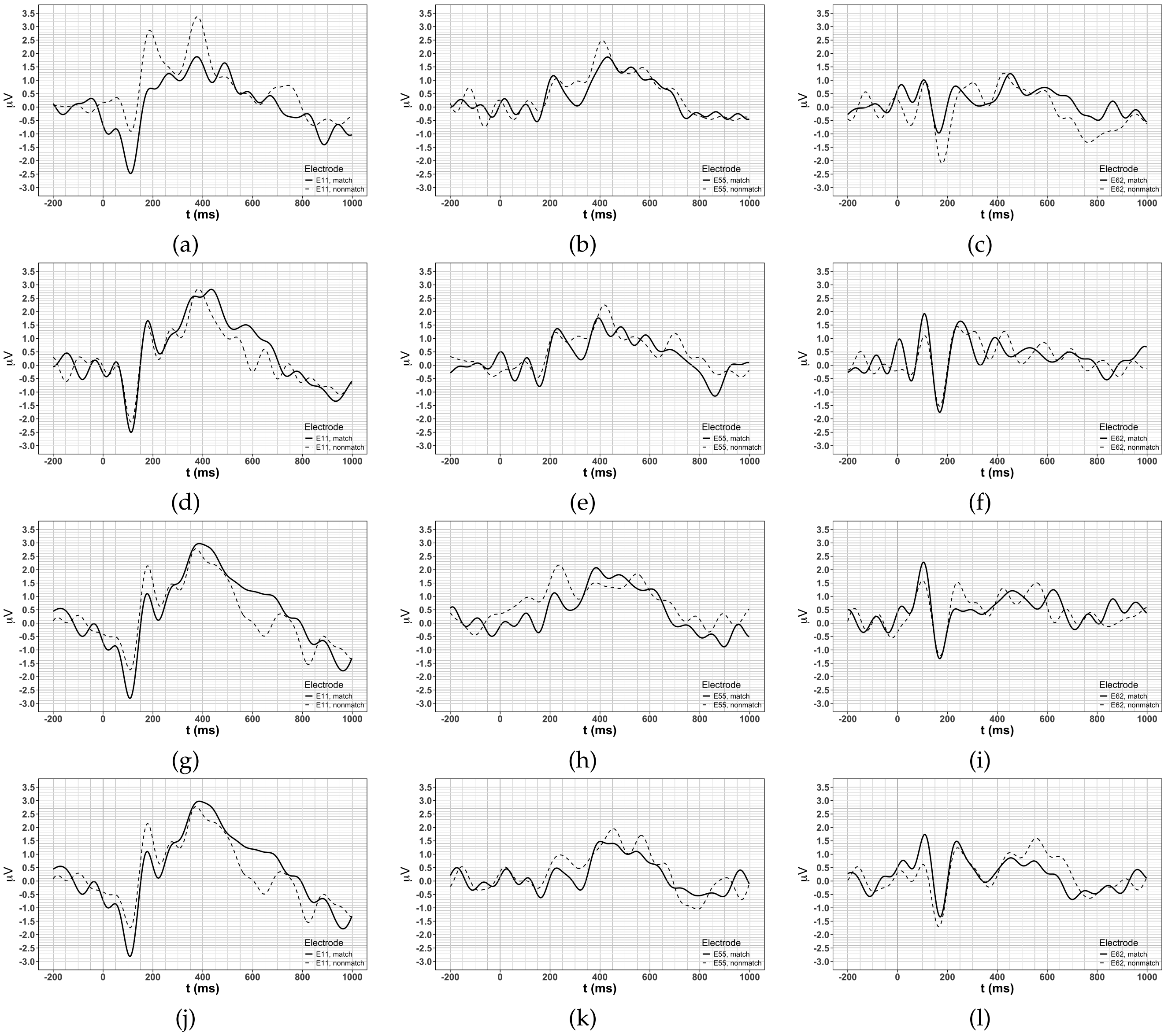

Figure S11. Group average waveforms at each level of number of targets and number of distractors at each of the three electrode: (a)-(c), 3 targets, 0 distractors; (d)-(f), 3 targets, 2 distractors; (g)-(i), 5 targets, 0 distractors; (j)-(l) 5 targets, 2 distractors. Solid lines are from target present trials and dashed lines are from target absent trials.

Table S27. Repeated measures analyses of the EEG data from the VSWM.

Electrode	Factor	*F*	*p*
E11	Targets (T)	2.40	0.124
	Distractors (D)	0.40	0.527
	Match (M)	3.26	0.073
	T × D	0.01	0.907
	T × M	0.42	0.520
	D × *M*	0.01	0.927
	T × D × M	1.68	0.197
E55	T	0.45	0.501
	D	3.30	0.071
	M	2.12	0.148
	T × D	2.16	0.144
	T × M	0.00	0.963
	D × M	0.11	0.743
	T × D × M	0.06	0.810
E62	T	0.14	0.708
	D	0.01	0.920
	M	0.45	0.505
	T × D	0.70	0.405
	T × M	0.01	0.940
	D × M	0.01	0.941
	T × D × M	0.39	0.531

4.4.4. VSWM EEG correlations

The complete set of correlations involving the EEG variables from the VSWM and the iron status biomarkers and age are presented in Supplementary Tables S28-S30. The only correlation involving age was for amplitudes at electrode E11 on trials on which three targets and 0 distractors. That relationship was positive, which runs counter to expectations. There were a small number of positive correlations between amplitudes and sFt, with these indicating that higher sFt values were associated with larger amplitudes, a result that is consistent with prior work with women of reproductive age [31,33]. There were no significant relationships between amplitudes at any of the electrodes and sFt percentile. There were no significant correlations involving Hb. However, there were a small number of significant correlations involving amplitude and either MCH and MCHC, indicating that there was some evidence for a positive relationship between these iron status biomarkers—related to oxygen transport—and brain function.

Table S28. Correlations involving the EEG variables from the VSWM from the iron status biomarkers (1). Cells highlighted in gray indicate correlations that were significant after correcting for the false discovery rate.

Targets	Distractors	Match	Variable	Electrode	*r*	*p*	Targets	Distractors	Match	*r*	*p*
3	0	0	Age	E11	0.49	**0.021**	5	0	0	0.30	0.180
				E55	0.14	0.531				0.16	0.482
				E62	0.06	0.777				0.29	0.198
			Hb	E11	0.12	0.607				0.13	0.574
				E55	-0.22	0.329				-0.10	0.664
				E62	-0.22	0.324				-0.18	0.415
			sFt	E11	0.22	0.315				0.33	0.128
				E55	0.36	0.104				-0.03	0.882
				E62	0.10	0.644				-0.15	0.509
			sFt_pctl	E11	0.04	0.871				0.13	0.565
				E55	0.32	0.149				-0.10	0.646
				E62	0.13	0.564				-0.24	0.286
		1	Age	E11	0.22	0.322			1	0.24	0.287
				E55	-0.27	0.227				0.14	0.533
				E62	-0.04	0.843				0.18	0.428
			Hb	E11	-0.03	0.891				0.13	0.571
				E55	0.33	0.134				-0.24	0.274
				E62	0.09	0.688				-0.19	0.401
			sFt	E11	0.58	**0.004**				0.45	0.034
				E55	0.34	0.118				0.12	0.596
				E62	-0.04	0.855				-0.19	0.396
			sFt_pctl	E11	0.43	0.048				0.33	0.134
				E55	0.45	0.036				0.07	0.768
				E62	0.04	0.855				-0.28	0.199
	2	0	Age	E11	0.18	0.410		2	0	0.04	0.861
				E55	0.01	0.972				0.13	0.568
				E62	-0.08	0.726				0.15	0.496
			Hb	E11	0.08	0.725				0.00	0.996
				E55	0.21	0.341				-0.26	0.237
				E62	-0.01	0.976				-0.10	0.662
			sFt	E11	0.48	**0.023**				0.26	0.250
				E55	0.00	0.983				0.01	0.981
				E62	-0.08	0.735				-0.04	0.873
			sFt_pctl	E11	0.38	0.079				0.26	0.236
				E55	-0.05	0.820				-0.06	0.808
				E62	-0.01	0.973				-0.12	0.596
		1	Age	E11	0.10	0.665			1	0.12	0.601
				E55	-0.02	0.922				0.56	**0.007**
				E62	0.08	0.725				0.42	0.054
			Hb	E11	0.21	0.339				0.02	0.921
				E55	-0.25	0.266				-0.05	0.841
				E62	-0.18	0.423				0.11	0.623
			sFt	E11	0.48	**0.025**				0.51	**0.015**
				E55	0.04	0.856				0.34	0.122
				E62	-0.10	0.670				0.23	0.312
			sFt_pctl	E11	0.42	0.054				0.40	0.069
				E55	0.03	0.879				0.06	0.793
				E62	-0.11	0.613				-0.01	0.973

Table S29. Correlations involving the EEG variables from the VSWM from the iron status biomarkers (2). Cells highlighted in gray indicate correlations that were significant after correcting for the false discovery rate.

Targets	Distractors	Match	Variable	Electrode	*r*	*p*	Targets	Distractors	Match	*r*	*p*
3	0	0	RBC	E11	-0.01	0.978	5	0	0	0.05	0.815
				E55	-0.07	0.750				0.03	0.889
				E62	-0.08	0.720				-0.20	0.369
			HCT	E11	-0.03	0.904				0.04	0.852
				E55	-0.25	0.258				-0.14	0.537
				E62	-0.21	0.339				-0.20	0.371
			RDW	E11	-0.07	0.770				-0.02	0.918
				E55	-0.10	0.673				0.00	0.984
				E62	-0.27	0.223				0.02	0.929
		1	RBC	E11	-0.08	0.733			1	0.09	0.700
				E55	0.07	0.773				-0.14	0.533
				E62	-0.26	0.250				-0.16	0.485
			HCT	E11	-0.11	0.611				0.04	0.854
				E55	0.21	0.358				-0.26	0.241
				E62	-0.02	0.919				-0.11	0.640
			RDW	E11	-0.14	0.531				-0.14	0.526
				E55	-0.32	0.145				0.08	0.717
				E62	-0.34	0.123				0.23	0.295
	2	0	RBC	E11	-0.22	0.319		2	0	-0.33	0.132
				E55	0.20	0.365				-0.25	0.263
				E62	-0.01	0.950				-0.04	0.877
			HCT	E11	-0.06	0.807				-0.19	0.390
				E55	0.16	0.485				-0.13	0.571
				E62	-0.11	0.634				0.08	0.720
			RDW	E11	-0.27	0.216				-0.23	0.295
				E55	0.04	0.876				0.21	0.341
				E62	-0.24	0.288				0.23	0.294
		1	RBC	E11	0.05	0.834			1	-0.16	0.482
				E55	-0.27	0.230				0.23	0.307
				E62	-0.31	0.160				0.10	0.661
			HCT	E11	0.10	0.657				-0.11	0.617
				E55	-0.31	0.162				0.08	0.724
				E62	-0.24	0.272				0.20	0.376
			RDW	E11	-0.13	0.572				-0.06	0.775
				E55	-0.03	0.884				0.19	0.400
				E62	0.05	0.836				-0.08	0.721

Table S30. Correlations involving the EEG variables from the VSWM from the iron status biomarkers (2). Cells highlighted in gray indicate correlations that were significant after correcting for the false discovery rate.

Targets	Distractors	Match	Variable	Electrode	*r*	*p*	Targets	Distractors	Match	*r*	*p*
3	0	0	MCV	E11	-0.03	0.901	5	0	0	-0.02	0.932
				E55	-0.23	0.304				-0.24	0.274
				E62	-0.16	0.476				0.05	0.830
			MCH	E11	0.16	0.465				0.10	0.643
				E55	-0.20	0.363				-0.19	0.398
				E62	-0.19	0.407				0.03	0.893
			MCHC	E11	0.43	0.045				0.27	0.223
				E55	0.02	0.924				0.06	0.775
				E62	-0.09	0.690				-0.03	0.901
		1	MCV	E11	-0.03	0.901			1	-0.07	0.747
				E55	0.15	0.499				-0.14	0.549
				E62	0.35	0.106				0.11	0.635
			MCH	E11	0.07	0.744				0.06	0.774
				E55	0.34	0.127				-0.14	0.527
				E62	0.47	**0.027**				-0.04	0.870
			MCHC	E11	0.23	0.306				0.30	0.180
				E55	0.47	**0.027**				-0.04	0.846
				E62	0.34	0.123				-0.29	0.183
	2	0	MCV	E11	0.28	0.214		2	0	0.27	0.232
				E55	-0.12	0.601				0.21	0.342
				E62	-0.11	0.619				0.16	0.477
			MCH	E11	0.42	0.049				0.47	**0.027**
				E55	-0.01	0.966				-0.01	0.958
				E62	0.01	0.953				-0.08	0.716
			MCHC	E11	0.40	0.067				0.52	**0.013**
				E55	0.23	0.313				0.46	0.033
				E62	0.26	0.243				0.51	**0.016**
		1	MCV	E11	0.07	0.773			1	0.10	0.667
				E55	-0.01	0.968				-0.27	0.233
				E62	0.15	0.519				0.08	0.736
			MCH	E11	0.23	0.294				0.25	0.258
				E55	0.02	0.937				-0.39	0.076
				E62	0.17	0.452				-0.02	0.947
			MCHC	E11	0.40	0.066				0.38	0.081
				E55	0.06	0.779				-0.33	0.134
				E62	0.34	0.123				-0.18	0.433

4.5. Blink rates

The complete set of correlations between task-related and spontaneous blink rates (from the resting period) are presented in Supplementary Table S31. There were no significant correlations with age. Task-related blink rates in each of the tasks were all significantly negatively related to blink rates, with this consistent with the idea that greater cognitive engagement in a task is related to lower blink rates [39,40]. Task-related blink rates in both the FNAM and RBCL were negatively related to blink rates, and task-related blink rates in the FNAM, PST, and RBCL, were negatively related to at least one other biomarker associated with oxygen transport.

Table S31. Correlations involving task-related and spontaneous blink rates and the iron status biomarkers and age. Cells highlighted in gray indicate correlations that were significant after correcting for the false discovery rate.

	FNAM		PST		RBCL		VSWM		Resting	
	r	*p*	*r*	*p*	*r*	*p*	*r*	*p*	*r*	*p*
Age	0.21	0.323	0.15	0.465	0.15	0.47	0.09	0.668	0.00	0.986
Hb	-0.49	**0.016**	-0.25	0.217	-0.50	**0.011**	-0.36	0.081	0.00	0.986
sFt	-0.57	**0.004**	-0.47	**0.017**	-0.43	**0.032**	-0.53	**0.006**	-0.34	0.108
sFt percentile	-0.60	**0.002**	-0.47	**0.015**	-0.45	**0.025**	-0.54	**0.006**	-0.38	0.077
RBC	-0.24	0.623	-0.22	0.368	-0.39	0.056	-0.31	0.138	-0.11	0.626
Hct	-0.41	0.062	-0.20	**0.032**	-0.37	0.068	-0.36	0.079	0.05	0.815
RDW	0.51	**0.007**	0.21	0.050	0.29	0.154	0.24	0.253	0.11	0.611
MCV	-0.33	0.470	-0.27	0.751	0.13	0.551	0.00	0.985	0.21	0.330
MCH	-0.39	0.132	-0.33	0.816	-0.11	0.597	-0.05	0.797	0.13	0.561
MCHC	-0.19	0.083	-0.18	0.276	-0.47	**0.017**	-0.13	0.528	-0.14	0.529

4.6. MRI estimates of brain iron deposits

Initial inspection of the intensity values from each of the ROIs indicated that the data were not normally distributed and that variances were quite different across ROIs. Therefore, the intensity values were transformed using the Box-Cox transformation

$$y' = \frac{y^{\lambda} - 1}{\lambda}, y \neq 0$$

with $\lambda = 0.22$. Group averages for transformed intensity values in each ROI in each hemisphere are presented in Supplementary Figure S12. As a reminder, lower intensity values are indicative of higher iron deposits. These data were analyzed using a 2 (hemisphere: left, right) $\times$ 6 (ROI: caudate, putamen, globus pallidus, substantia nigra, VLPFC, ACC) mixed model repeated measures ANOVA, with hemisphere and ROI as fixed factors and participant as the random factor. The results of this analysis are presented in Supplementary Table S32. There was a significant main effect of hemisphere and a significant hemisphere $\times$ ROI interaction, with post-hoc Tukey tests indicating that intensity values were lowest in substantia nigra, globus pallidus, caudate, and putamen being lower (higher in iron) than VLPFC which in turn was lower than ACC, with the ordering of the lowest values being different in each hemisphere. These results are consistent with previous literature on brain iron deposits [27,28,41,42].

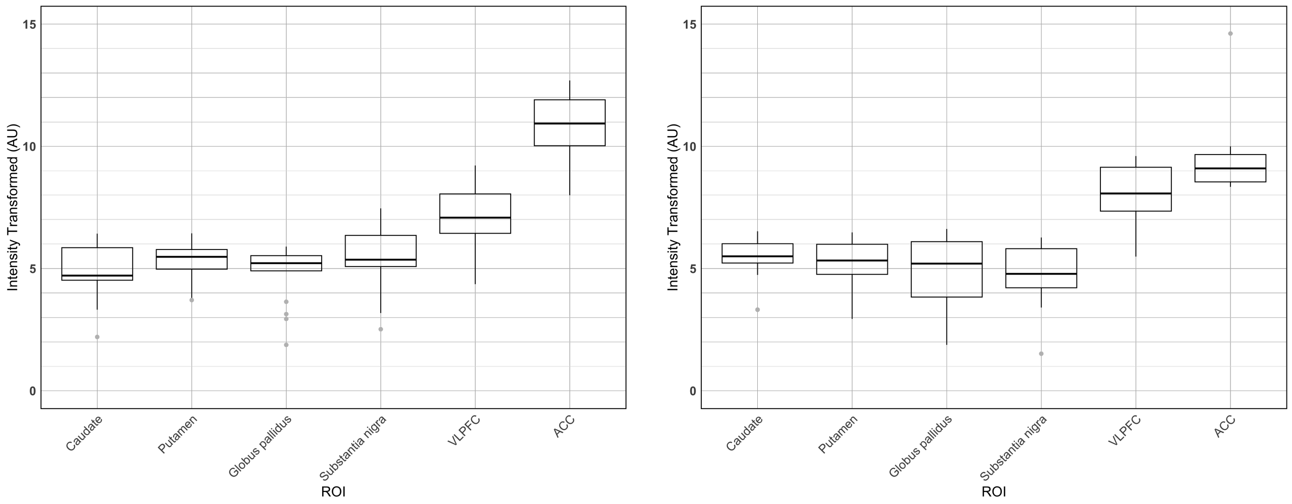

(a) (b)

Figure S12. Transformed intensity values for each of the ROIs in (a) the left and (b) the right hemisphere. Note: AU = arbitrary units.

Table S32. Repeated measures analysis of the transformed MRI intensity values.

Factor	*df*	*F*	*p*
Hemisphere (H)	1	0.35	0.556
ROI (R)	5	103.79	< 0.001
H × R	5	3.79	0.003

The complete set of correlations involving the MRI estimates of brain iron deposits and the blood iron status biomarkers are presented in Supplementary Tables S33-S35. In brief, there were no significant correlations observed.

Table S33. Correlations involving transformed MRI intensity values and blood iron biomarkers (1).

		Age		Hb		sFt		sFt pctl	
Hemisphere	ROI	*r*	*p*	*r*	*p*	*r*	*p*	*r*	*p*
Left	Caudate	-0.07	0.794	-0.19	0.470	0.20	0.443	0.18	0.496
	Putamen	-0.32	0.217	0.22	0.401	-0.26	0.317	-0.11	0.673
	Globus pallidus	0.21	0.426	-0.35	0.173	-0.32	0.208	-0.36	0.161
	Substantia nigra	-0.01	0.966	0.17	0.519	-0.15	0.578	-0.16	0.536
	VLPFC	0.30	0.247	0.20	0.444	-0.25	0.335	-0.28	0.269
	ACC	0.28	0.286	0.04	0.877	-0.13	0.633	-0.30	0.240
Right	Caudate	0.05	0.860	0.18	0.482	-0.23	0.369	-0.24	0.358
	Putamen	-0.23	0.366	-0.02	0.942	0.12	0.646	0.15	0.575
	Globus pallidus	0.28	0.269	0.19	0.466	-0.20	0.439	-0.27	0.287
	Substantia nigra	0.11	0.691	-0.21	0.446	-0.30	0.262	-0.36	0.172
	VLPFC	0.11	0.663	0.35	0.164	-0.29	0.259	-0.27	0.293
	ACC	-0.09	0.724	-0.12	0.661	-0.32	0.211	-0.49	0.045

Table S34. Correlations involving transformed MRI intensity values and blood iron biomarkers (2).

		RBC		HCT		RDW	
Hemisphere	ROI	*r*	*p*	*r*	*p*	*r*	*p*
Left	Caudate	0.02	0.943	-0.34	0.183	-0.15	0.571
	Putamen	-0.08	0.757	0.29	0.252	-0.21	0.411
	Globus pallidus	-0.11	0.680	-0.19	0.476	0.41	0.103
	Substantia nigra	0.30	0.236	0.09	0.746	0.37	0.142
	VLPFC	0.07	0.790	0.32	0.217	-0.12	0.655
	ACC	-0.02	0.955	0.21	0.422	0.05	0.865
Right	Caudate	0.41	0.105	0.07	0.789	0.12	0.659
	Putamen	-0.31	0.230	-0.10	0.705	-0.15	0.572
	Globus pallidus	0.34	0.179	0.21	0.428	0.15	0.580
	Substantia nigra	-0.01	0.970	-0.25	0.342	-0.33	0.209
	VLPFC	0.25	0.326	0.49	0.047	-0.21	0.430
	ACC	0.40	0.117	0.36	0.155	-0.13	0.613

Table S35. Correlations involving transformed MRI intensity values and blood iron biomarkers (3).

		MCV		MCH		MCHC	
Hemisphere	ROI	*r*	*p*	*r*	*p*	*r*	*p*
Left	Caudate	-0.41	0.100	-0.27	0.299	0.21	0.417
	Putamen	0.44	0.080	0.36	0.153	-0.04	0.880
	Globus pallidus	-0.08	0.768	-0.33	0.201	-0.50	0.042
	Substantia nigra	-0.27	0.300	-0.12	0.653	0.24	0.361
	VLPFC	0.27	0.291	0.17	0.523	-0.14	0.588
	ACC	0.25	0.333	0.07	0.797	-0.32	0.209
Right	Caudate	-0.41	0.101	-0.21	0.414	0.31	0.231
	Putamen	0.27	0.292	0.33	0.202	0.13	0.615
	Globus pallidus	-0.19	0.460	-0.14	0.593	0.07	0.782
	Substantia nigra	-0.32	0.222	-0.33	0.217	-0.01	0.969
	VLPFC	0.23	0.381	0.15	0.563	-0.10	0.702
	ACC	-0.07	0.782	0.07	0.786	0.28	0.273

References

1. Amariglio, R.E.; Becker, J.A.; Carmasin, J.; Wadsworth, L.P.; Lorius, N.; Sullivan, C.; Maye, J.E.; Gidicsin, C.; Pepin, L.C.; Sperling, R.A.; et al. Subjective cognitive complaints and amyloid burden in cognitively normal older individuals. *Neuropsychologia* **2012**, *50*, 2880–2886.
2. Jessen, F.; Amariglio, R.E.; Van Boxtel, M.; Breteler, M.; Ceccaldi, M.; Chételat, G.; Dubois, B.; Dufouil, C.; Ellis, K.A.; Van Der Flier, W.M.; et al. A conceptual framework for research on subjective cognitive decline in preclinical Alzheimer's disease. *Alzheimer's & Dementia* **2014**, *10*, 844–852.
3. Rentz, D.M.; Amariglio, R.E.; Becker, J.A.; Frey, M.; Olson, L.E.; Frishe, K.; Carmasin, J.; Maye, J.E.; Johnson, K.A.; Sperling, R.A. Face-name associative memory performance is related to amyloid burden in normal elderly. *Neuropsychologia* **2011**, *49*, 2776–2783.
4. Rubiño, J.; Andrés, P. The face-name associative memory test as a tool for early diagnosis of Alzheimer's disease. *Frontiers in Psychology* **2018**, *9*, 1464.
5. Frank, M.J.; Seeberger, L.C.; O'Reilly, R.C. By carrot or by stick: Cognitive reinforcement learning in Parkinsonism. *Science* **2004**, *306*, 1940–1943.
6. Frank, M.J.; Samanta, J.; Moustafa, A.A.; Sherman, S.J. Hold Your Horses: Impulsivity, Deep Brain Stimulation, and Medication in Parkinsonism. *Science* **2007**, *318*, 1309 – 1312. https://doi.org/10.2307/20051661.
7. Ashby, F.G.; Noble, S.; Filoteo, J.V.; Waldron, E.M.; Ell, S.W. Category learning deficits in Parkinson's disease. *Neuropsychology* **2003**, *17*, 115.
8. Hélie, S.; Waldschmidt, J.G.; Ashby, F.G. Automaticity in rule-based and information-integration categorization. *Attention, Perception, & Psychophysics* **2010**, *72*, 1013–1031.
9. Lewandowsky, S.; Yang, L.X.; Newell, B.R.; Kalish, M.L. Working memory does not dissociate between different perceptual categorization tasks. *Journal of Experimental Psychology: Learning, Memory, and Cognition* **2012**, *38*, 881.
10. Maddox, W.T.; Ashby, F.G.; Bohil, C.J. Delayed feedback effects on rule-based and information-integration category learning. *Journal of Experimental Psychology: Learning, Memory, and Cognition* **2003**, *29*, 650.
11. Ashby, F.G.; Wang, Y.W. Computational cognitive neuroscience models of categorization. In *The Cambridge Handbook of Computational Cognitive Sciences*, 2nd edition ed.; Sun, R., Ed.; Cambridge University Press, 2023; chapter 12, pp. 400–425.
12. Cools, R.; Altamirano, L.; D'Esposito, M. Reversal learning in Parkinson's disease depends on medication status and outcome valence. *Neuropsychologia* **2006**, *44*, 1663–1673.
13. Costa, A.; Peppe, A.; Dell'Agnello, G.; Carlesimo, G.A.; Murri, L.; Bonuccelli, U.; Caltagirone, C. Dopaminergic modulation of visual-spatial working memory in Parkinson's disease. *Dementia and Geriatric Cognitive Disorders* **2003**, *15*, 55–66.
14. Moustafa, A.A.; Sherman, S.J.; Frank, M.J. A dopaminergic basis for working memory, learning and attentional shifting in Parkinsonism. *Neuropsychologia* **2008**, *46*, 3144–3156.
15. Knott, V.; Millar, A.; Dulude, L.; Bradford, L.; Alwahhabi, F.; Lau, T.; Shea, C.; Wiens, A. Event-Related Potentials in Young and Elderly Adults during a Visual Spatial Working Memory Task. *Clinical EEG and Neuroscience* **2004**, *35*, 185–192.

16. Delorme, A.; Mackeig, S. EEGLAB: an open source toolbox for analysis of single-trial EEG dynamics including independent component analysis. *Journal of Neuroscience Methods* **2004**, *134*, 9–21.
17. Delorme, A. EEG is better left alone. *Scientific Reports* **2023**, *13*, 2372.
18. Delorme, A.; Jung, T.P.; Sejnowski, T.; Makeig, S.; et al. Improved rejection of artifacts from EEG data using high-order statistics and independent component analysis. *Neuroimage* **2005**.
19. Guo, C.; Voss, J.L.; Paller, K.A. Electrophysiological correlates of forming memories for faces, names, and face–name associations. *Cognitive Brain Research* **2005**, *22*, 153–164.
20. Mitchell, M.B.; Shirk, S.D.; McLaren, D.G.; Dodd, J.S.; Ezzati, A.; Ally, B.A.; Atri, A. Recognition of faces and names: Multimodal physiological correlates of memory and executive function. *Brain Imaging and Behavior* **2016**, *10*, 408–423.
21. Frank, M.J.; Woroch, B.S.; Curran, T. Error-related negativity predicts reinforcement learning and conflict biases. *Neuron* **2005**, *47*, 495–501.
22. Larson, M.J.; Clayson, P.E.; Primosch, M.; Leyton, M.; Steffensen, S.C. The effects of acute dopamine precursor depletion on the cognitive control functions of performance monitoring and conflict processing: an event-related potential (ERP) study. *PLoS one* **2015**, *10*, e0140770.
23. Schmid, P.C.; Hackel, L.M.; Jasperse, L.; Amodio, D.M. Frontal cortical effects on feedback processing and reinforcement learning: Relation of EEG asymmetry with the feedback-related negativity and behavior. *Psychophysiology* **2018**, *55*, e12911.
24. Rabi, R.; Joanisse, M.F.; Zhu, T.; Minda, J.P. Cognitive changes in conjunctive rule-based category learning: An ERP approach. *Cognitive, Affective, & Behavioral Neuroscience* **2018**, *18*, 1034–1048.
25. Kleifges, K.; Bigdely-Shamlo, N.; Kerick, S.E.; Robbins, K.A. BLINKER: Automated extraction of ocular indices from EEG enabling large-scale analysis. *Frontiers in Neuroscience* **2017**, *11*, 12.
26. Liu, M.; Liu, S.; Ghassaban, K.; Zheng, W.; Dicicco, D.; Miao, Y.; Habib, C.; Jazmati, T.; Haacke, E.M. Assessing global and regional iron content in deep gray matter as a function of age using susceptibility mapping. *Journal of Magnetic Resonance Imaging* **2016**, *44*, 59–71.
27. Daugherty, A.M.; Haacke, E.M.; Raz, N. Striatal Iron Content Predicts Its Shrinkage and Changes in Verbal Working Memory after Two Years in Healthy Adults. *The Journal of Neuroscience* **2015**, *35*, 6731–6743.
28. Daugherty, A.M.; Raz, N. Accumulation of iron in the putamen predicts its shrinkage in healthy older adults: A multi-occasion longitudinal study. *NeuroImage* **2016**, *128*, 11–20.
29. Newbolds, S.F.; Wenger, M.J. Assessing the pattern electroretinogram as a proxy measure for dopamine in the context of iron deficiency. *Nutritional Neuroscience* **2024**, pp. 1–12. https://doi.org/10.1080/1028415X.2024.2304943.
30. Rhoten, S.E.; Wenger, M.J.; De Stefano, L.A. Iron deficiency negatively affects behavioral measures of learning, indirect neural measures of dopamine, and neural efficiency. *Cognitive, Affective, and Behavioral Neuroscience* **2024**. in press.
31. Wenger, M.J.; Rhoten, S.E.; Murray-Kolb, L.E.; Scott, S.P.; Boy, E.; Gahutu, J.B.; Haas, J.D. Changes in iron status are related to changes in brain activity and behavior in Rwandan female university students: Results from a randomized controlled efficacy trial involving iron-biofortified beans. *Journal of Nutrition* **2019**, *149*, 687–697.
32. Wenger, M.J.; Murray-Kolb, L.E.; Nevins, J.E.H.; Venkatramanan, S.; Reinhart, G.A.; Wesley, A.; Haas, J.D. Consumption of a double-fortified salt affects perceptual, attentional, and mnemonic functioning in women in a randomized controlled trial in India. *The Journal of Nutrition* **2017**, *147*, 2297–2308.
33. Wenger, M.J.; Murray Kolb, L.E.; Scott, S.P.; Boy, E.; Haas, J.D. Modeling relationships between iron status, behavior, and brain electrophysiology: evidence from a randomized study involving a biofortified grain in Indian adolescents. *BMC Public Health* **2022**, *22*, 1299.
34. Rouder, J.N.; Morey, R.D.; Morey, C.C.; Cowan, N. How to measure working memory capacity in the change detection paradigm. *Psychonomic Bulletin & Review* **2011**, *18*, 324–330.
35. Sternberg, S. High-speed scanning in human memory. *Science* **1966**, *153*, 652–654.
36. Sternberg, S. The discovery of processing stages: Extensions of Donders' method. *Acta psychologica* **1969**, *30*, 276–315.
37. Kinnell, A.; Dennis, S. The role of stimulus type in list length effects in recognition memory. *Memory & Cognition* **2012**, *40*, 311–325.
38. Ratcliff, R.; Spieler, D.; McKoon, G. Explicitly modeling the effects of aging on response time. *Psychonomic Bulletin and Review* **2000**, *7*, 1–25.
39. Ranti, C.; Jones, W.; Klin, A.; Shultz, S. Blink rate patterns provide a reliable measure of individual engagement with scene content. *Scientific Reports* **2020**, *10*, 8267.

40. Rosenfield, M.; Jahan, S.; Nunez, K.; Chan, K. Cognitive demand, digital screens and blink rate. *Computers in Human Behavior* **2015**, *51*, 403–406.
41. Connor, J.; Menzies, S.; St Martin, S.; Mufson, E. Cellular distribution of transferrin, ferritin, and iron in normal and aged human brains. *Journal of neuroscience research* **1990**, *27*, 595–611.
42. Connor, J.R.; Snyder, B.S.; Beard, J.L.; Fine, R.E.; Mufson, E.J. Regional distribution of iron and iron-regulatory proteins in the brain in aging and Alzheimer's disease. *Journal of Neuroscience Research* **1992**, *31*, 327–335.

Disclaimer/Publisher's Note: The statements, opinions and data contained in all publications are solely those of the individual author(s) and contributor(s) and not of MDPI and/or the editor(s). MDPI and/or the editor(s) disclaim responsibility for any injury to people or property resulting from any ideas, methods, instructions or products referred to in the content.
