# Supplementary figures and images for: Cognitive Performance in Relation to Systemic and Brain Iron at Perimenopause
Source: Nutrients. 2025 Feb 20;17(5):745. doi: 10.3390/nu17050745 (PMC11901746; doi:10.3390/nu17050745)

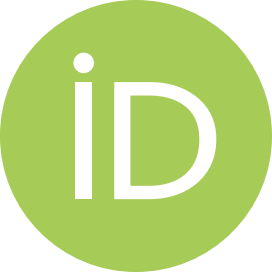

Supplement: Supplementary file 1 [file nutrients-17-00745-s001.zip › Definitions/logo-orcid.pdf]

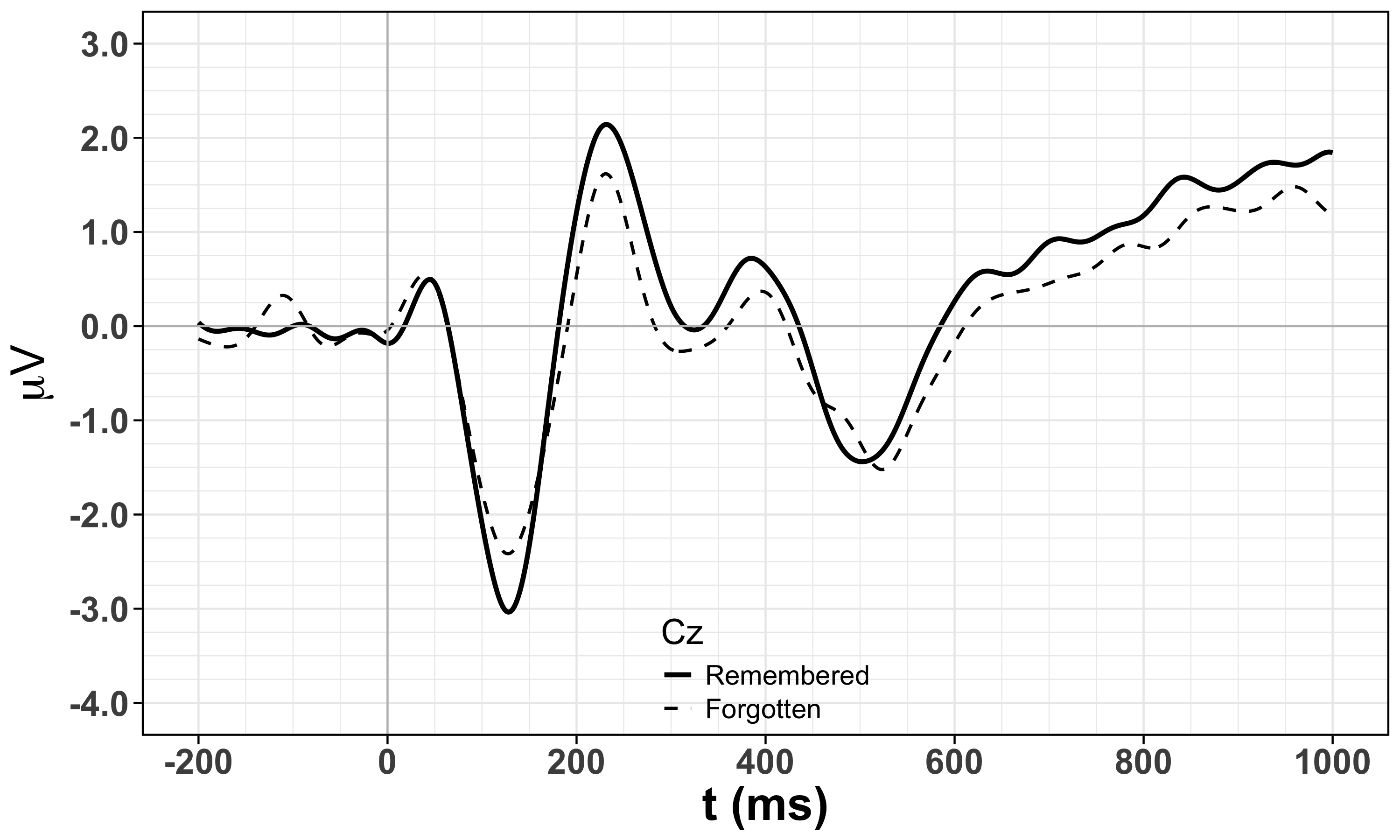

Supplement: Supplementary file 1 [file nutrients-17-00745-s001.zip › figures/fnam_name_del_Cz.png]

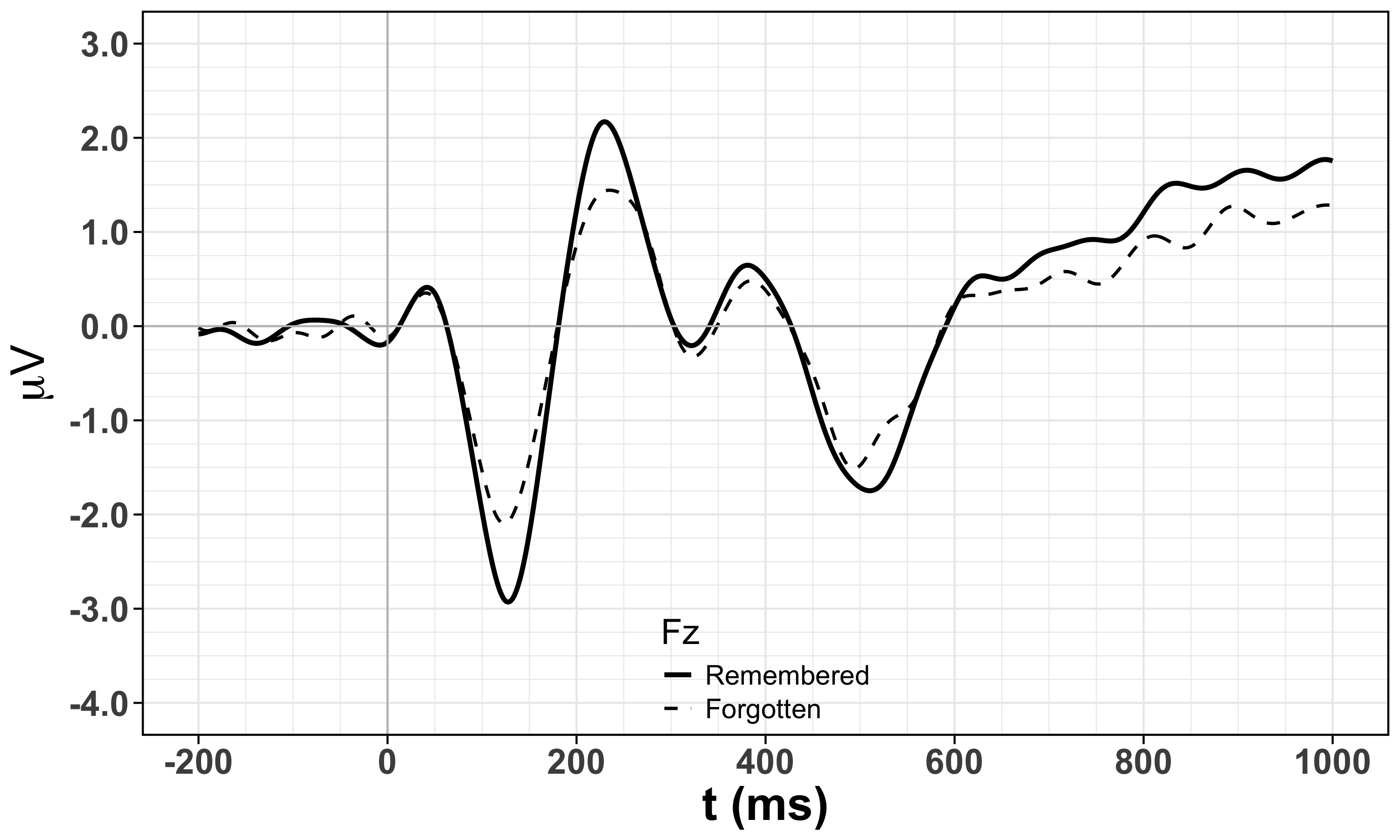

Supplement: Supplementary file 1 [file nutrients-17-00745-s001.zip › figures/fnam_name_del_Fz.png]

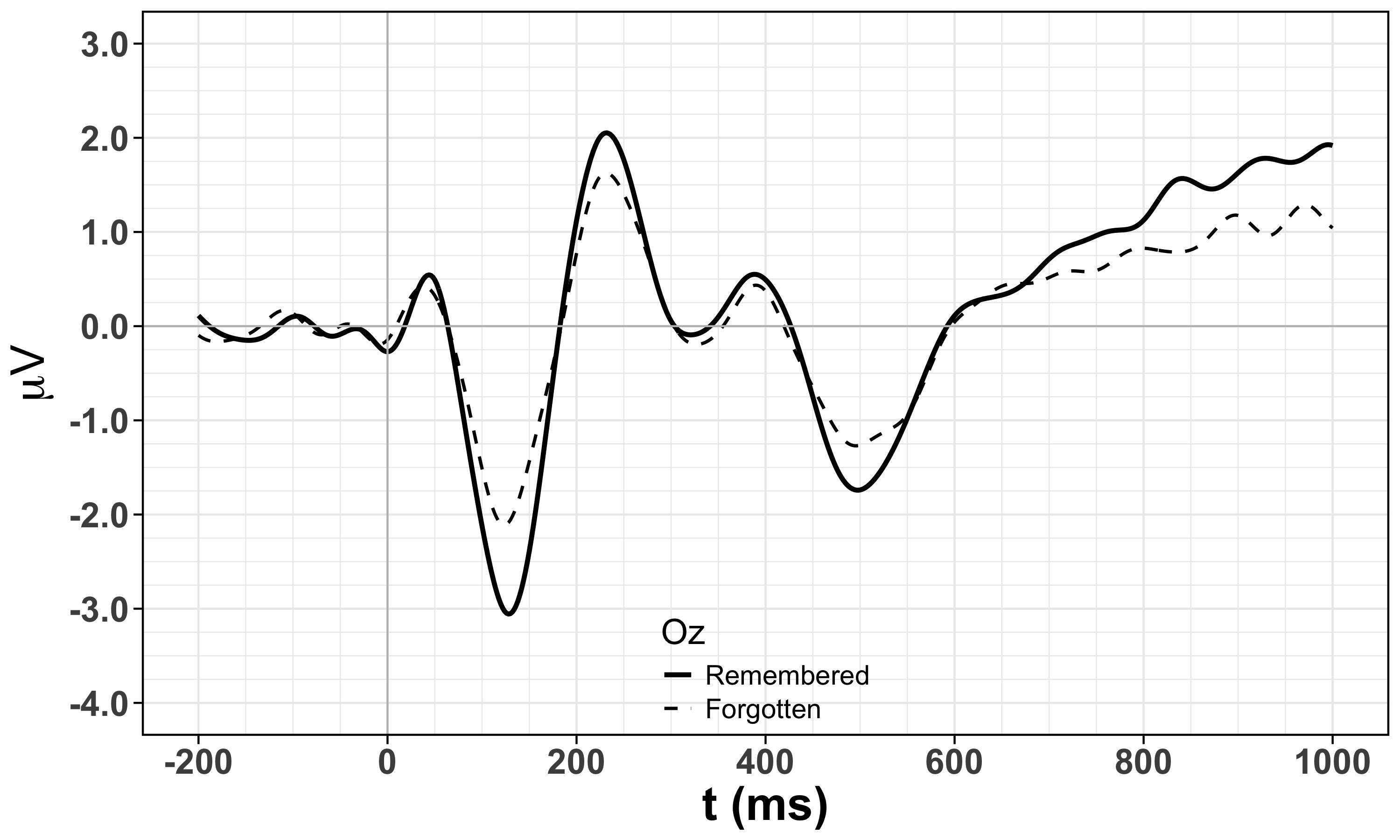

Supplement: Supplementary file 1 [file nutrients-17-00745-s001.zip › figures/fnam_name_del_Oz.png]

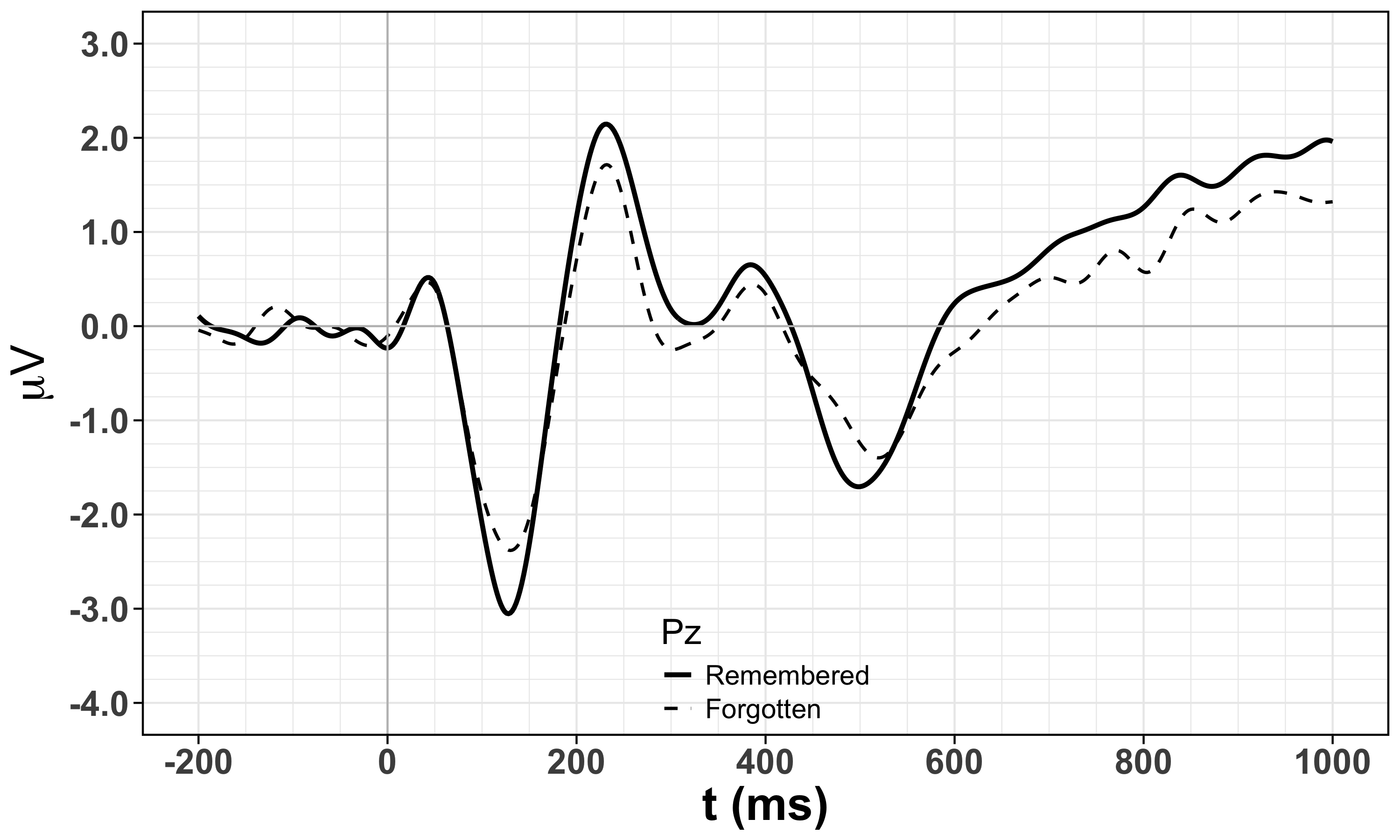

Supplement: Supplementary file 1 [file nutrients-17-00745-s001.zip › figures/fnam_name_del_Pz.png]

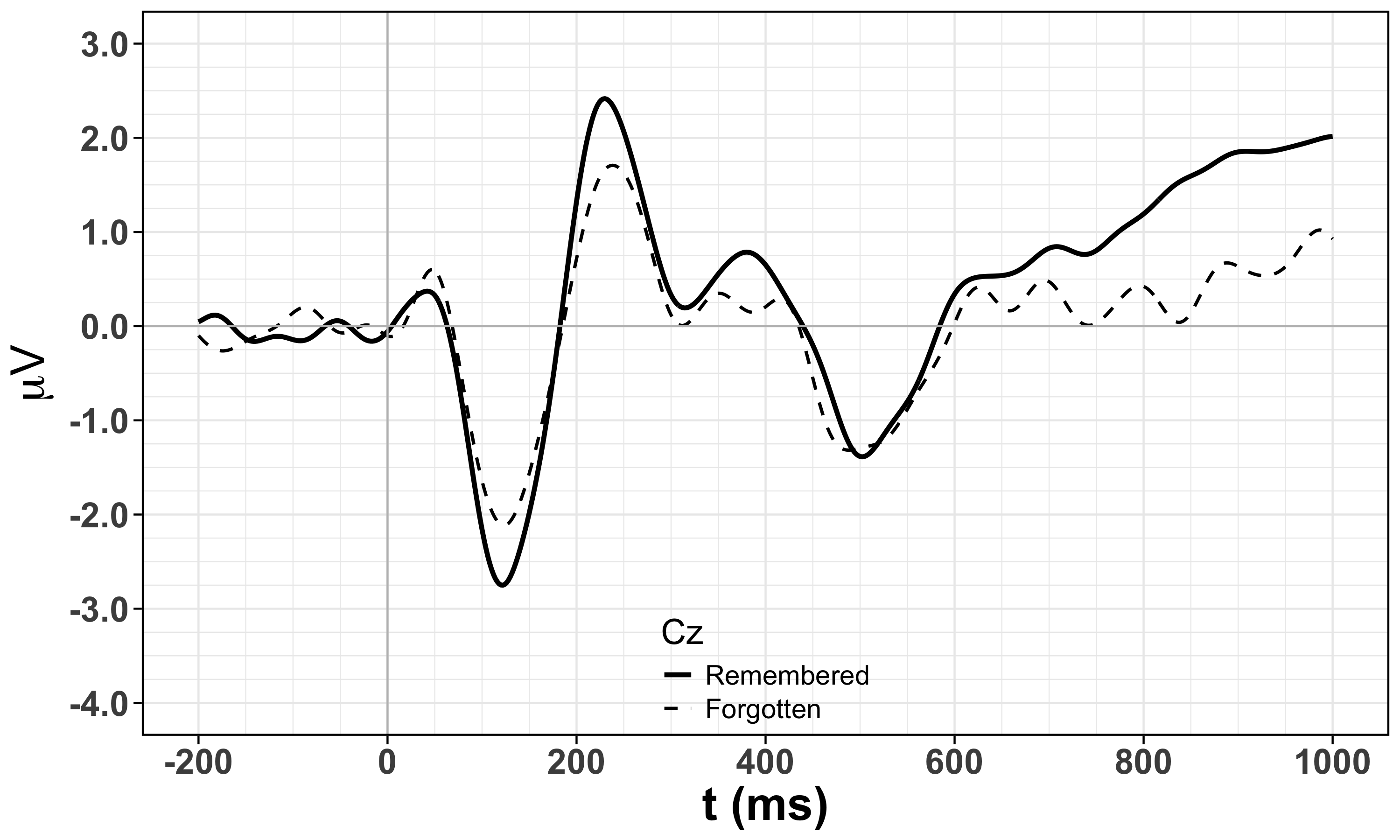

Supplement: Supplementary file 1 [file nutrients-17-00745-s001.zip › figures/fnam_name_imm_Cz.png]

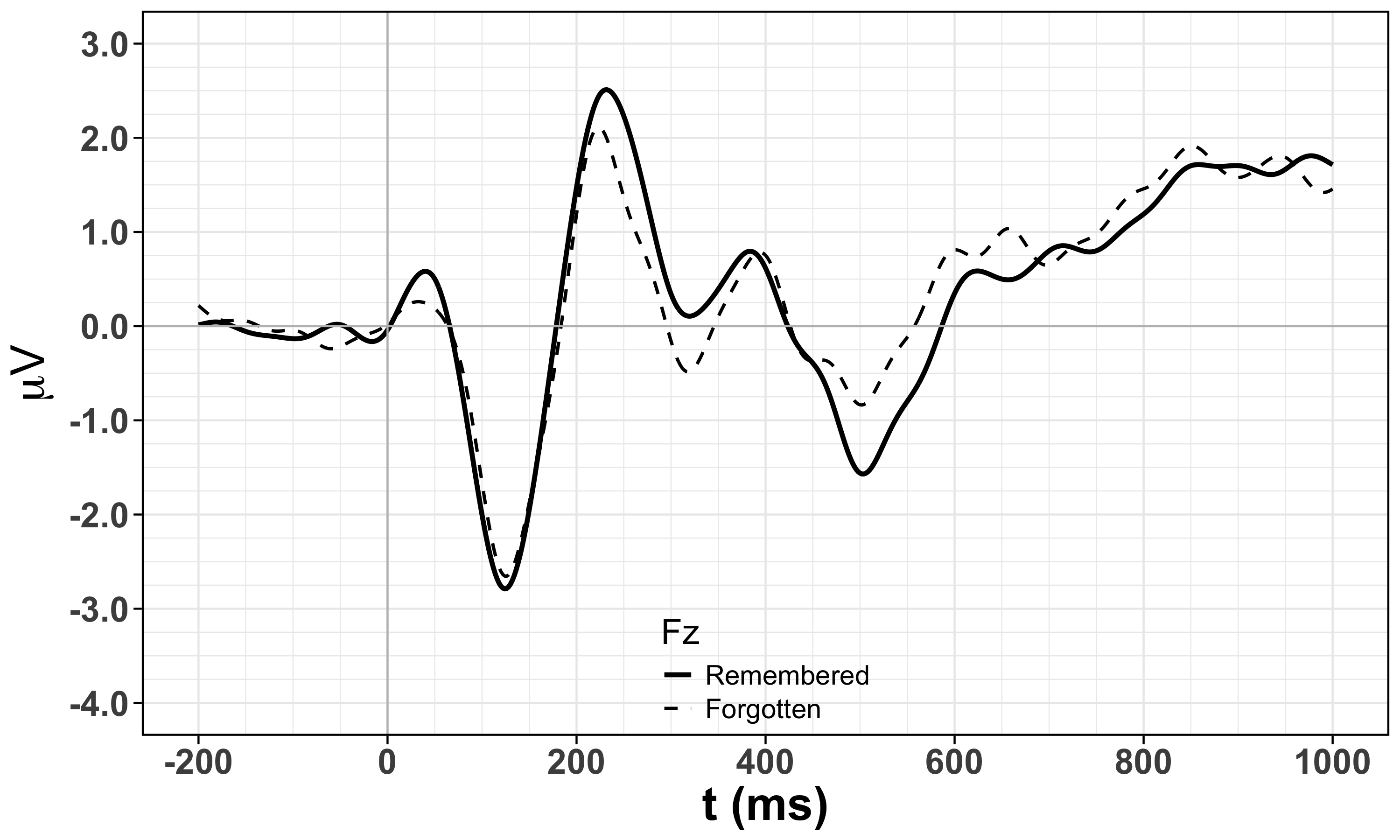

Supplement: Supplementary file 1 [file nutrients-17-00745-s001.zip › figures/fnam_name_imm_Fz.png]

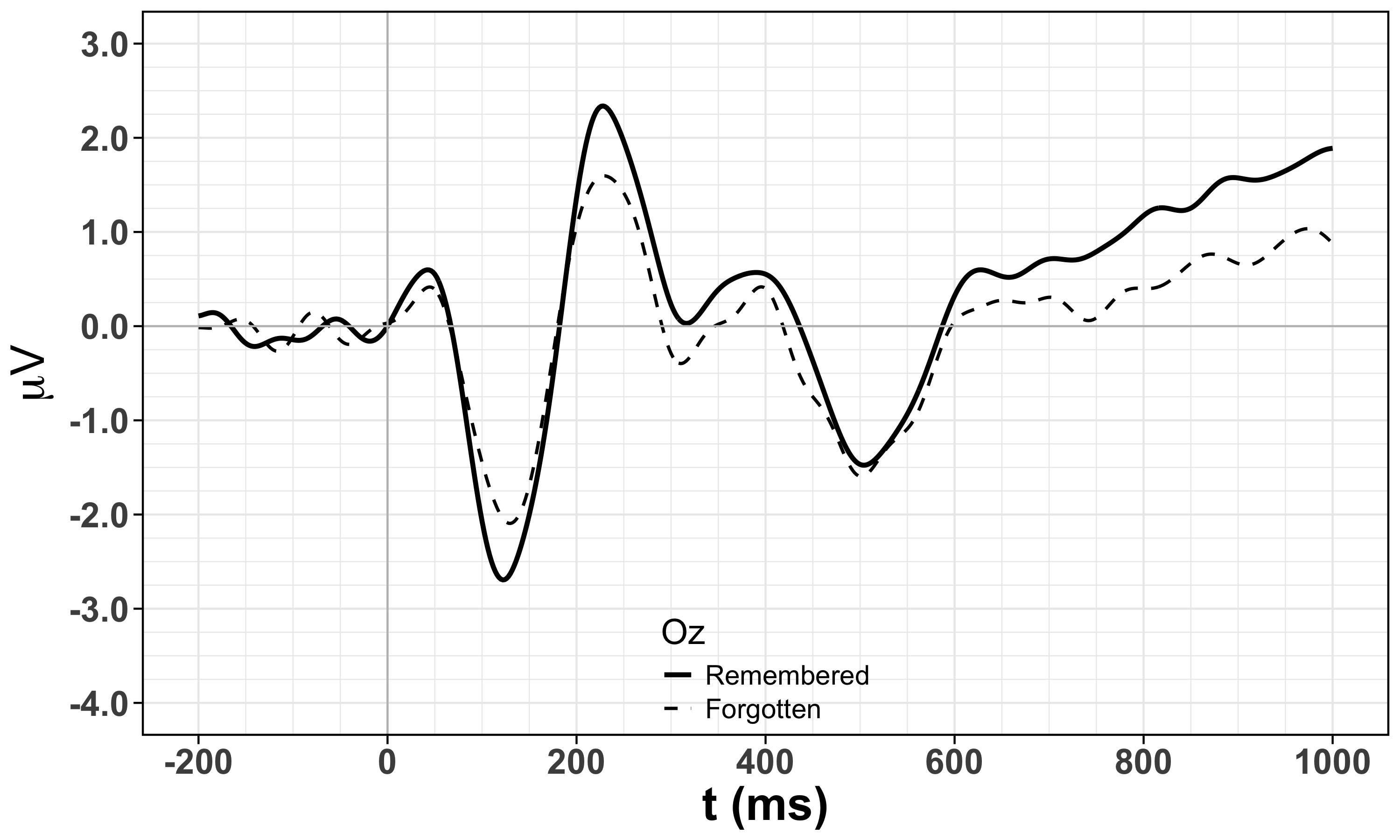

Supplement: Supplementary file 1 [file nutrients-17-00745-s001.zip › figures/fnam_name_imm_Oz.png]

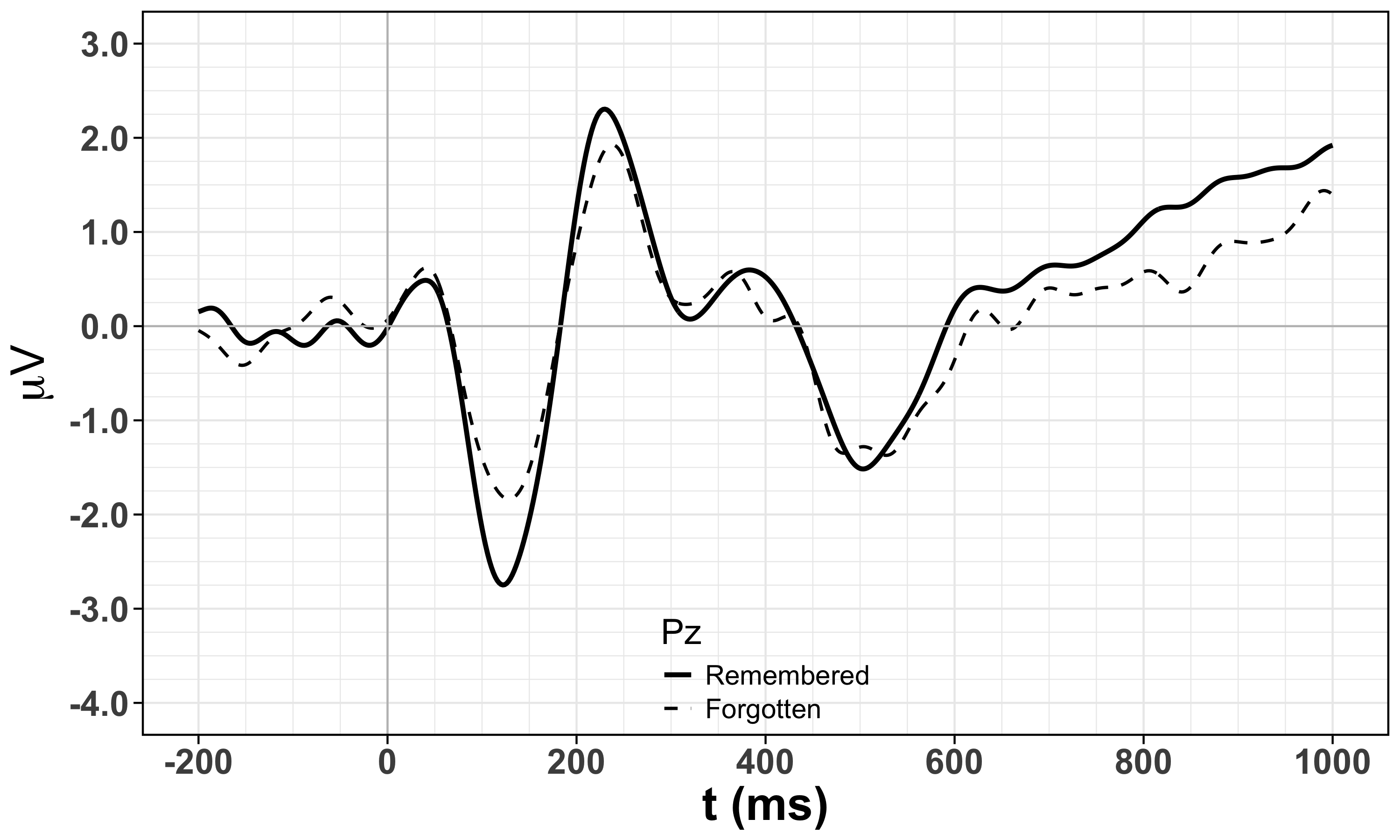

Supplement: Supplementary file 1 [file nutrients-17-00745-s001.zip › figures/fnam_name_imm_Pz.png]

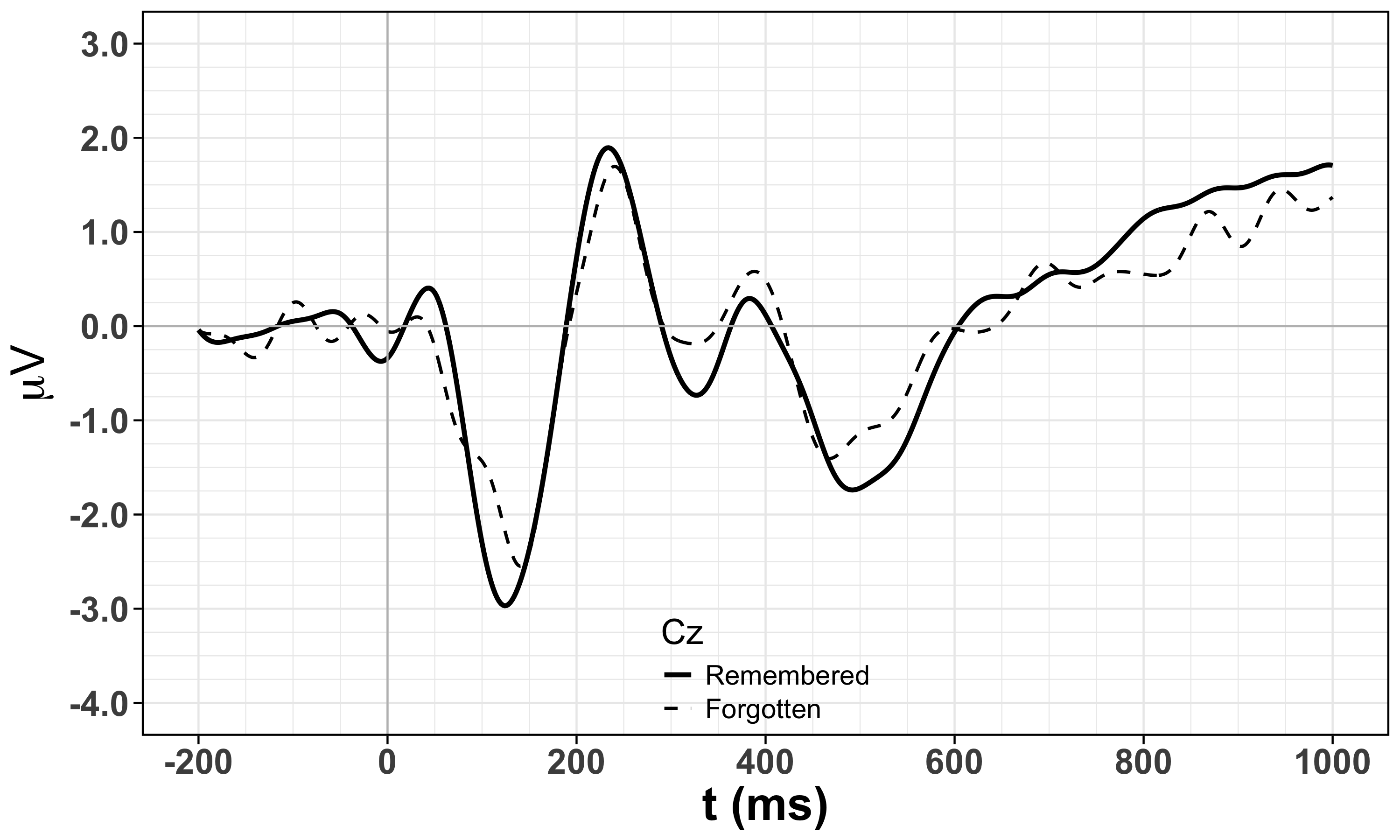

Supplement: Supplementary file 1 [file nutrients-17-00745-s001.zip › figures/fnam_occn_del_Cz.png]

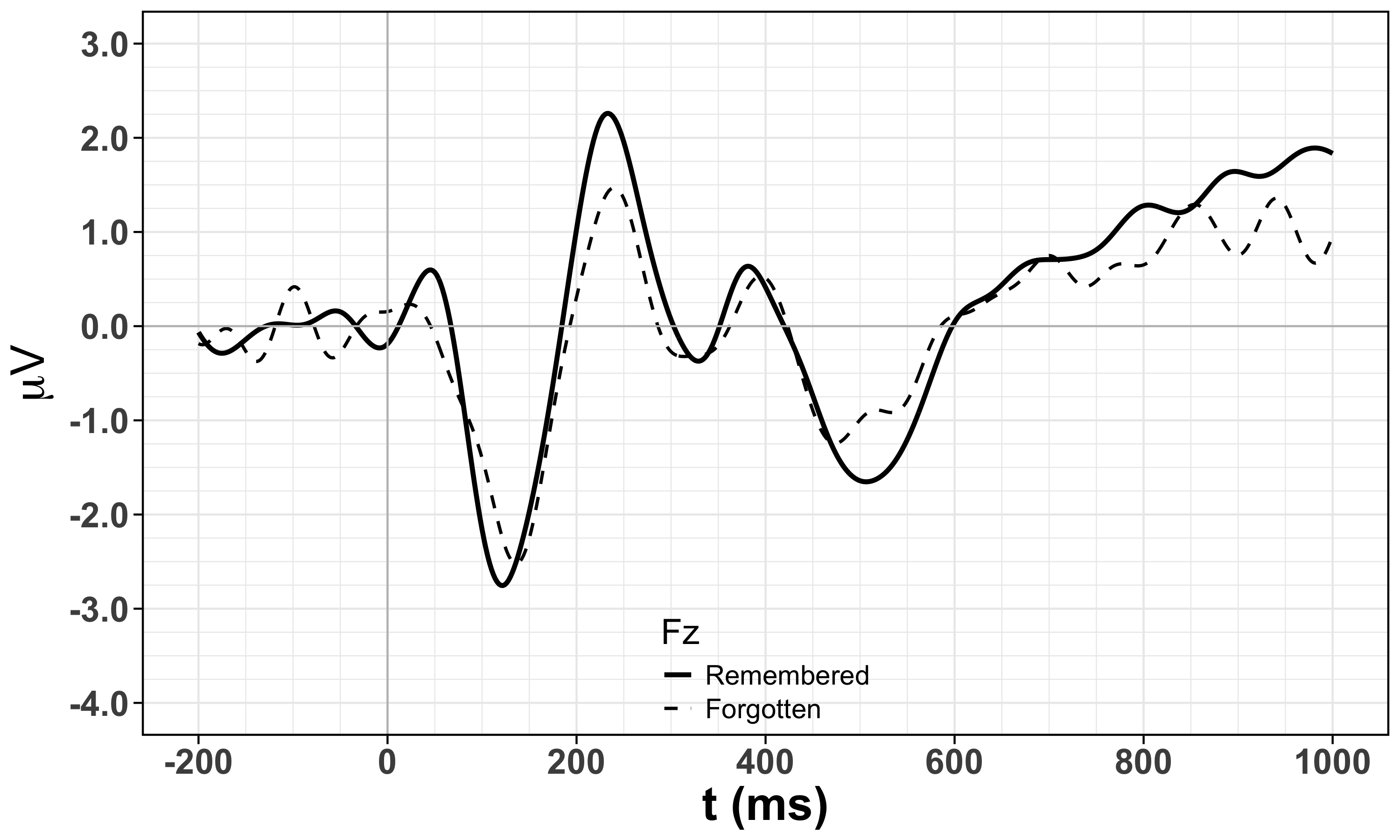

Supplement: Supplementary file 1 [file nutrients-17-00745-s001.zip › figures/fnam_occn_del_Fz.png]

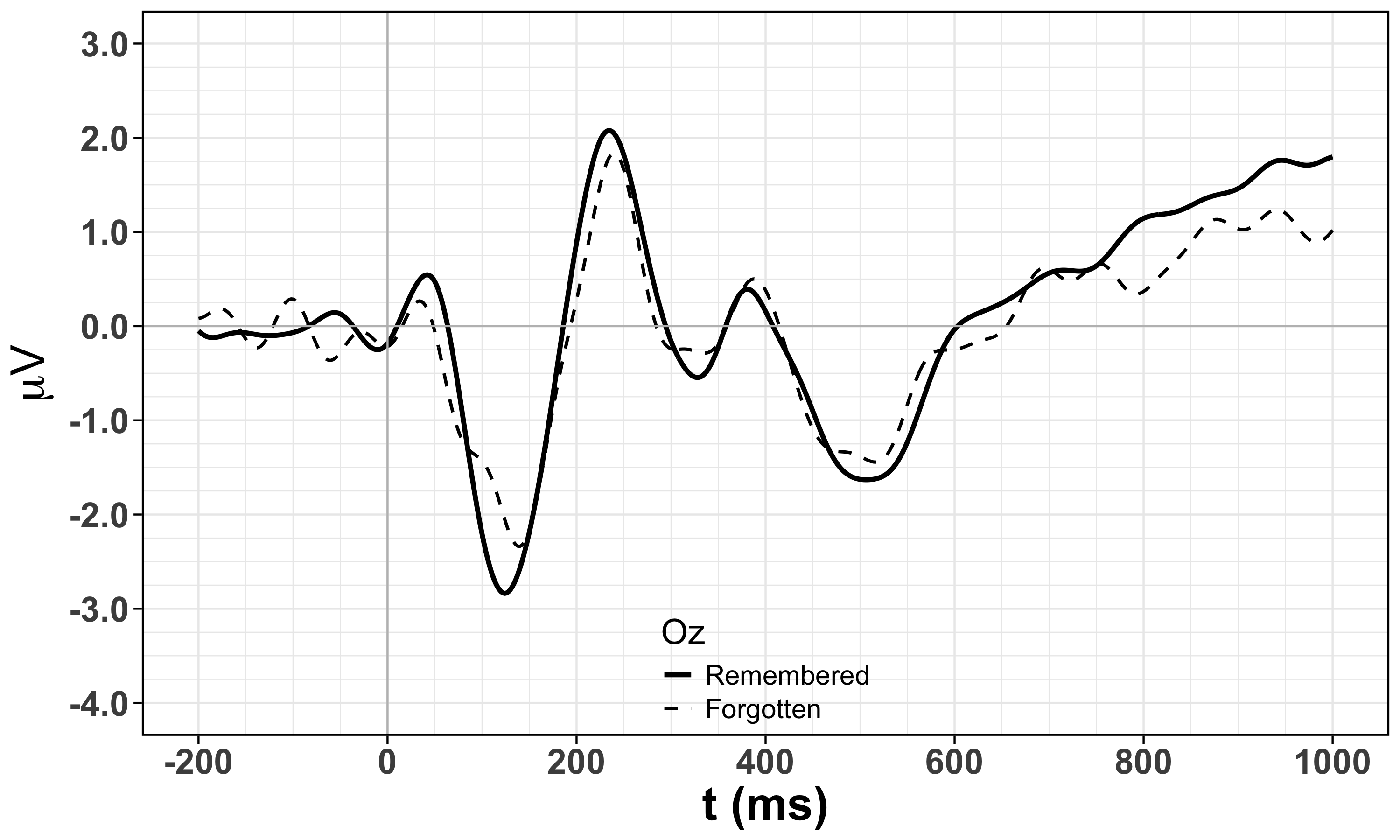

Supplement: Supplementary file 1 [file nutrients-17-00745-s001.zip › figures/fnam_occn_del_Oz.png]

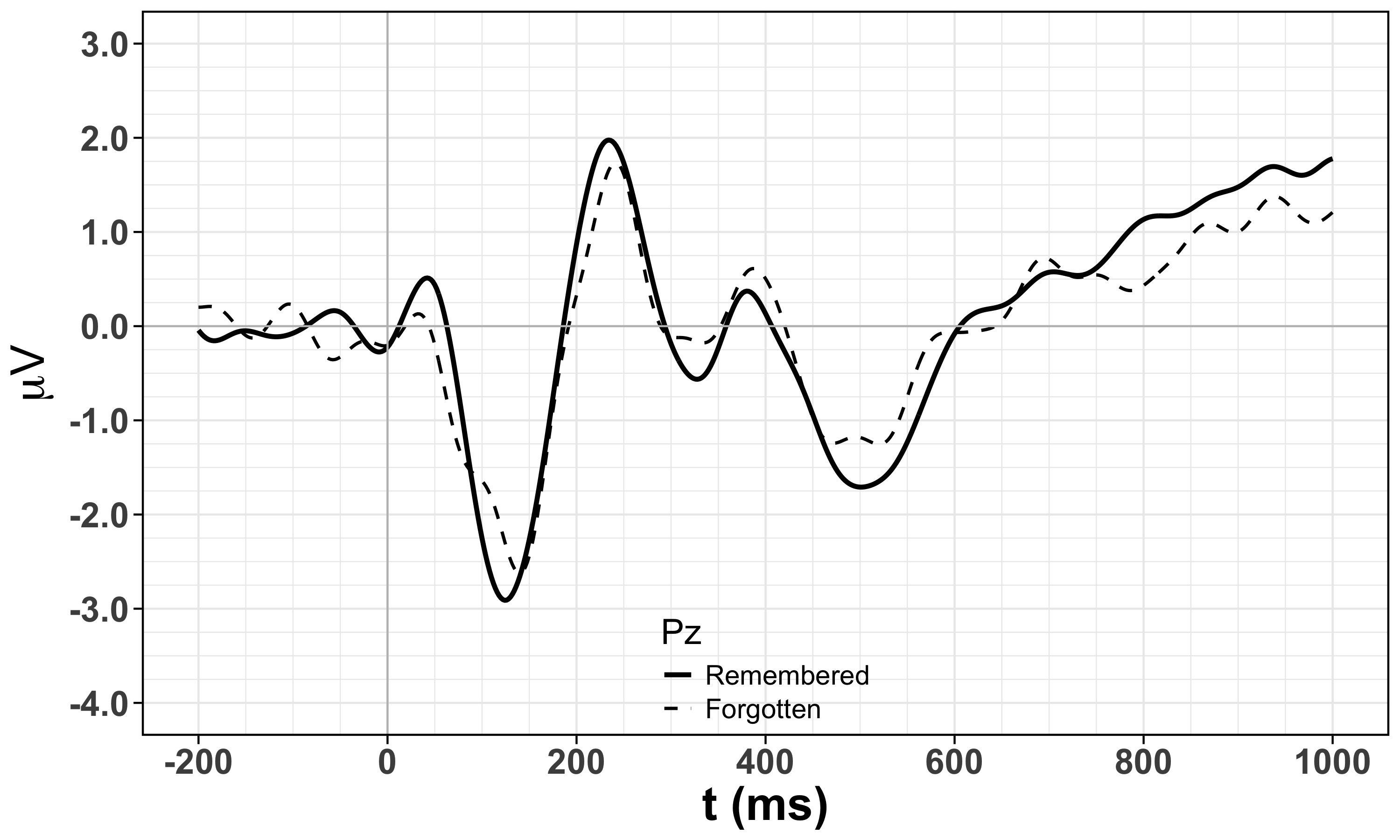

Supplement: Supplementary file 1 [file nutrients-17-00745-s001.zip › figures/fnam_occn_del_Pz.png]

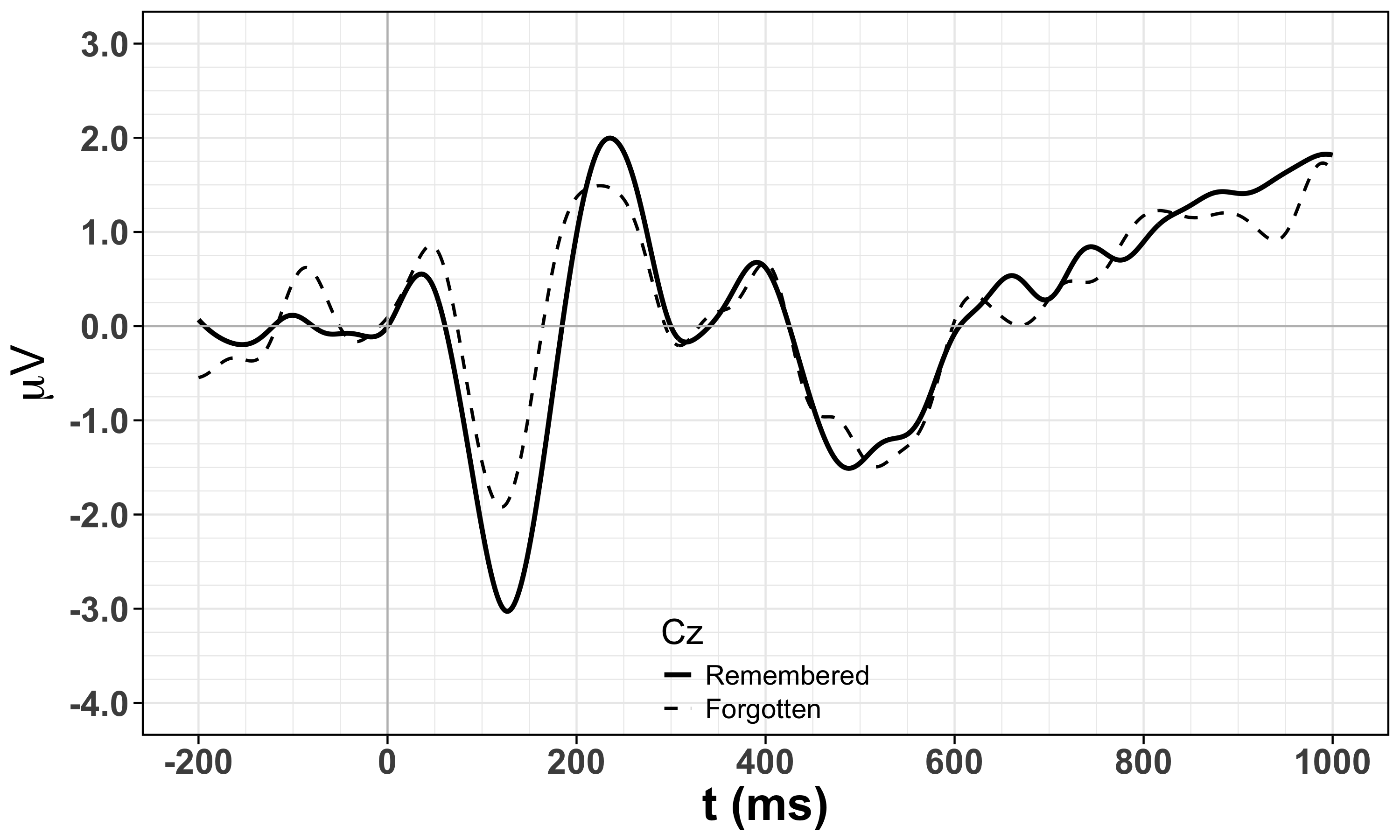

Supplement: Supplementary file 1 [file nutrients-17-00745-s001.zip › figures/fnam_occn_imm_Cz.png]

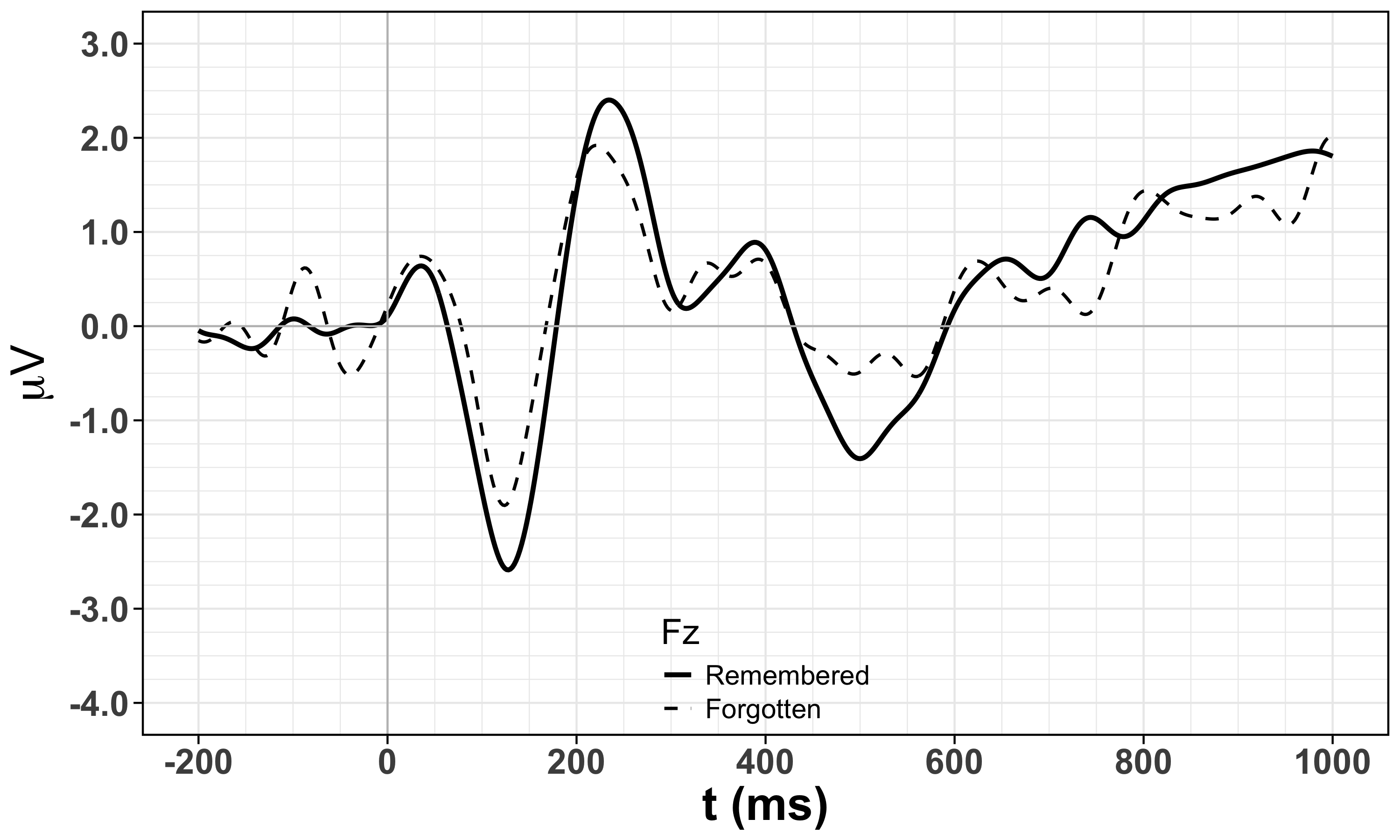

Supplement: Supplementary file 1 [file nutrients-17-00745-s001.zip › figures/fnam_occn_imm_Fz.png]

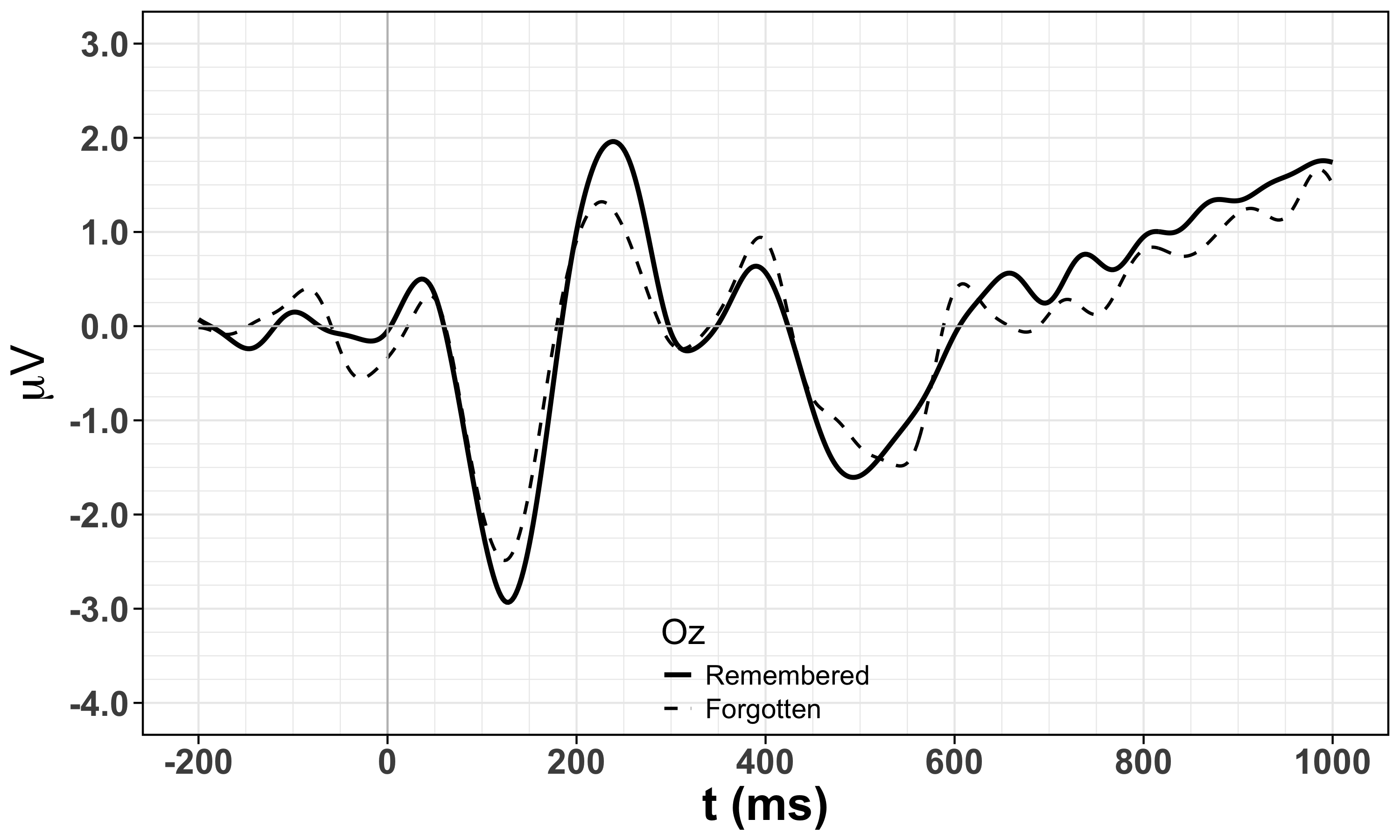

Supplement: Supplementary file 1 [file nutrients-17-00745-s001.zip › figures/fnam_occn_imm_Oz.png]

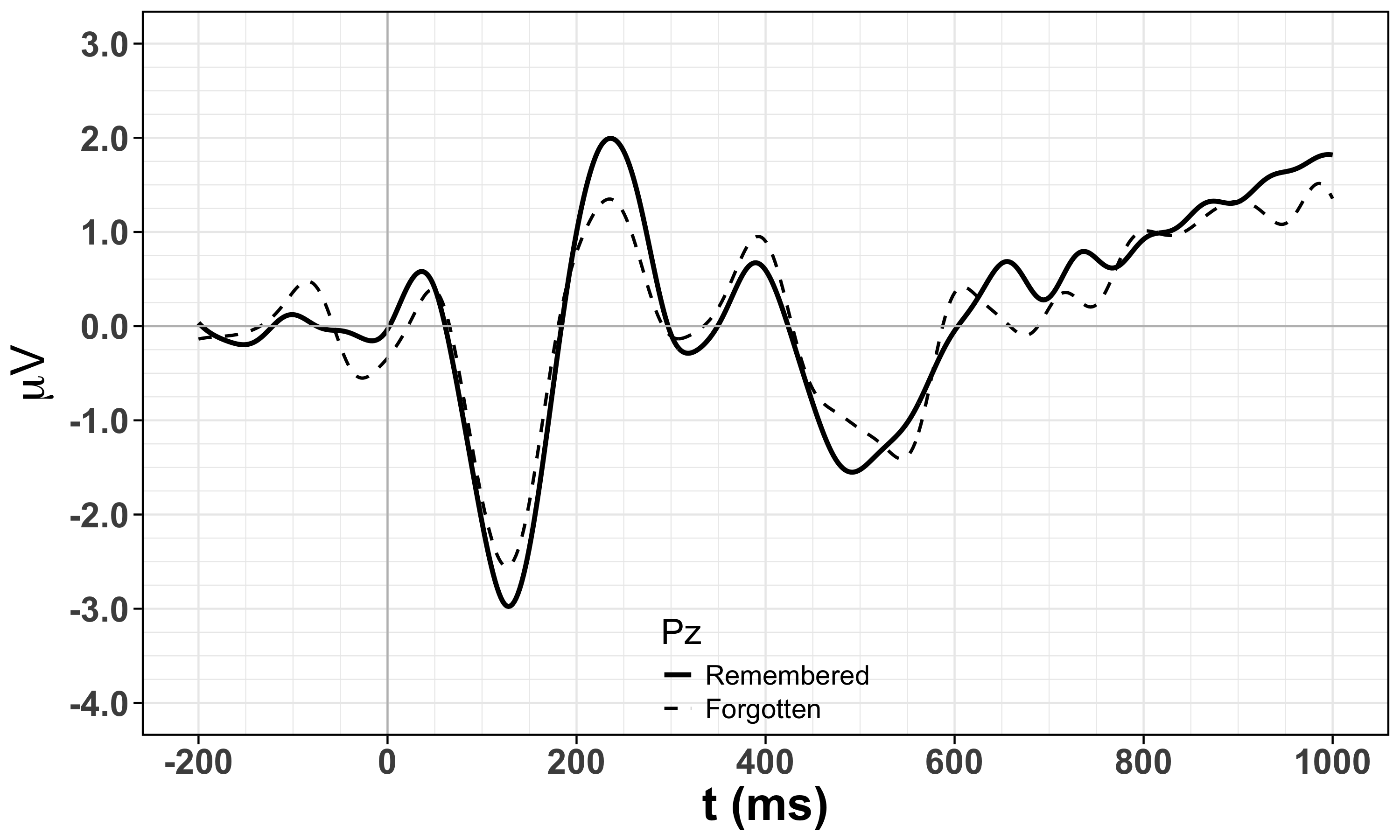

Supplement: Supplementary file 1 [file nutrients-17-00745-s001.zip › figures/fnam_occn_imm_Pz.png]

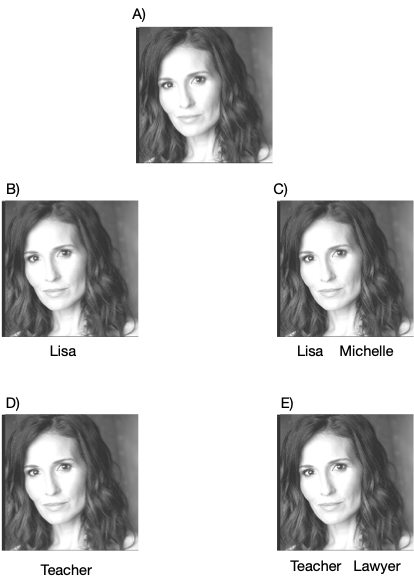

Supplement: Supplementary file 1 [file nutrients-17-00745-s001.zip › figures/fnam_stim.png]

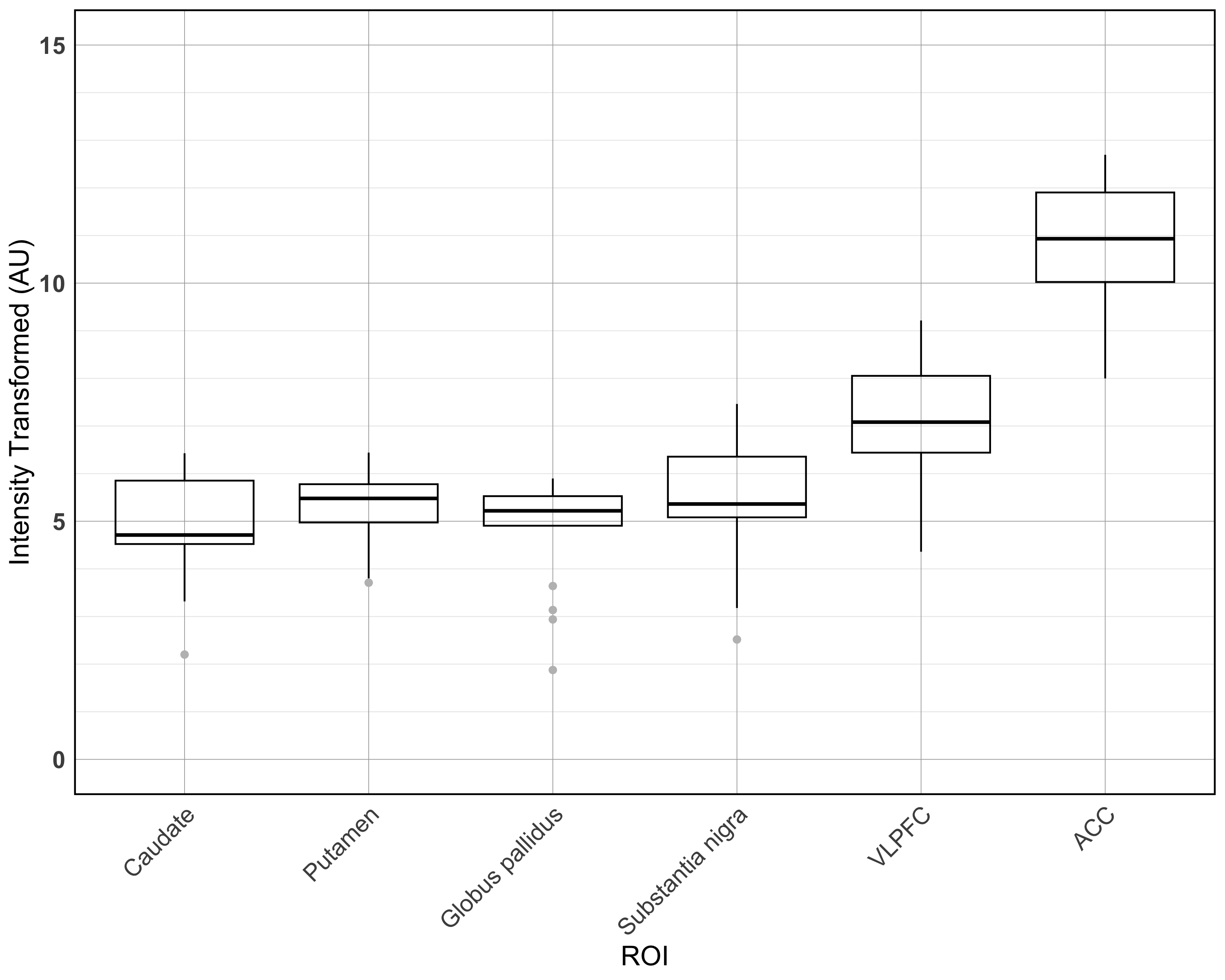

Supplement: Supplementary file 1 [file nutrients-17-00745-s001.zip › figures/left_transformed.png]

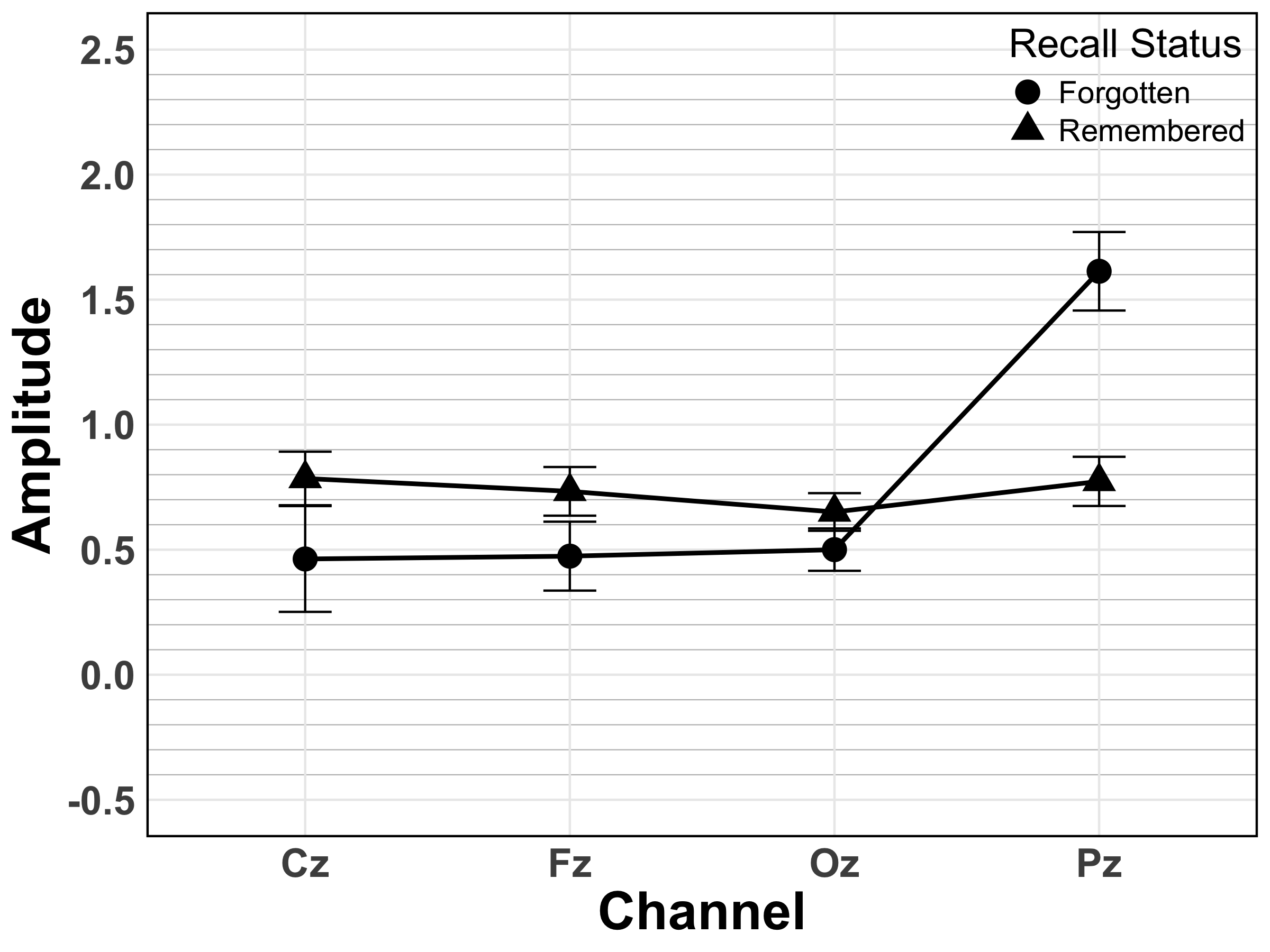

Supplement: Supplementary file 1 [file nutrients-17-00745-s001.zip › figures/means_fnd600.png]

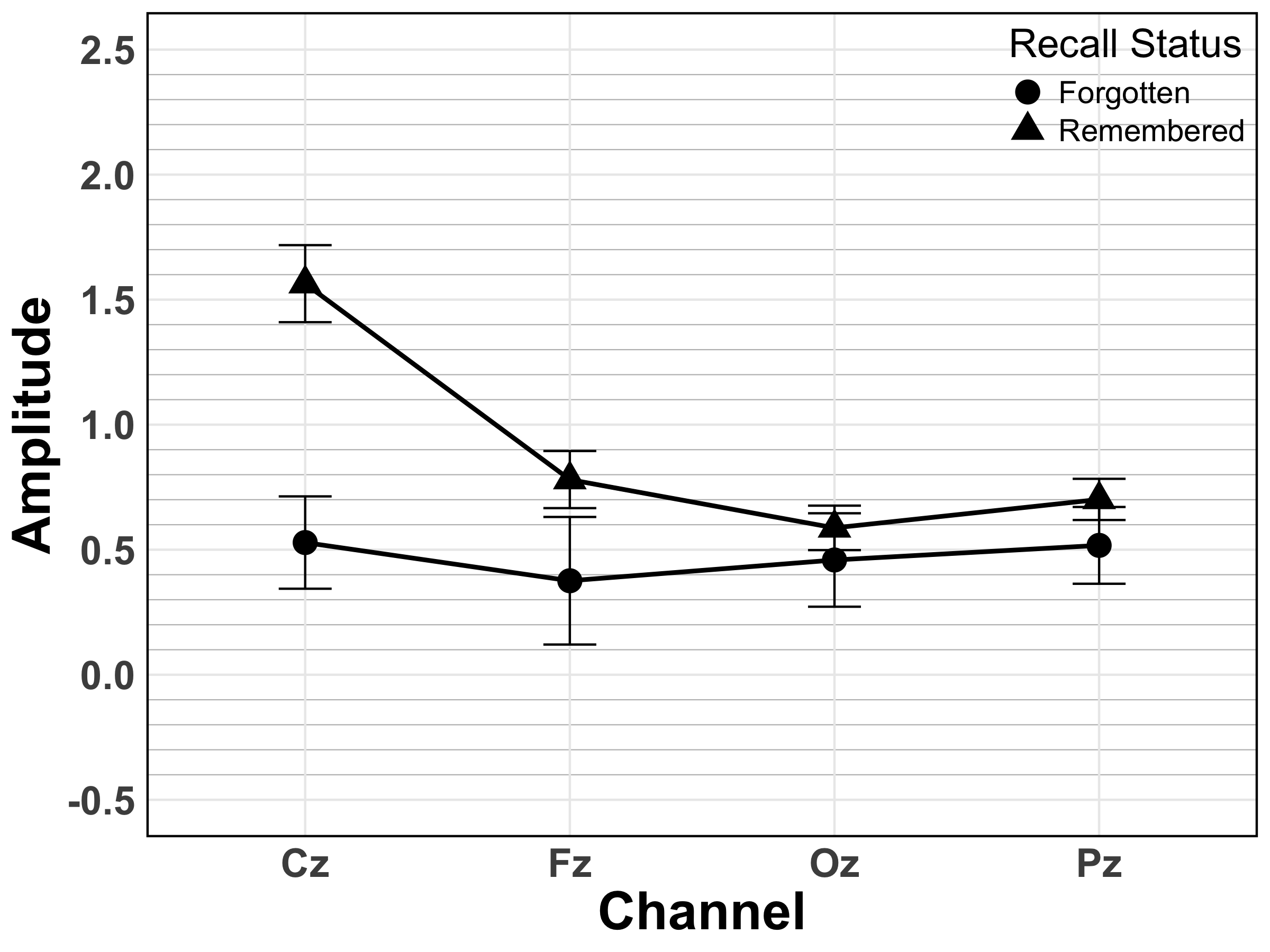

Supplement: Supplementary file 1 [file nutrients-17-00745-s001.zip › figures/means_fod200.png]

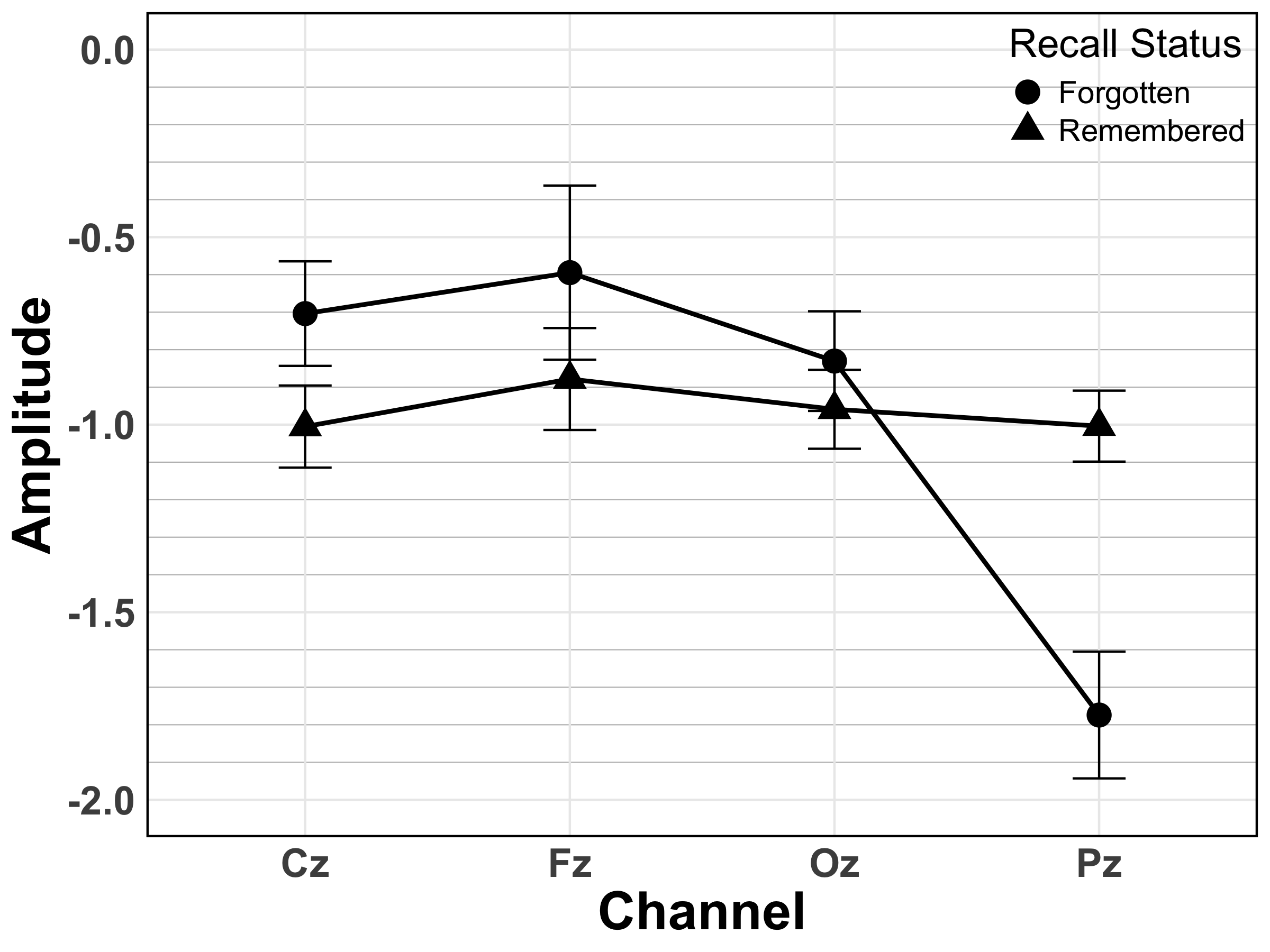

Supplement: Supplementary file 1 [file nutrients-17-00745-s001.zip › figures/means_fod400.png]

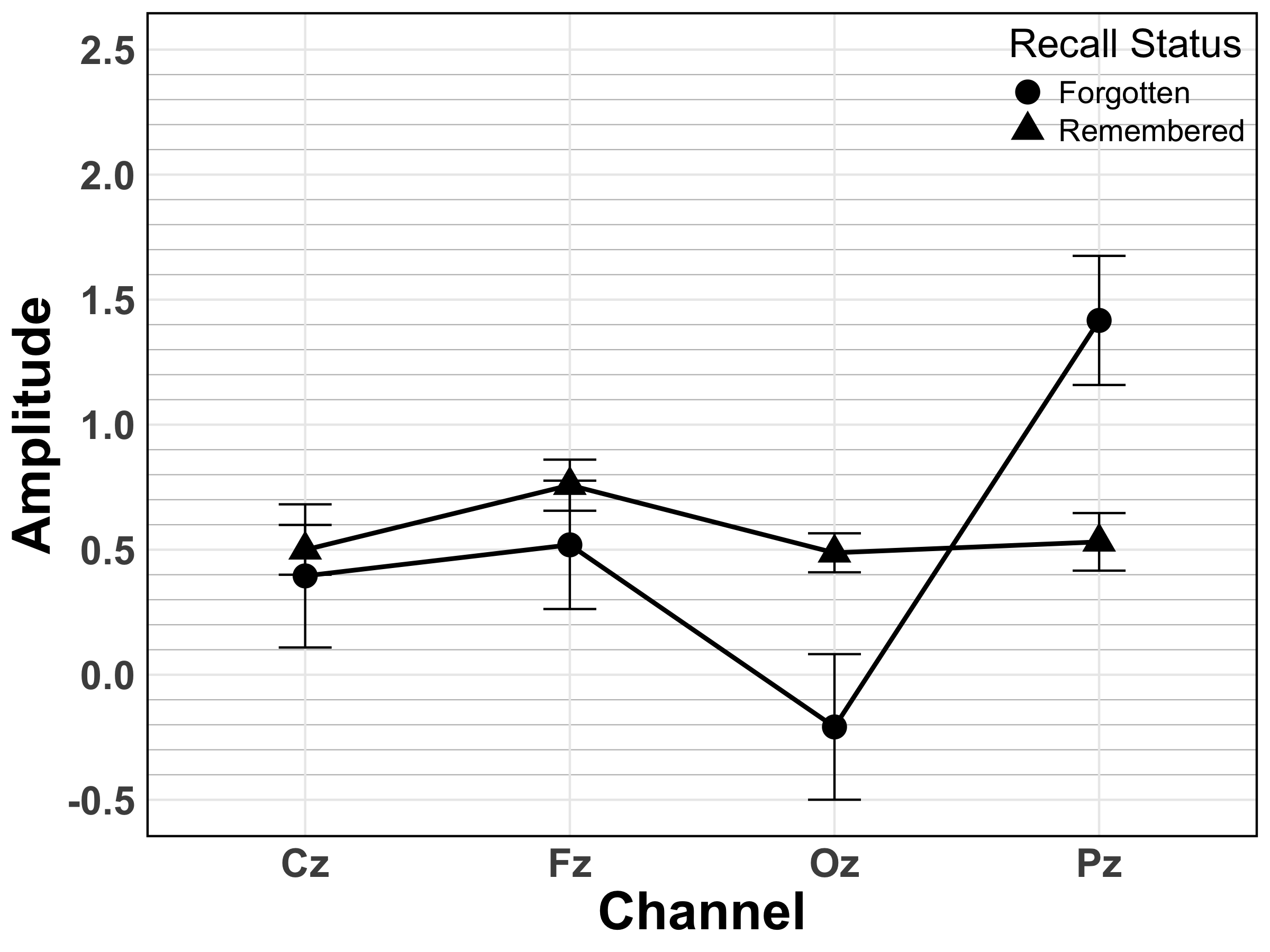

Supplement: Supplementary file 1 [file nutrients-17-00745-s001.zip › figures/means_foi600.png]

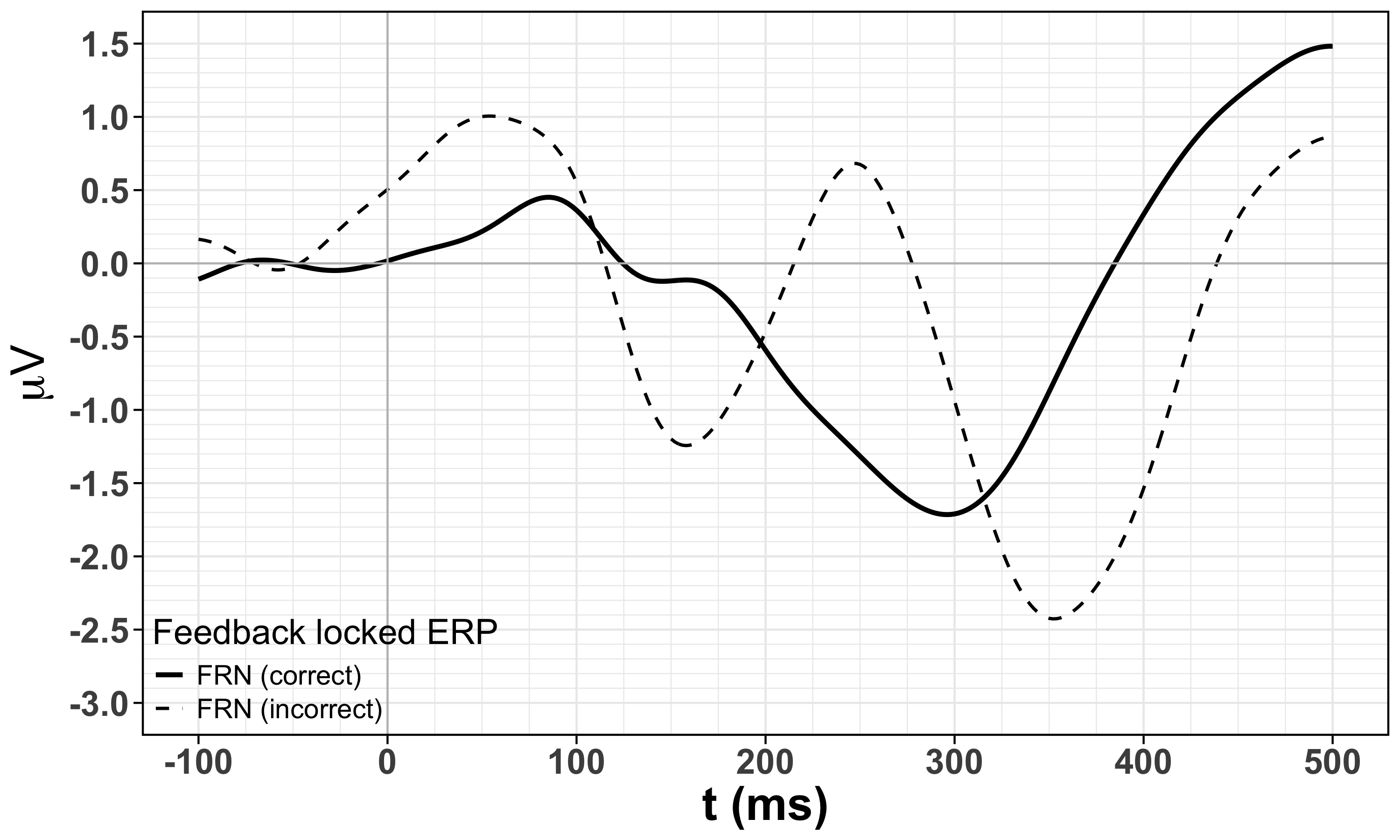

Supplement: Supplementary file 1 [file nutrients-17-00745-s001.zip › figures/pst_fl_erp.png]

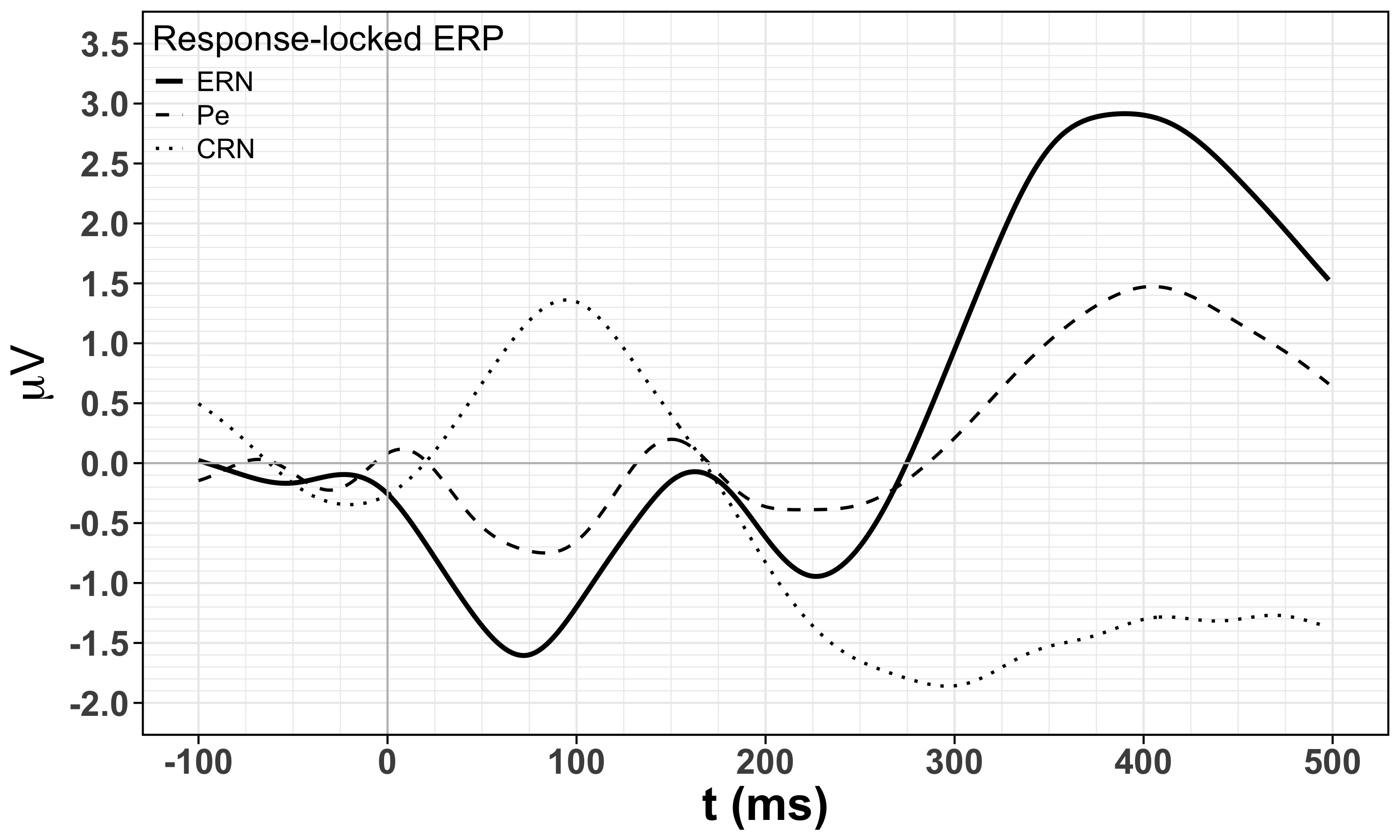

Supplement: Supplementary file 1 [file nutrients-17-00745-s001.zip › figures/pst_rl_erp.png]

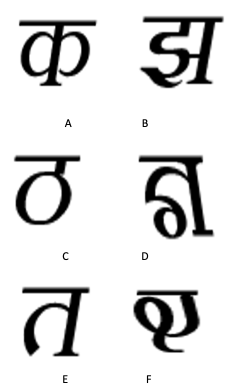

Supplement: Supplementary file 1 [file nutrients-17-00745-s001.zip › figures/pst_stim.png]

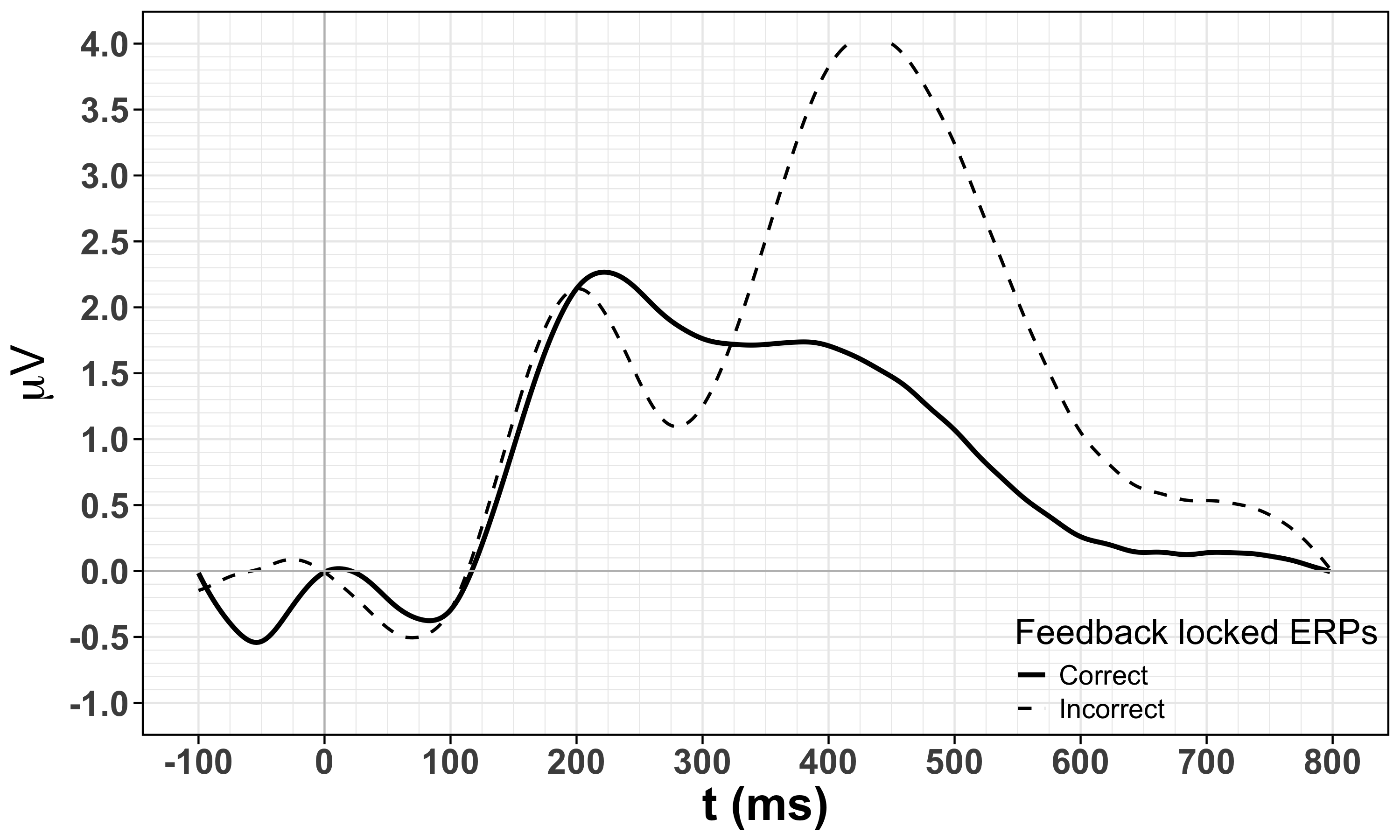

Supplement: Supplementary file 1 [file nutrients-17-00745-s001.zip › figures/rbcl_fl_erp.png]

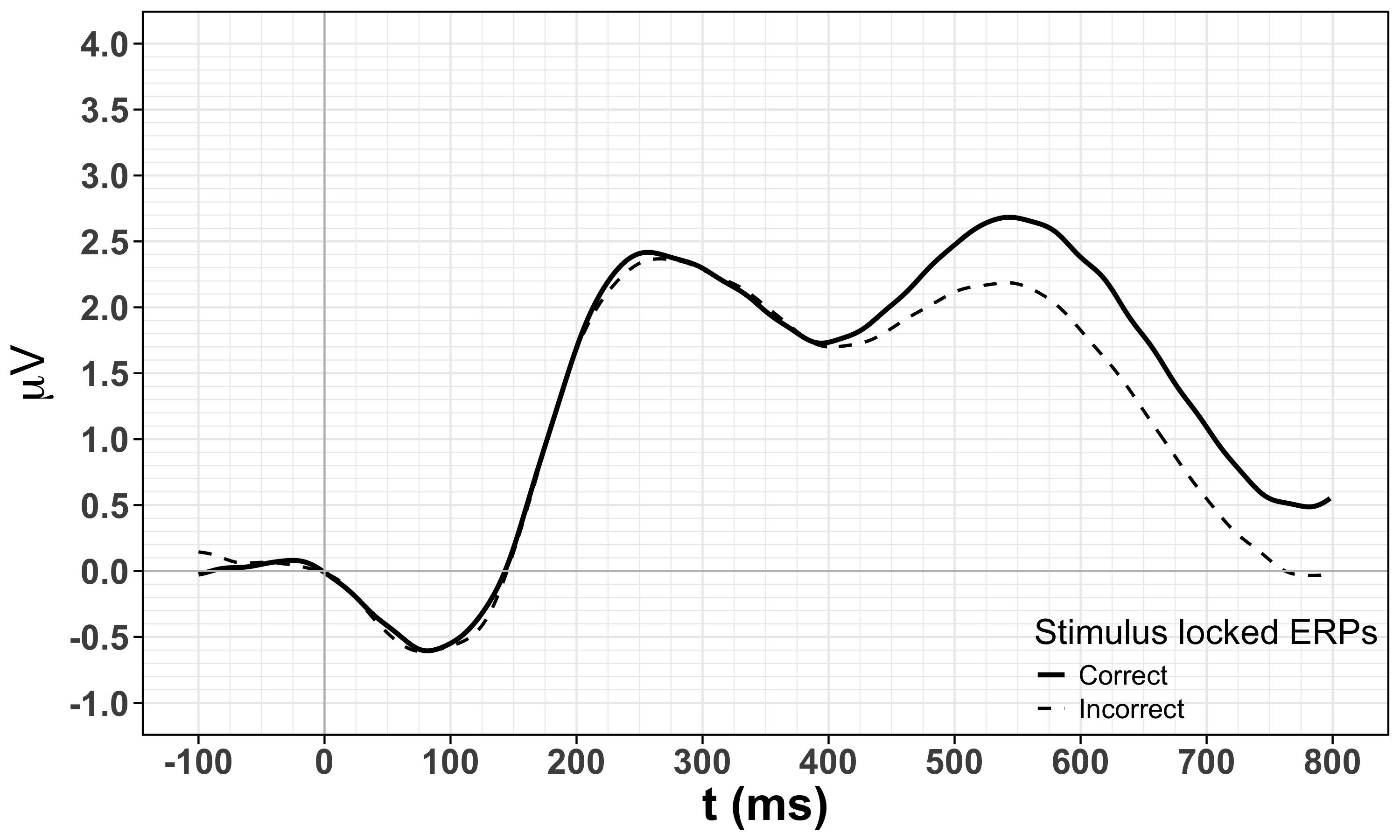

Supplement: Supplementary file 1 [file nutrients-17-00745-s001.zip › figures/rbcl_sl_erp.png]

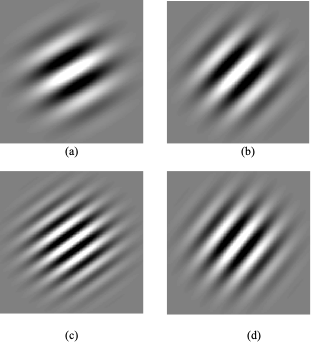

Supplement: Supplementary file 1 [file nutrients-17-00745-s001.zip › figures/rbcl_stim.png]

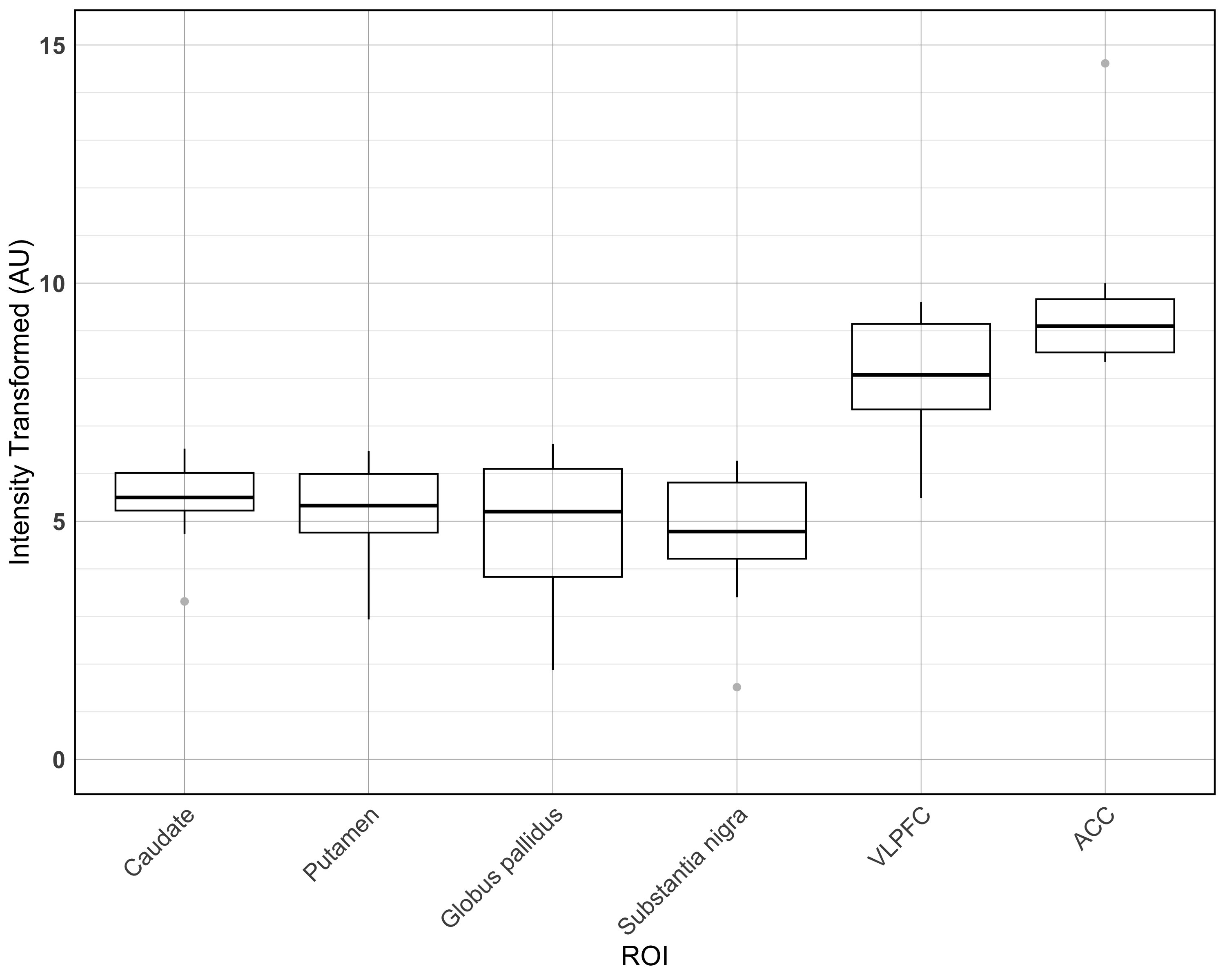

Supplement: Supplementary file 1 [file nutrients-17-00745-s001.zip › figures/right_transformed.png]

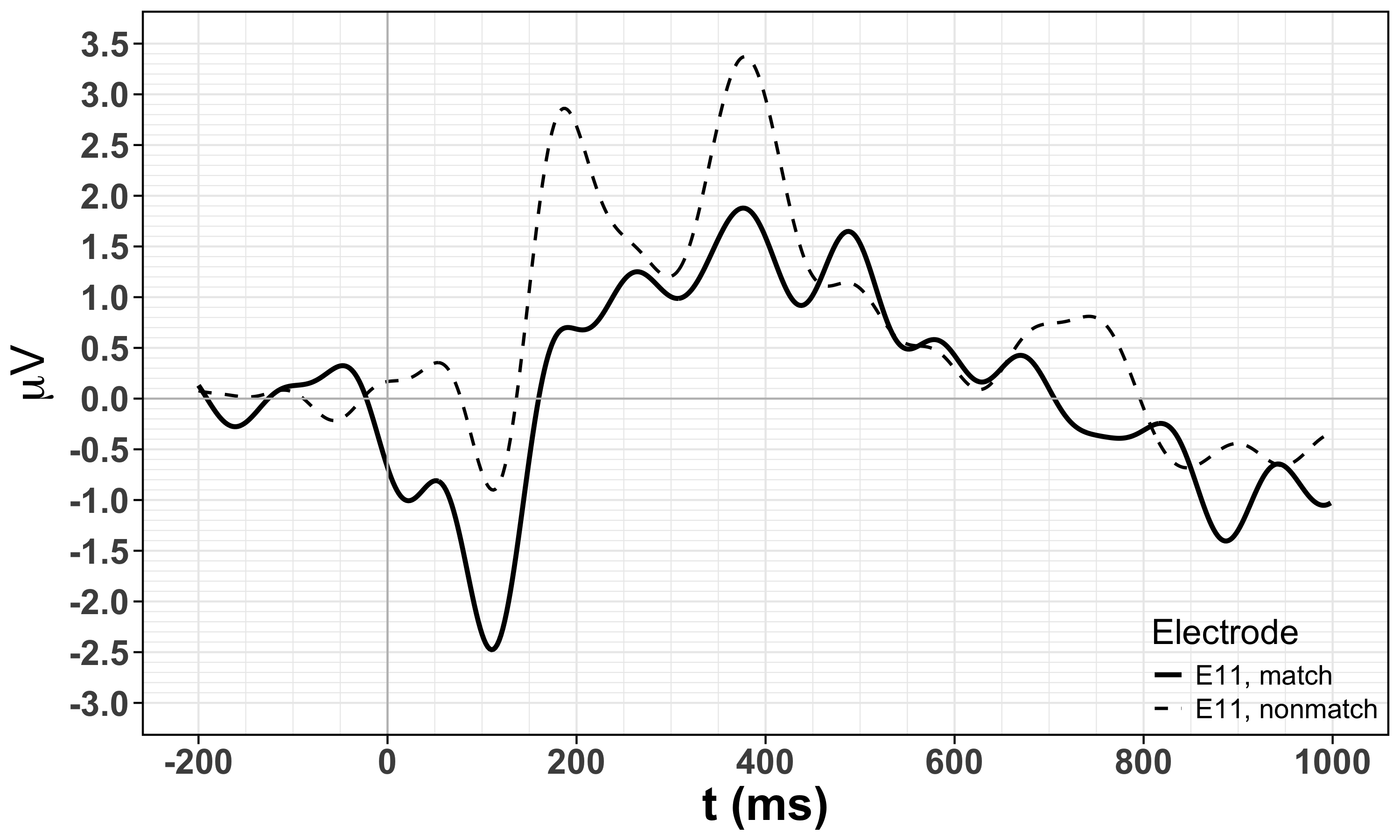

Supplement: Supplementary file 1 [file nutrients-17-00745-s001.zip › figures/vswm_e11_3-0.png]

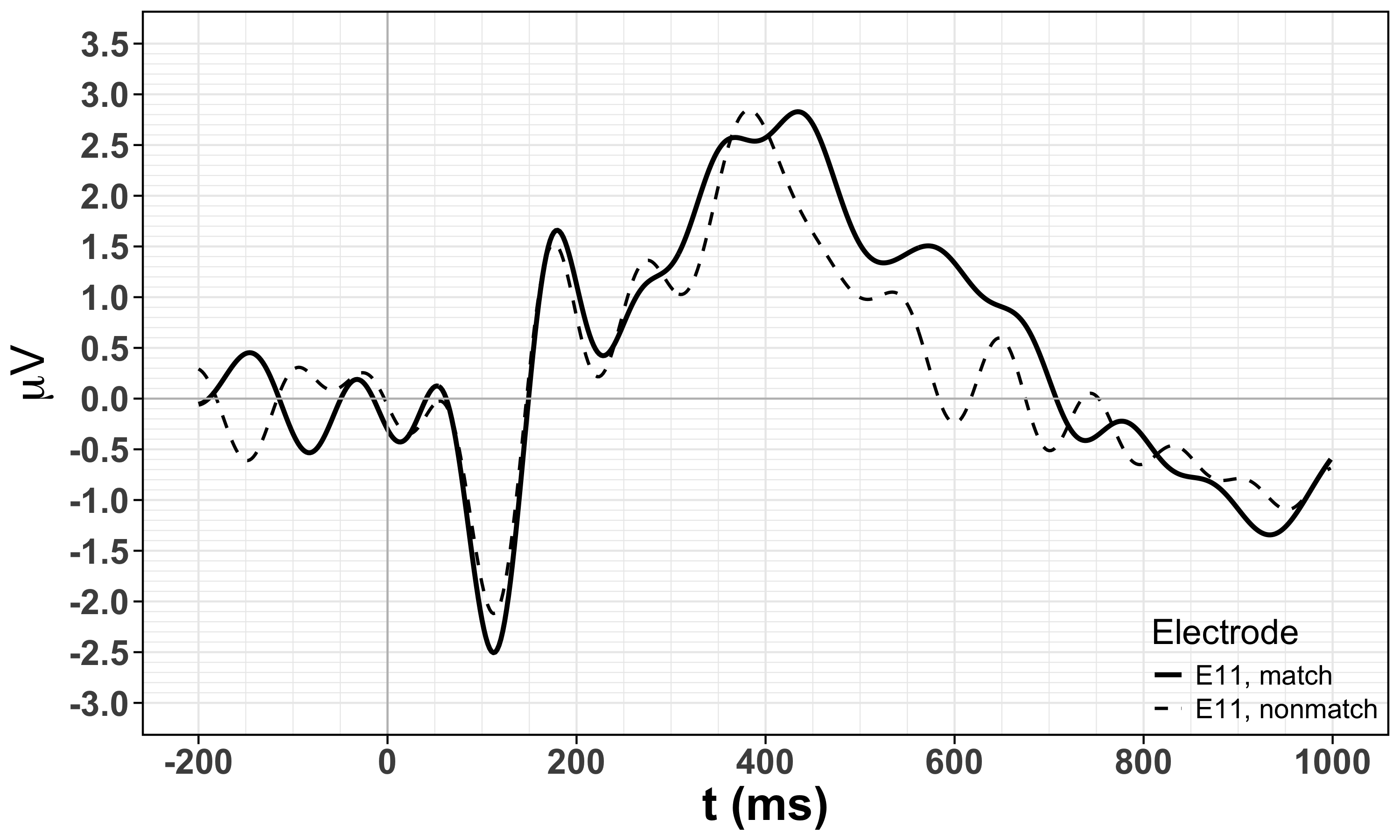

Supplement: Supplementary file 1 [file nutrients-17-00745-s001.zip › figures/vswm_e11_3-2.png]

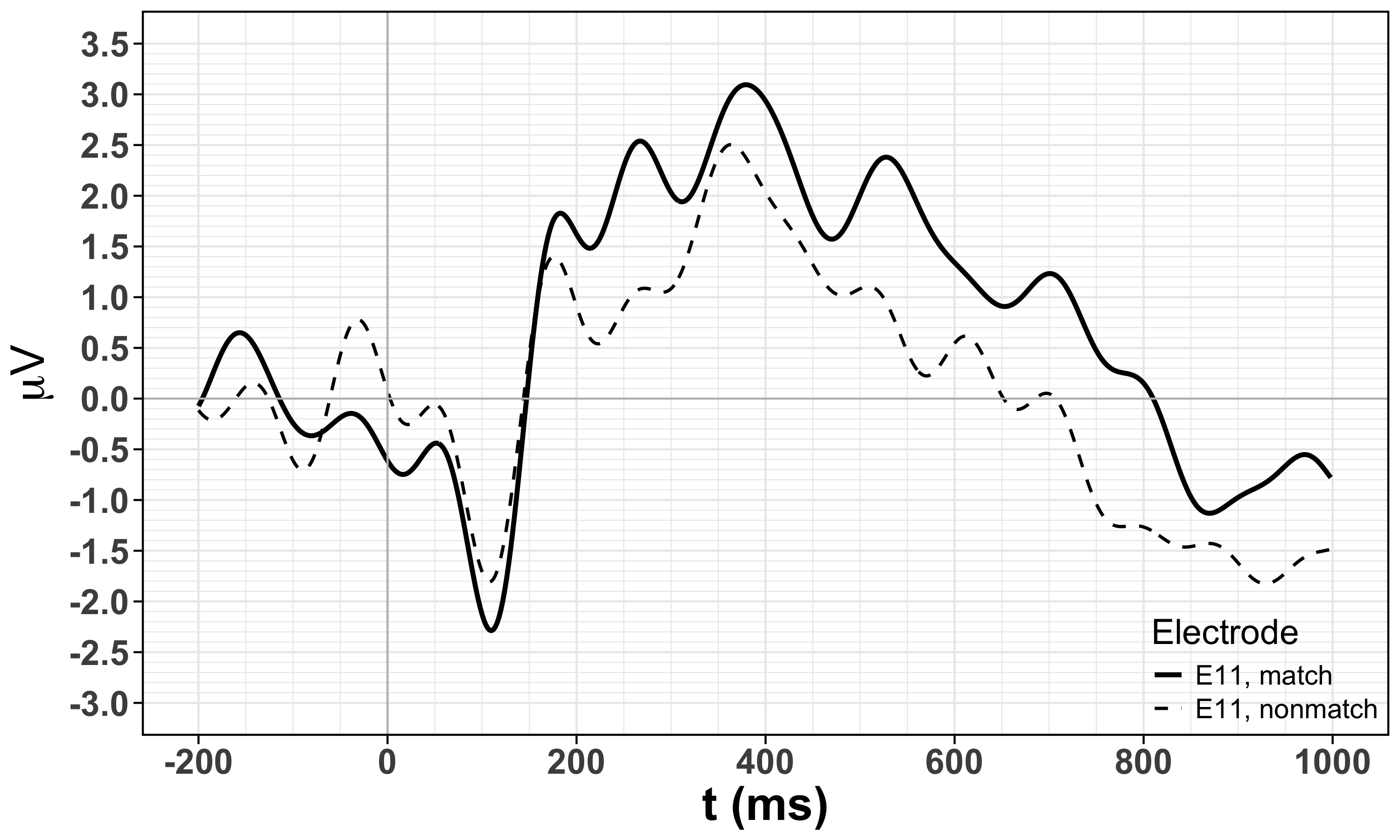

Supplement: Supplementary file 1 [file nutrients-17-00745-s001.zip › figures/vswm_e11_5-0.png]

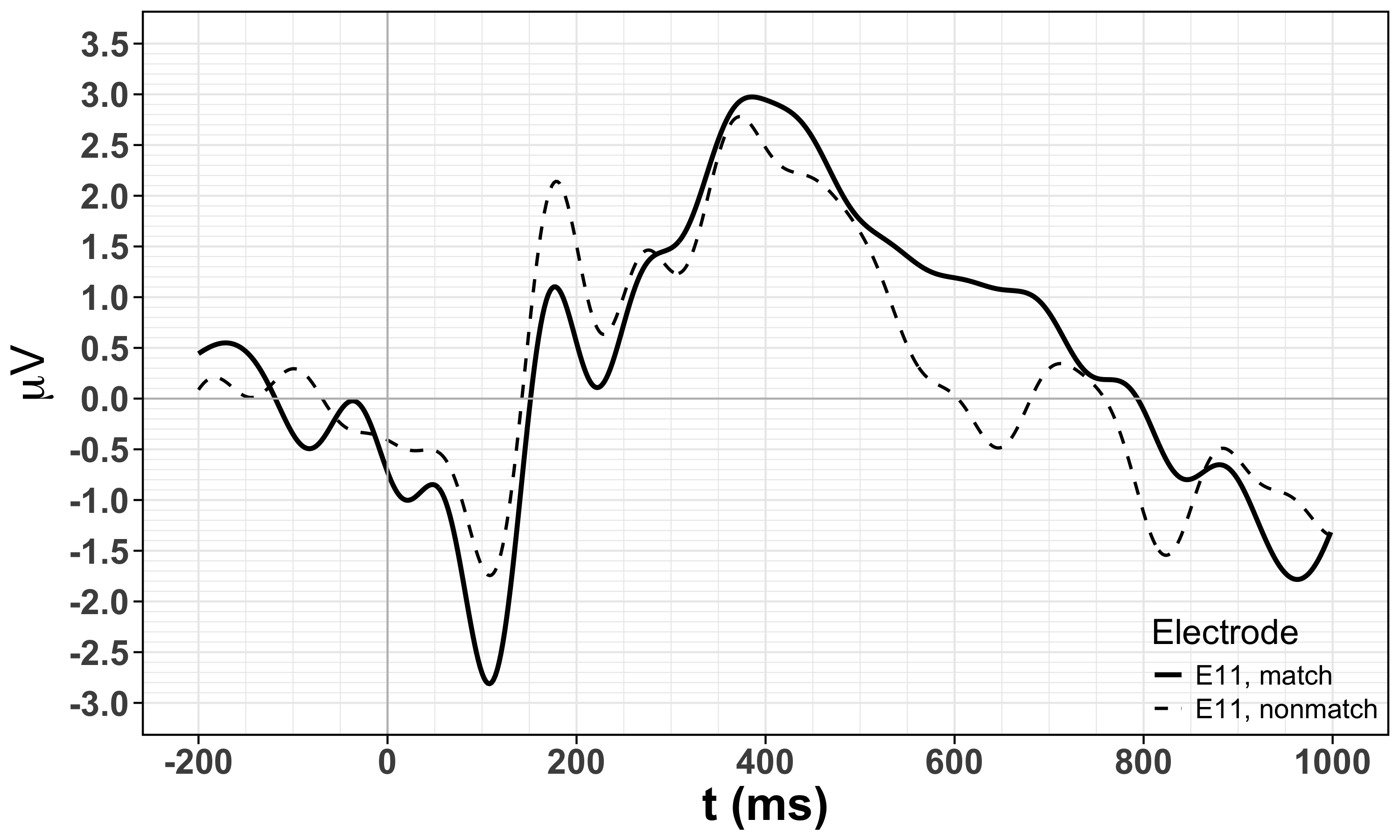

Supplement: Supplementary file 1 [file nutrients-17-00745-s001.zip › figures/vswm_e11_5-2.png]

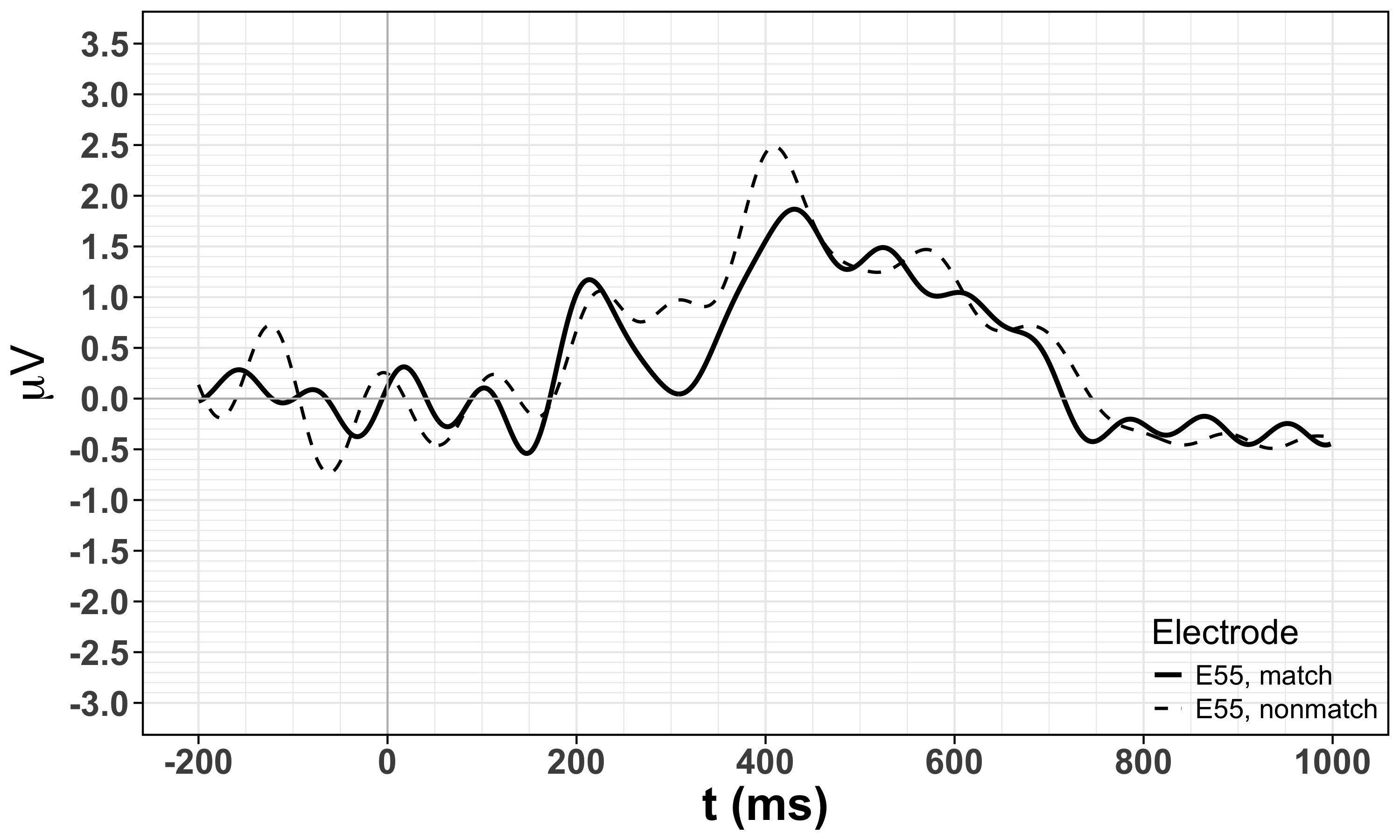

Supplement: Supplementary file 1 [file nutrients-17-00745-s001.zip › figures/vswm_e55_3-0.png]

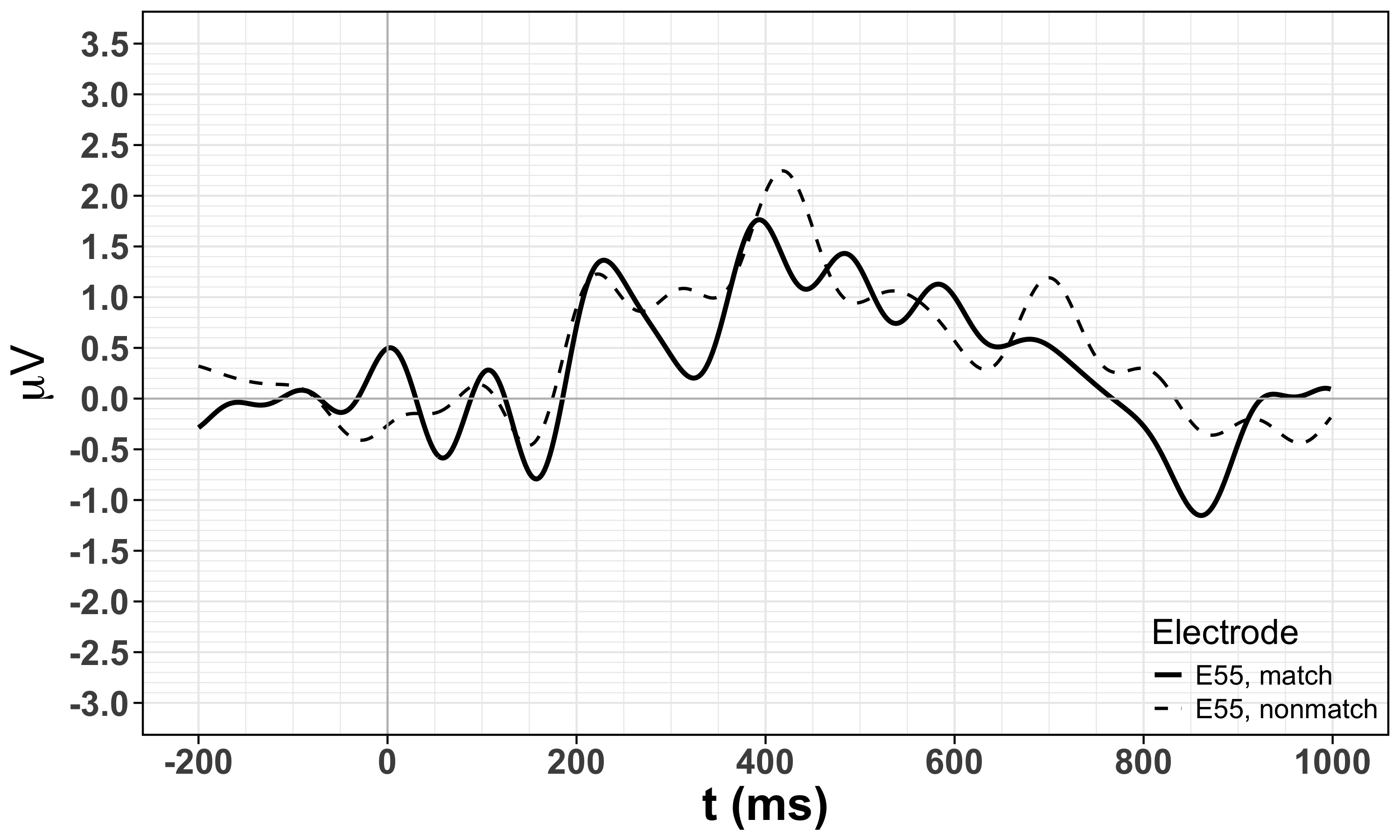

Supplement: Supplementary file 1 [file nutrients-17-00745-s001.zip › figures/vswm_e55_3-2.png]

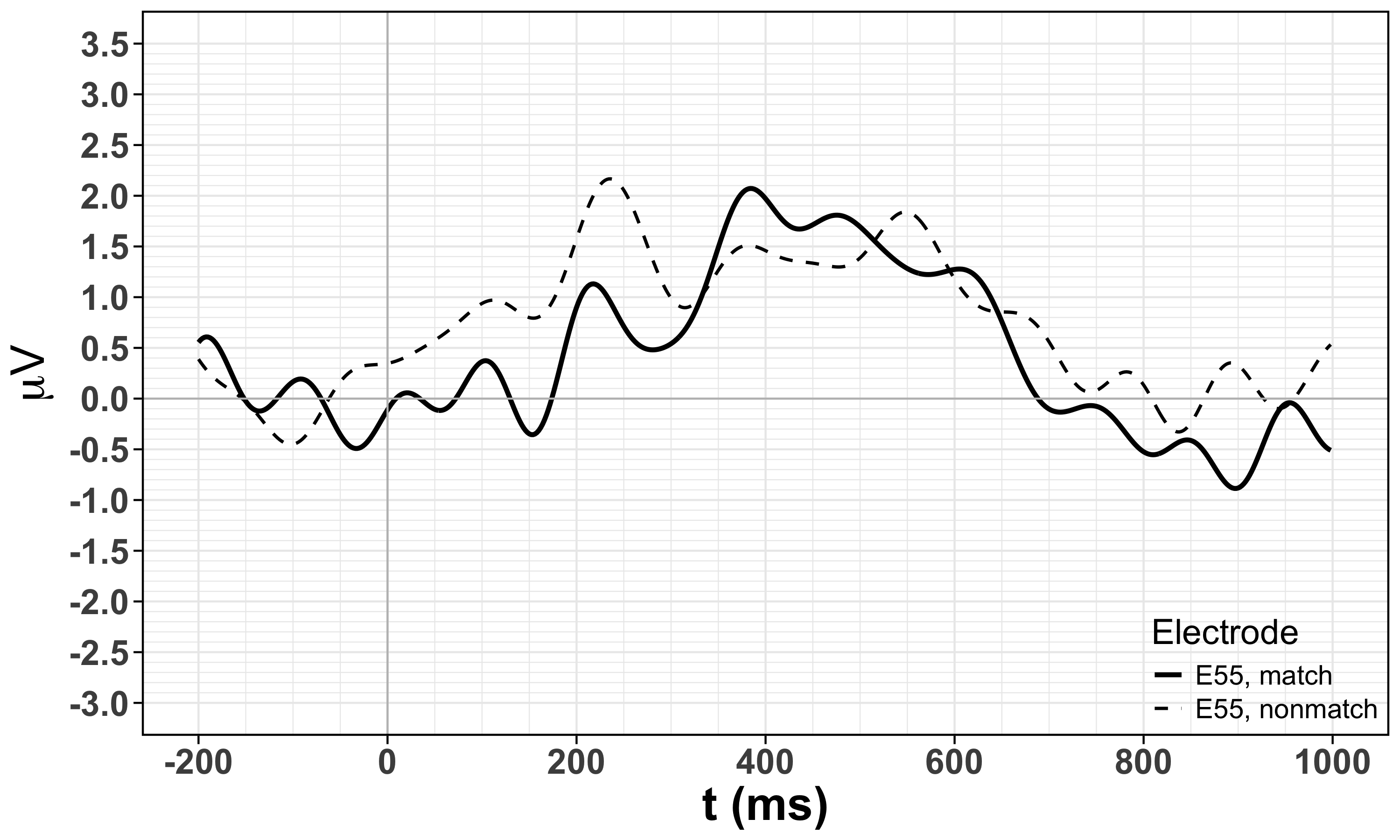

Supplement: Supplementary file 1 [file nutrients-17-00745-s001.zip › figures/vswm_e55_5-0.png]

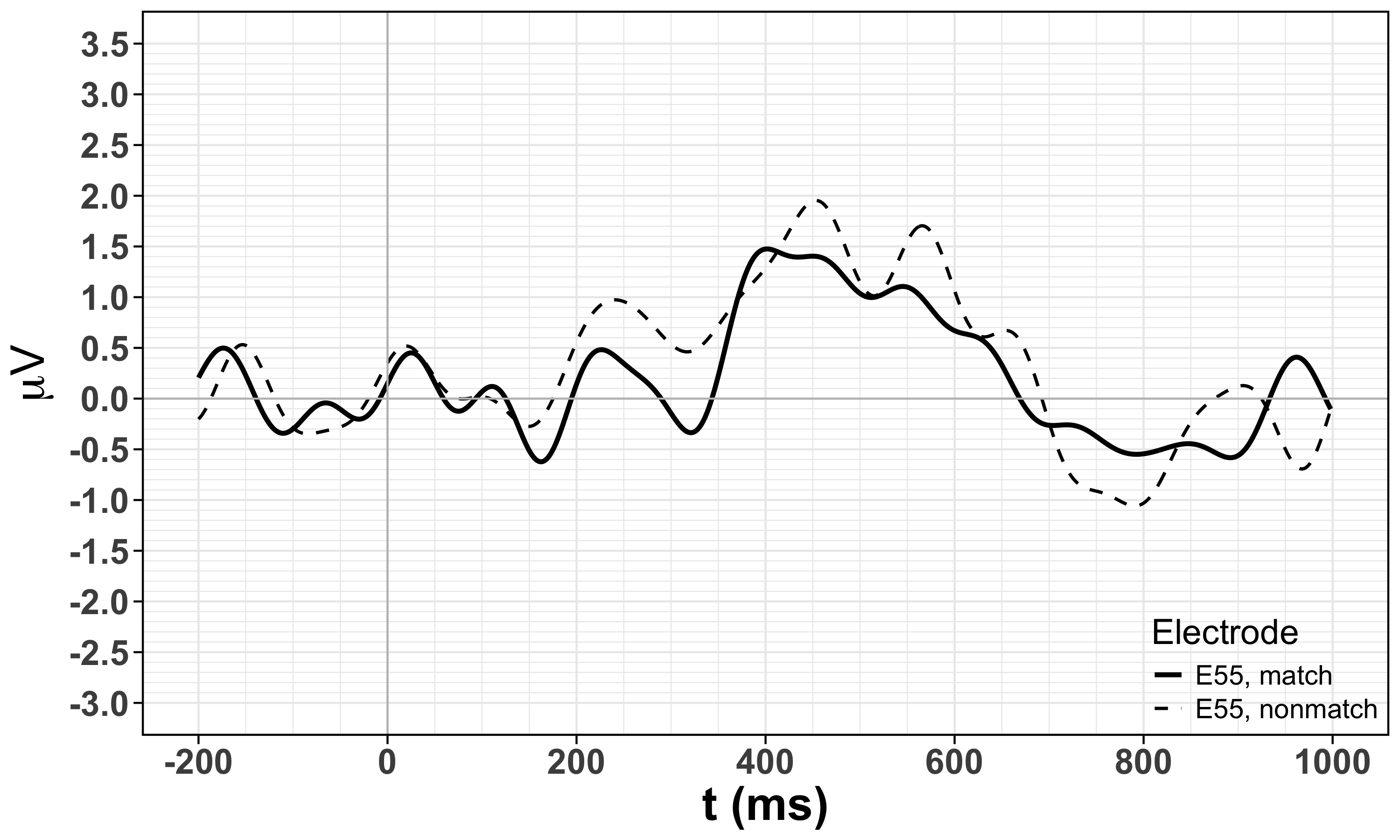

Supplement: Supplementary file 1 [file nutrients-17-00745-s001.zip › figures/vswm_e55_5-2.png]

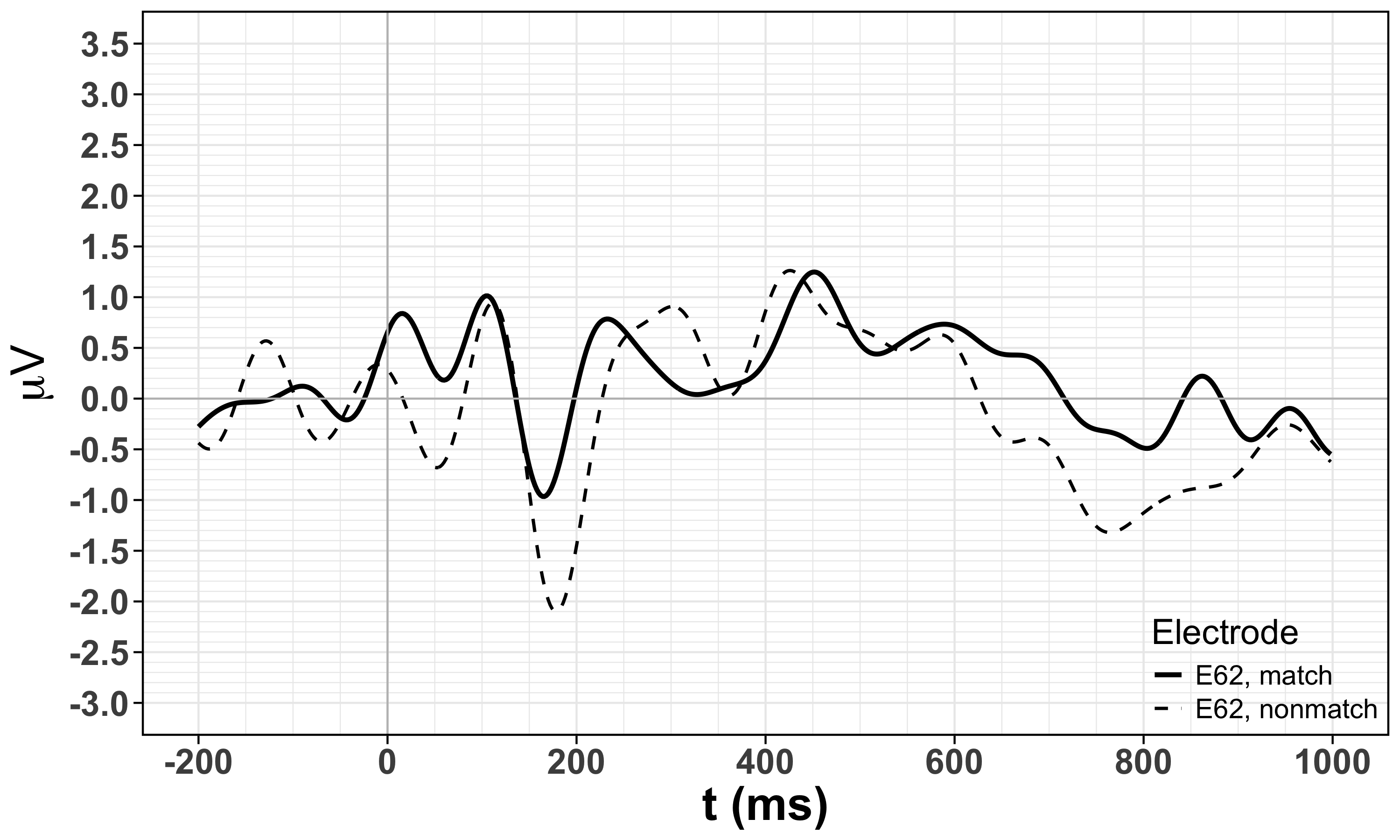

Supplement: Supplementary file 1 [file nutrients-17-00745-s001.zip › figures/vswm_e62_3-0.png]

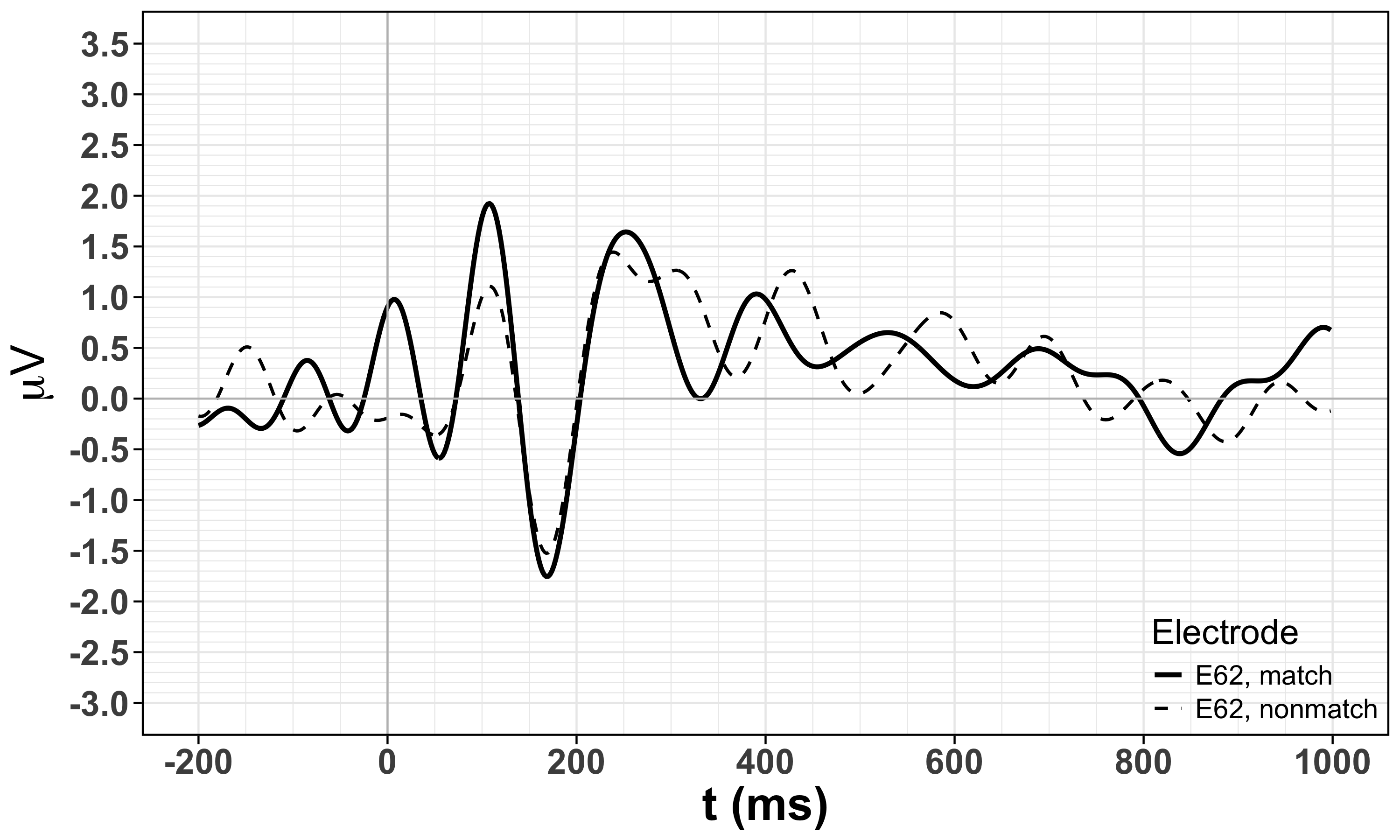

Supplement: Supplementary file 1 [file nutrients-17-00745-s001.zip › figures/vswm_e62_3-2.png]

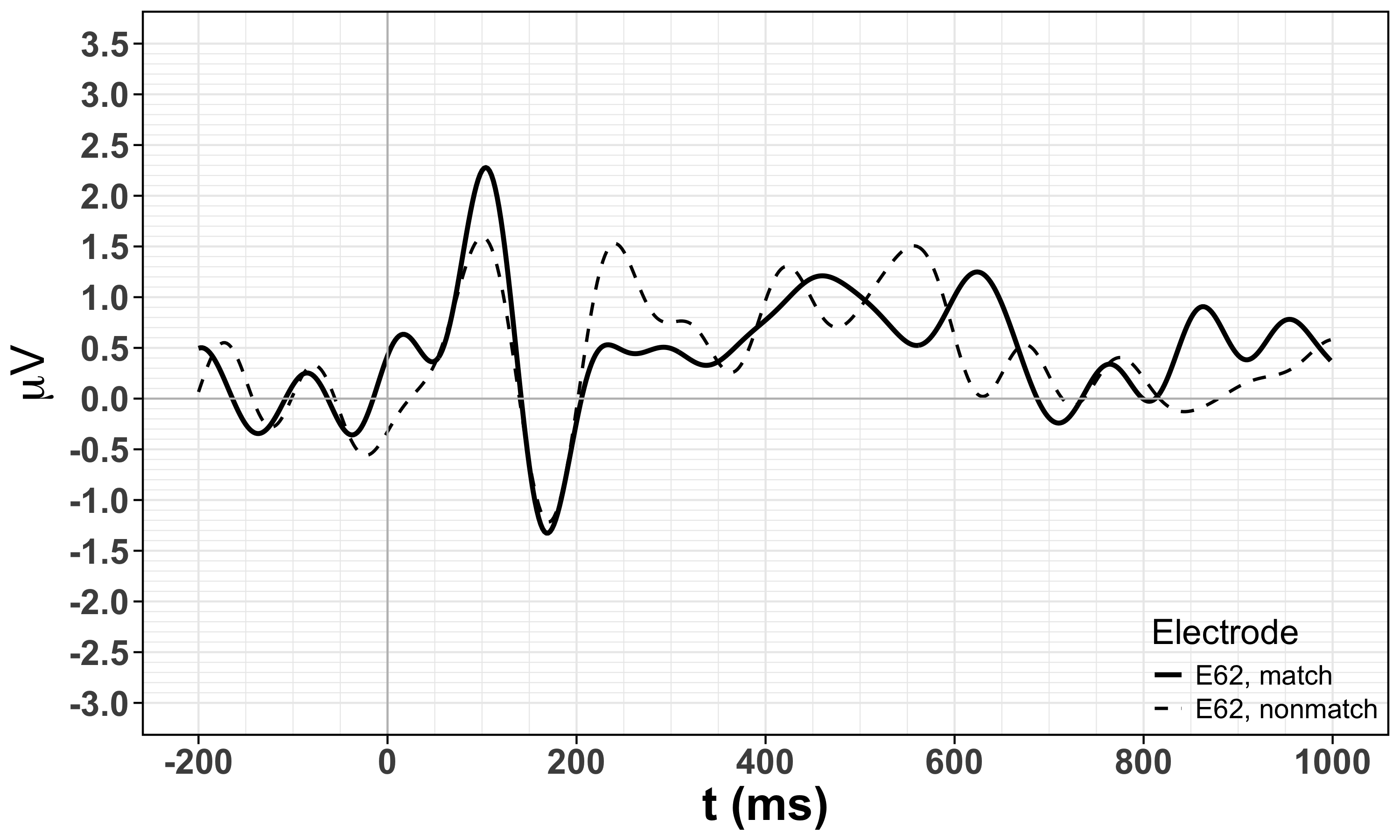

Supplement: Supplementary file 1 [file nutrients-17-00745-s001.zip › figures/vswm_e62_5-0.png]

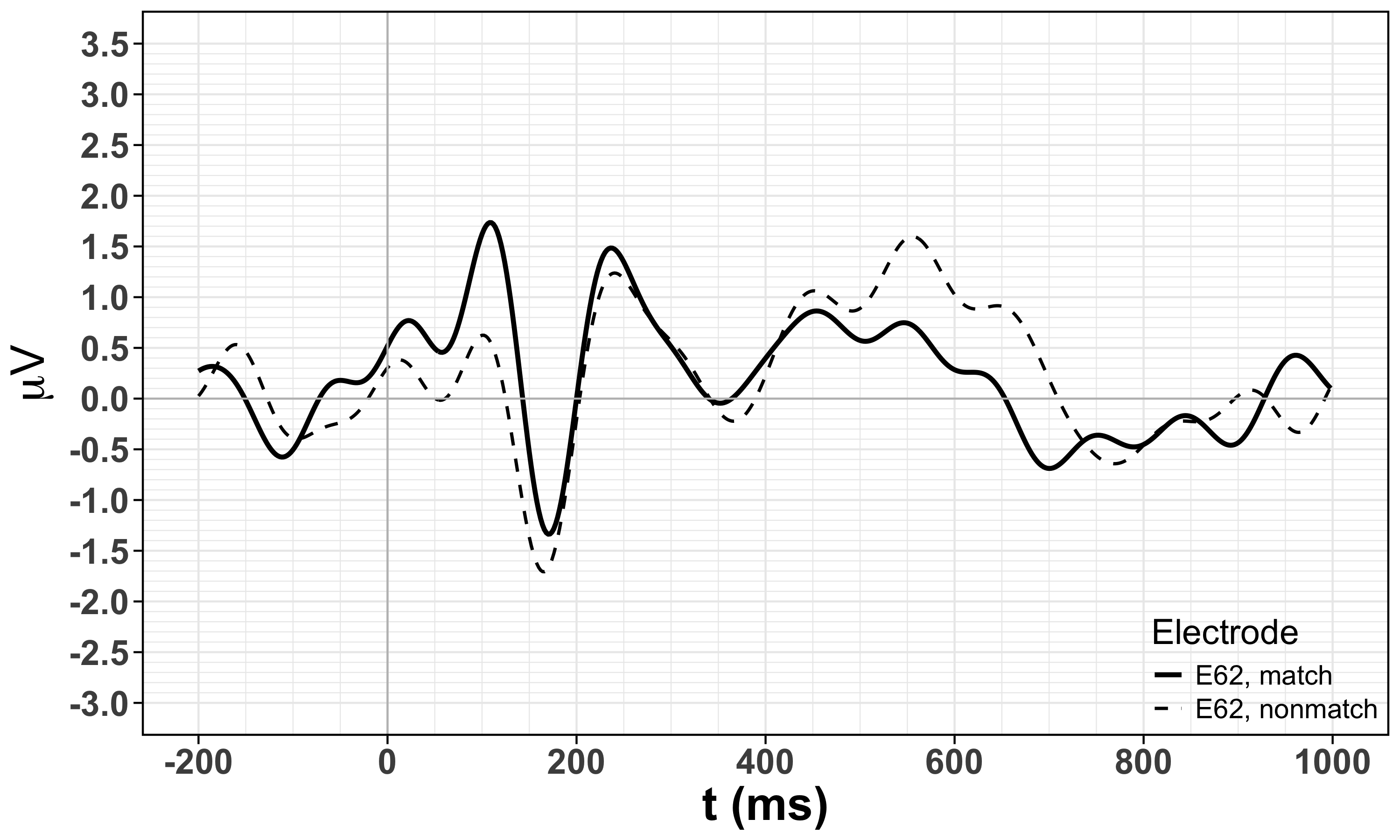

Supplement: Supplementary file 1 [file nutrients-17-00745-s001.zip › figures/vswm_e62_5-2.png]

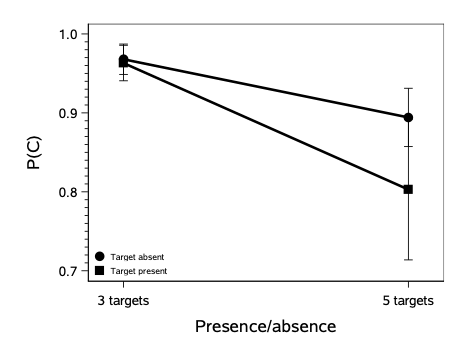

Supplement: Supplementary file 1 [file nutrients-17-00745-s001.zip › figures/vswm_interaction.png]

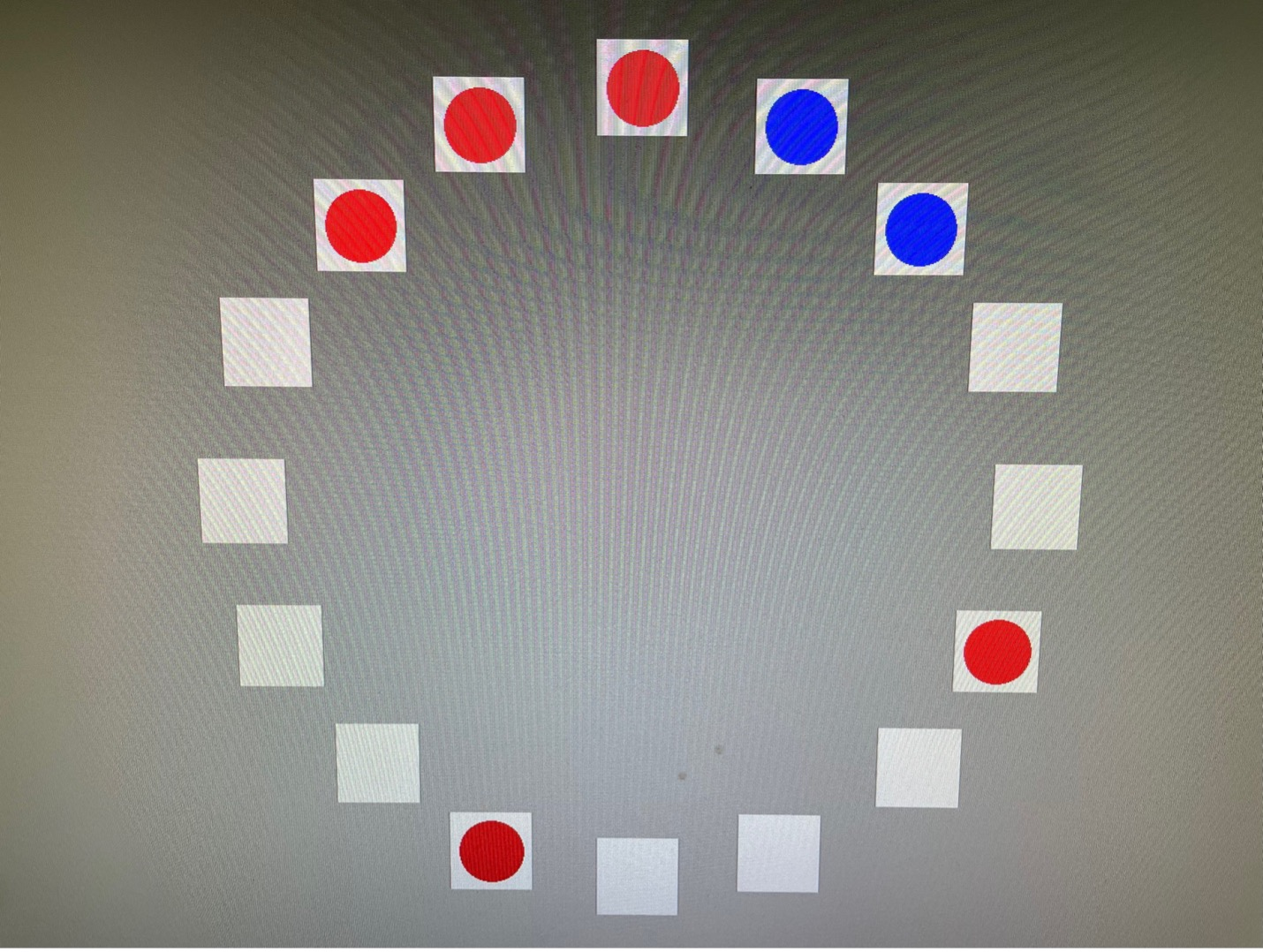

Supplement: Supplementary file 1 [file nutrients-17-00745-s001.zip › figures/vswm_stim.png]
